# Clinical Advances in Immunonutrition and Atherosclerosis: A Review

**DOI:** 10.3389/fimmu.2019.00837

**Published:** 2019-04-24

**Authors:** Ana María Ruiz-León, María Lapuente, Ramon Estruch, Rosa Casas

**Affiliations:** ^1^Department of Internal Medicine, Hospital Clinic, University of Barcelona, Barcelona, Spain; ^2^Mediterranean Diet Foundation, Barcelona, Spain; ^3^CIBER 06/03: Fisiopatología de la Obesidad y la Nutrición, Instituto de Salud Carlos III, Madrid, Spain

**Keywords:** immunonutrition, atherosclerosis, cardiovascular disease, Mediterranean diet, functional foods, dietary supplements, inflammation, bioactive compounds

## Abstract

Atherosclerosis is a chronic low-grade inflammatory disease that affects large and medium-sized arteries and is considered to be a major underlying cause of cardiovascular disease (CVD). The high risk of mortality by atherosclerosis has led to the development of new strategies for disease prevention and management, including immunonutrition. Plant-based dietary patterns, functional foods, dietary supplements, and bioactive compounds such as the Mediterranean Diet, berries, polyunsaturated fatty acids, ω-3 and ω-6, vitamins E, A, C, and D, coenzyme Q10, as well as phytochemicals including isoflavones, stilbenes, and sterols have been associated with improvement in atheroma plaque at an inflammatory level. However, many of these correlations have been obtained *in vitro* and in experimental animals' models. On one hand, the present review focuses on the evidence obtained from epidemiological, dietary intervention and supplementation studies in humans supporting the role of immunonutrient supplementation and its effect on anti-inflammatory response in atherosclerotic disease. On the other hand, this review also analyzes the possible molecular mechanisms underlying the protective action of these supplements, which may lead a novel therapeutic approach to prevent or attenuate diet-related disease, such as atherosclerosis.

## Introduction

Globally, cardiovascular diseases (CVD) represent the most frequent cause of death worldwide. It has been estimated that in 2013 17.3 million people died from this disease (1), representing 31.5% of the total deaths worldwide (2). Key factors related to maintaining cardiovascular health are to not smoke, to perform physical activity, maintain a healthy body weight with a healthy diet, and control blood lipid, blood pressure (BP) and glycemia levels to within normal values (3, 4). In fact, adherence to these factors is correlated with lower cardiovascular mortality [relative risk (RR), 0.25; 95% confidence interval (CI) 0.10–0.63] (3). In this respect, diet plays a key role. Good cardiovascular health status is related to a balanced energy intake including whole-grain foods, legumes, seafood and fish, and high content in fruits and vegetables and low intake of processed food and red meat, sugar added foods or beverages and refined grains (4, 5).

Most CVDs are associated with the development of atherosclerosis (3), which is a chronic systemic inflammatory disease that affects artery walls due to altered inflammatory response. Cholesterol-rich lipoproteins with apolipoprotein B are susceptible to absorption and binding to the arterial subendothelial matrix. In this matrix, lipoproteins are altered by oxidation, enzymatic and non-enzymatic cleavage, and aggregation, producing pro-inflammatory particles and activating the overlying endothelium. Thereafter, the recruitment of monocyte-derived cells to the subendothelium activates immune response. These cells transform into mononuclear phagocytes that engulf normal and altered lipoproteins and transform into cholesterol foam cells which remain in the plaque, take up lipids, and engorge and stimulate disease progression by developing chronic inflammatory response (6, 7).

**Figure 1:**
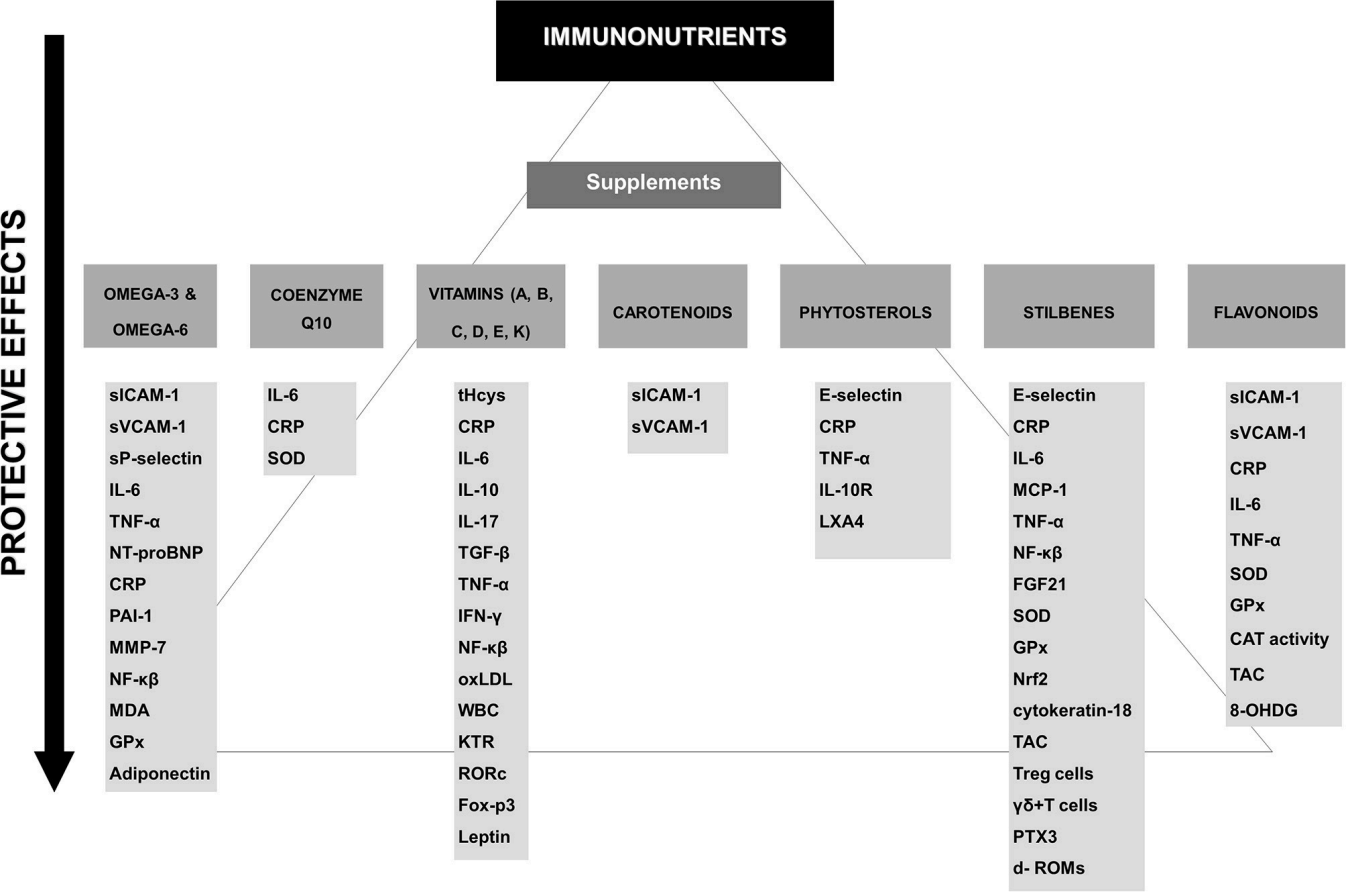
Potential protective effects of the different supplements on immune factors. CAT, catalase; CRP, C-reactive protein; d- ROMs, diacron-reactive oxygen metabolites; FGF21, Fibroblast growth factor 21; Foxp3, forkhead box protein-3; GPx, glutathione peroxidase; 8-OHDG, hydroxydeoxyguanosine; IFN-γ, Interferon gamma; IL-, interleukin; KTR, kynurenine-to-tryptophan ratio; LXA4, lipoxin A4; MDA, malondialdehyde; MCP-1, monocyte chemoattractant protein-1; MMP, metalloproteinases; NF-κβ, nuclear transcription factor signaling; NT-pro-BNP, N-terminal pro b-type natriuretic peptide; oxLDL, oxidized low-density lipoprotein; PAI-1, plasminogen activator inhibitor type 1; RORc, retinoid-related orphan receptor-c; sICAM-1, soluble intercellular adhesion molecule 1; SOD, superoxide dismutase; sVCAM-1, soluble vascular cell adhesion molecule-1; TAC, total antioxidant capacity; T-bet, T helper 1 cell lineage commitment; TGF-β, transforming growth factor-beta; TNF-α, tumor necrosis factor-alpha; WBC, white blood cell count.

Lifestyle modifications and medical treatment are the most frequent approaches to prevent clinical manifestations of cardiovascular diseases such as myocardial infarction, stroke or renal failure (3). In this sense, plant-based dietary patterns, functional foods, dietary supplements, and bioactive compounds have been associated with improvement in atheroma plaque development at an inflammatory level. However, many of these correlations have been obtained *in vitro* and in experimental animal models. Therefore, the present review focuses on the evidence obtained from epidemiological, dietary intervention and supplementation studies in humans supporting the role of immunonutrient supplementation in atherosclerotic disease. This review also analyzes the possible molecular mechanisms underlying the protective action of these supplements, which may lead to the development of novel therapeutic approaches to prevent or attenuate diet-related disease such as atherosclerosis (Figure 1). Relevant studies, systematic reviews and meta-analysis were searched to obtain the reference lists. The Medical Subject Headings search terms included: inflammation, oxidative stress, inflammatory markers, IL-1, CRP, TNF-α, IL-6, atherosclerosis, flavonols, stilbenes, coenzyme Q10, vitamins, carotenoids, omega-3 fatty acids, omega-6 fatty acids, resveratrol, catechins, epigallocatechin gallate, flavonoids, flavonols, and phytosterols. We performed a search of the MEDLINE, PUBMED, and Cochrane Library databases, and reviewed the English language literature of humans with no time restriction.

## Omega-3

Among polyunsaturated fatty acids (PUFAs), the most important classes are the omega-3 (ω-3) and omega-6 (ω-6) fatty acids (FA). PUFAs present two or more double bonds between carbons within the fatty acid chain. It is possible to distinguish several different ω-3 FA: α-linolenic acid (ALA), eicosapentaenoic acid (EPA), and docosahexaenoic acid (DHA) (8). The major ω-6 FA are linoleic and arachidonic acid (AA).

Essential FA, ALA and linoleic acid, are obtained from the diet (flaxseed, soybean, and canola oils) (9, 10). In the liver ALA is converted into EPA and then DHA (10). Both EPA and DHA can be directly obtained through diet (fish, fish oils, and krill oils) or dietary supplements and are also found in ω-3 fortified foods such as eggs, dairy products, pastas, cereals, breads and oils, among others (11).

Many chronic diseases such as CVD and cancer seem to be correlated with the ω-6/ω-3 ratio, although the optimal ratio has yet to be defined (12, 13).

There is currently a large amount of scientific evidence demonstrating the utility of marine-derived ω-3 FA supplements in the prevention of CVD. However, large studies on ω-3 FA have shown confounding results, probably because of the heterogeneous study designs (14, 15), the inclusion of mixed populations with or without coronary artery disease (CAD) (16, 17) and insufficient doses (< 1,000 mg) and duration (18) of supplementation. Indeed, a recent meta-analysis of 10 studies including 77,917 high-risk individuals (61.4% men with a mean age of 64 years) with a mean follow-up of 4.4 years did not find any significant association between ω-3 FA (226–1,800 mg of EPA acid/day) and a reduction in any major vascular events or fatal or nonfatal coronary heart disease (CHD) (19). The same results were observed in another meta-analysis performed by Rizos et al. (20). Still another meta-analysis provided insufficient evidence about the effect of ω-3 FA supplements (EPA and DHA) on the secondary prevention of CVD. The number of deaths by CVD was small (0.91; 95% confidence interval [95% CI] 0.84–0.99), and ω-3 FA did not reduce the risk of overall cardiovascular events (0.99; 95% CI 0.89–1.09) (15). On the other hand, a recent meta-analysis of 51 randomized controlled trials (RCTs) including 3,000 participants, showed a strong reduction in heart rate with ω-3 FA (DHA+EPA) supplementation. However, changes in heart rate were only observed after administering DHA alone but not after EPA alone (21).

In the last years, a great number of mechanisms have been related to the anti-inflammatory actions of ω-3 FA in atherosclerosis. Different mechanisms have been proposed in an attempt to explain the cardioprotective effects of ω-3 FA. On one hand, ω-3 FA may improve the lipid and lipoprotein profile, BP and endothelial function, and down-regulate the expression of leukocyte cells and the concentrations of various pro-inflammatory biomarkers related to the development of atherosclerosis such as chemokines, cytokines or soluble adhesion molecules as well as markers related to plaque stability such as metalloproteinases (MMP). On the other hand, mechanisms improving oxidation, thrombosis or aggregation platelet have been proposed (22–26). Thus, a recent meta-analysis including 45 RCTs and 2,674 individuals with type 2 diabetes mellitus (T2DM) linked ω-3 FA supplementation (ranging from 0.40 to 18.00 g, with duration of supplementation of 2 to 104 weeks) with a significant reduction in plasma levels of tumor necrosis factor-α (TNF-α, *P* = 0.045) and interleukin-6 (IL-6, *P* = 0.026) as well as low-density lipoprotein cholesterol (LDL-C), very low-density lipoprotein (VLDL), triglycerides (TG), and glycated hemoglobin concentrations (HbA1c)(*P* ≤ 0.044; all) (27). In addition, in another meta-analysis of 16 RCTs including 901 participants, endothelial function, measured by flow-mediated dilation (FMD), significantly improved after administering 0.45–4.5 g of ω-3 FA during 56 days (+2.30%, *P* = 0.001) (28). A systematic review of 26 RCTs (29) on ω-3 FA and inflammatory biomarkers in both healthy and ill individuals (CVD and other chronic and acute diseases) showed lower levels of inflammation [C-reactive protein (CRP), IL-6, plasminogen activator inhibitor type 1 (PAI-1), TNF-α, N-terminal pro b-type natriuretic peptide (NT-proBNP) and endothelial activation (both in healthy subjects and in those with chronic and acute diseases). Among all the ω-3 FA studied (different types and dosages), DHA showed the highest reduction in cytokine-induced endothelial leukocyte adhesion molecules (soluble intercellular adhesion molecule 1 (sICAM-1) and soluble vascular cell adhesion molecule-1 (sVCAM-1). In addition, a meta-analysis of 18 RCTs reported that ω-3 FA supplementation (0.272 to 6.6 g/d) may reduce plasma concentrations of sICAM-1 in healthy subjects (−8.87; 95% CI: −15.20, −2.53; *P* = 0.006) as well as in subjects with dyslipidemia (−15.31; 95% CI: −26.82, −3.81; *P* = 0.009) (30).

Observational studies have shown that ω-3 FA supplementation is associated with reduced markers of atherothrombotic risk. The Multi-Analyte, Thrombogenic, and Genetic Markers of Atherosclerosis study included 600 men with CVD (aged 64.4 ± 10.1 year) (31). The authors compared the use of fish oil supplementation in several subgroups: non lipid-lowering therapy vs. lipid-lowering therapy. The results showed that volunteers not receiving lipid-lowering therapy had a lower VLDL, intermediate-density lipoprotein cholesterol (IDLs), remnant lipoproteins, TG, LDL-C, oxidized low-density lipoprotein (LDL)-β2 glycoprotein complex (AtherOx) levels, collagen-induced platelet aggregation, thrombin-induced platelet-fibrin clot strength, and shear elasticity (*P* < 0.03; all).

Several mechanisms have been proposed to explain the anti-atherogenic effects of ω-3 FA on inhibiting atheroma plaque development (Table 1). In an interventional study of 275 healthy European subjects between 20 and 40 years of age, Paulo et al. (32) randomized the participants into one of four dietary groups: fish oil group (1,418 mg of ω-3 FA /day), lean fish (272 mg of ω-3 FA/day) or fatty fish (3,003 mg of ω-3 FA/day), and a control group (sunflower oil capsules). After 8-weeks of intervention sICAM-1 concentrations reduced by 5% in the lean fish group in contrast to the fatty fish and fish oil diets, in which these concentrations did not significantly change after intervention, although the latter two groups both showed a significant increase of 16.1% and 21.9%, respectively for sVCAM-1. In a randomized study (33) a significant decrease was found in sP-selectin after supplementation with 6.6 g of ω-3 FA, especially in men, while a significant reduction in sICAM-1 concentrations and an increase in sVCAM-1 concentrations were observed in women after administering 2.0 g and 6.6 g of ω-3 FA, respectively. Yusof et al. (34) also observed a slight decrease in plasma sICAM-1 concentrations after administering 1.8 g of EPA plus 0.3 g DHA daily for 8 weeks in 10 healthy middle-aged men.

**Table 1:** Nutrients and bioactive compounds can modulate the progression of atherosclerosis.

**Study and nutrient/bioactive compound**	**Study design**	**Participants**		**Findings**	**Strengths/Limitations**
**PUFA**					
Paulo et al. (32)	For 8-weeks, four randomized groups: capsules supplemented with DHA + EPA (fish oil group), lean fish (cod) or fatty fish (salmon) (3 portions of 150 g /week), and control group (sunflower oil capsules).	275 healthy subjects aged 20–40 years.	Double-blind, randomized, controlled trial.	↓ sICAM-1 in lean fish group (*P* < 0.05) ↑sVCAM-1 in fish oil and fatty fish groups (*P* < 0.05; both)	Relatively large sample size. Dietary and physical activity assessment. Comparison of foods vs. supplements/ Relatively short intervention period.
Eschen et al. (33)	Daily supplement of ω-3 FA 6.6 g, ω-3 FA 2.0 g, or olive oil during 12-weeks.	60 healthy participants (mean age 38 ± 11 y).	Double-blind, randomized, controlled trial.	Gender analysis and dietary supplementation: Men 6.6g: ↓ sP-selectin (89 ± 21 vs. 78 ± 20, *P* < 0.01) Women:↓ sICAM-1 (253 ± 38 vs. 227 ± 34, *P* < 0.05, 2.0 g) and ↑ sVCAM-1 (802 ± 198 vs. 860 ± 232, *P* < 0.05, 6.6 g).	Small sample size and low female representation. Vague description of inclusion criteria and no description of randomization method. P-selectin baseline levels were lower than 2.0 g ω-3 FA group. No information about dropout, compliance rate and dietary habits.
Yusof et al. (34)	Daily 1.8 g EPA plus 0.3 g DHA vs. placebo group (coconut oil rich in medium-chain saturated fatty acids). For 8-weeks.	Placebo group (*n* = 11) vs. Intervention group (*n* = 10). Healthy males aged 35–60 years.	Randomized, double-blinded, placebo-controlled	ω-3 FA group: −9.5 ± 6.9 ng/mL of sICAM-1 sICAM-1 concentration was inversely related to levels of DHA in plasma (*r* = −0.675; *P* < 0.001).	High compliance rate. Analysis of fatty acids composition, blood lipids levels and supplements concentration/ Small sample size and no specific limitations reported. Lower HDL-C levels at baseline in placebo group. No dietary habits assessment.
Tousoulis et al. (35)	2 g/day of ω-3 FA (dose of 2 g, 46% EPA-38% DHA) vs. placebo. For 12 weeks.	29 subjects with MetS, 14 females, aged 44 ± 12.	Randomized, placebo-controlled, double-blind, cross-over design.	ω-3 FA: ↑ FMD and PWV (*P* < 0.001 for all). ↓ IL-6 (*P* = 0.003); ↑PAI-1 (*P* = 0.003) ↓ TG and total cholesterol levels (*P* < 0.001).	Dietary assessment/ Small sample size.
Siniarski et al. (36)	Daily intake of ω-3 FA (2 g/day, 1 g of DHA and 1 g of EPA) or placebo for 3 months.	34 patients with established ASCVD and T2DM (mean age 65.6 ± 6.8 y).	Two-center, prospective randomized double-blind, placebo-controlled study.	ω-3 FA did not improve endothelial function indices (FMD and NMD).	ω-3 FA levels were measured during intervention/ Small sample size. Baseline differences in angiotensin-converting enzyme inhibitor levels. No dietary assessment.
Cawood et al. (37)	Daily intake of ω-3 FA (0.81 g EPA and 0.675 g DHA/day) or placebo for median of 21 days.	Patients awaiting carotid endarterectomy (*n* = 121), >18 years of age.	Randomized, double-blinded, placebo-controlled.	ω-3 FA group: ↓ fewer foam cells (*P* = 0.0390) ↓ mRNA for MMP-7 (*P* = 0.0055), −9 (*P* = 0.0048), −12 (*P* = 0.0044), and for IL-6 (*P* = 0.0395) and sICAM-1 (*P* = 0.0142). ↑EPA content ↓ plaque instability (*P* = 0.0209), plaque inflammation (*P* = 0.0108), the number of T cells in the plaque (*P* = 0.0097).	Relatively large sample size. Plasma FA composition was analyzed/ Short intervention period.
Thies et al. (38)	Control, sunflower oil (n−6), or fish-oil (1.4 g EPA + DHA/day) capsules for 7–189 days.	188 patients awaiting carotid endarterectomy (mean age 70 ± 8 y).	Randomized, double-blinded, placebo-controlled	ω-3 FA group: ↓ inflammation ↑ thin fibrous caps ↓ infiltration of T cells ↑inhibition of macrophages.	Large sample size and long intervention period. Low dropout rate. Dietary assessment/ Observed results depend on variable intervention time.
Zhao et al. (39)	2 g ω-3 FA (180 mg EPA and 120 mg DHA) or to matching placebo for 3 months.	76 patients with heart failure aged ≥ 60 years.	Prospective, randomized, placebo controlled study.	ω-3 FA: ↓TNF-α (*P* = 0.002), IL-6 (*P* = 0.015), sICAM-1 (*P* = 0.026), and NT-proBNP (*P* = 0.024).	Results can be only extrapolated to elder people./ Limited information about placebo characteristics. No dietary assessment.
Allaire et al. (40)	3 g/d of the following supplements for periods of 10 weeks: (1) EPA (2.7 g/d), (2) DHA (2.7 g/d), and (3) corn oil as a control for 10-weeks.	Healthy men (*n* = 48) and women (*n* = 106) with abdominal obesity and low-grade systemic inflammation.	Double-blind, randomized, crossover, controlled study.	DHA supplementation higher: ↓ CRP, IL-6, TNF-α ↑ Adiponectin.	Large sample size. High compliance. Dietary assessment/ No baseline levels of EPA and DHA in plasma phospholipids were only measured posttreatment.
Bouwens et al. (41)	For 26 weeks, daily consumption of: (1) 1.8 g EPA + DHA, (2) 0.4 g EPA + DHA, or (3) 4.0 g high–oleic acid sunflower oil.	111 healthy Dutch elderly subjects (aged > 65 years).	Double-blind, randomized, crossover, controlled study.	1.8 g EPA + DHA group changed in 1,040 genes, and changes in inflammatory pathways including eicosanoid synthesis, interleukin signaling, and MAP kinase signaling. There were also changes in the expression of genes related to atherosclerotic processes, such as cell adhesion, scavenger receptor activity, and adipogenesis, and changes in inflammatory signaling, such as eicosanoid metabolism and IL-6 and MAP kinase signaling, NF-κβ and Toll-like receptor signaling.	Large sample size. Plasma FA levels were analyzed. Gene expression analysis/ Results can be only extrapolated to elder people. Sample was not characterized.
Kusumoto et al. (42)	Arachidonic acid (AA) group and placebo group. The daily AA dose was 838 mg/d in the AA group, for 4 weeks.	24 healthy Japanese men, > 18 y, and BMI: 19–27 kg/m^2^.	Double-blind, randomized, placebo-controlled study.	No changes on any metabolic parameter or platelet function.	Accurate description of supplement composition. Dietary assessment/ Small sample size and short intervention period.
Sluijs et al. (43)	Daily intake of CLA (2.5 g c9, t11 CLA and 0.6 g trans-10, cis-12 CLA) or placebo supplements for 6 months.	401 subjects, aged 40–70 years and with a BMI ≥ 25 kg/m^2^.	Double-blind, randomized, placebo-controlled, parallel-group trial.	No changes in concentrations of fasting lipid, glucose, insulin, and CRP.	Large sample size and long intervention period. High compliance rate/ No dietary assessment.
Hassan Eftekhari et al. (44)	Daily intake of 3 g CLA, 1,920 mg/d ω-3, or placebo for 2 months.	90 atherosclerotic patients (40 males and 50 females) aged 30 to 60 years with angiographically diagnosed coronary atherosclerosis.	Double-blind, randomized, placebo-controlled, parallel-group trial.	ω-3 and CLA group: ↓ hs-CRP ↑GPx ↓MDA ω-3: ↓ IL-6.	High retention and compliance rate/ No dietary assessment.
**COENZIME Q10**					
Mohseni et al. (45)	Daily intake of 200 mg of CoQ10 or placebo for 12 weeks.	52 Iranian patients with hyperlipidemia and MI, aged 35 to 70 years old.	Randomized double-blinded controlled clinical trial.	CoQ10: ↓ total cholesterol, LDL-c, fibrinogen, TG ↑ HDL-c ↓ SBP and DBP.	Dietary assessment. High retention rate/ Small female representation (15%).
Pérez-Sánchez et al. (46)	Daily intake of 200 mg of CoQ10 or placebo for 1-month.	36 patients with antiphospholipid syndrome (mean age 51.89 ± 10.56).	Prospective, randomized, double-masked crossover, placebo-controlled study.	CoQ10 attenuated the elevated expression of inflammatory and thrombotic risk markers in monocytes.	High retention rate. MicroRNA analysis/ Short intervention period, small sample size. No dietary assessment.
Lee et al. (47)	3 dietary-arms: placebo group, 60 mg/day (Q10-60 group) and 150 mg/d (Q10-150 group) during 12-weeks.	51 patients with CAD: placebo (*n =* 14), Q10-60 group (*n* = 19), Q10-150 group (*n* = 18). Mean age 77.1 ± 5.9.	Randomized parallel, placebo-controlled study.	Q10-150 group: ↓ IL-6 and MAD Q10-60 and Q10-150 groups: ↑SOD.	Compliance was ensured/ Small sample size and female representation (8%). No information about dietary habits during intervention period.
Lee et al. (48)	Daily intake of 200 mg of CoQ10 or placebo for 12 weeks.	51 obese subjects: CoQ10 group (*n* = 26, BMI = 27.9 ± 2.3 kg/m^2^ age = 42.7 ± 11.3 years) and placebo group (*n* = 25, BMI = 26.8 ± 2.8 kg/m^2^ , age = 41.3 ± 11.2 years).	Randomized, double-blind, placebo-controlled, single center study.	No evidence that coenzyme Q10 affects fatigue index, arterial stiffness, metabolic parameters, or inflammatory markers.	Low retention rate. No information about dietary habits during intervention period.
FAITH trial (49, 50)	Placebo capsule or a capsule containing aged garlic extract and CoQ10 (extract+CoQ10, 1200 and 120 mg, respectively) daily for 1 year.	65 firefighters considered to have a high CVD risk (age 55 ± 6 years).	Placebo-controlled, double-blind, randomized trial.	Compared to placebo, extract+CoQ10: Improved coronary artery calcium (CAC) scanning (32 ± 6 vs. 58 ± 8, *P* = 0.01) Improved levels of CRP (−0.12 ± 0.24 vs. 0.91 ± 0.56 mg/L, *P* < 0.05). PWV and endothelial function measured by DTM.	Large intervention period/ The conclusions might not assess which components of garlic extract-CoQ10 capsule were responsible for the observed effects. No information about dietary habits during intervention period.
**VITAMINS**					
Christen et al. (51)	Daily combination consumption of folic acid (2.5 mg), vitamin B6 (50 mg), vitamin B12 (1 mg) or placebo for 7.3 years.	300 women with pre-existing CVD or 3 or more coronary risk factors. Mean age 62.1.	Randomized, double-blind, placebo-controlled trial.	B-vitamin group: ↓Homocysteine concentrations (−18%). No changes in CRP (*P* = 0.77), IL-6 (*P* = 0.91), ICAM-1 (*P* = 0.38), or fibrinogen (*P* = 0.68).	Participants were supplemented with folic acid. No information about dietary habits during intervention period.
Peeters et al. (52)	Daily combination consumption of 5 mg of folic acid, 0.4 mg of vitamin B12 and 50 mg of vitamin B6 or placebo for 8 weeks.	230 healthy volunteers from the general population.	Randomized, double-blind, placebo-controlled trial.	B-vitamin group: ↓Homocysteine concentrations (−18%). No changes in CRP, IL-6, IL-8, or MCP-1.	Short intervention period. No information about dietary habits during intervention period.
Van Dijk et al. (53)	Daily combination consumption of vitamin B12 (500 μg) and folic acid (400 μg) or placebo for 2 years.	522 participants elderly patients (55% were men) with hyperhomocysteinemia (12–50 μmol/l). Mean age of 72 years.	Randomized, double-blind, placebo-controlled trial.	B-vitamins group did not change compared to placebo: ICAM-1 (*P* = 0.72), VCAM-1 (*P* = 0.39), VEGF (*P* = 0.40), SAA (*P* = 0.85) or CRP levels (*P* = 0.70).	Large sample size and long intervention period. High retention rate/ Limited information about vitamin B12 levels post-intervention. No dietary assessment.
Durga et al. (54)	Daily intake of folic acid supplementation (0.8 mg/d) vs. placebo for 1 year.	530 men and postmenopausal women (aged 50 to 70 years) with homocysteine concentrations of 1.8 mg/L or higher (≥13 μmol/L) at screening.	Randomized, double-blind, placebo-controlled trial.	28% decrease in homocysteine concentrations No changes in CRP, sICAM-1, oxLDL, IgG and IgM against oxLDL.	Large sample size and long intervention period. Dietary assessment. High compliance. Low dropout rate. Plasma folate analysis.
Bleie et al. (55)	Daily intake of: (A) folic acid (0.8 mg)/vitamin B12 (0.4 mg)/vitamin B6 (40 mg), (B) folic acid/vitamin B12, (C) vitamin B6 alone or (D) placebo for 6 months.	90 patients (21 female) with CAD, aged 38–80 years.	Prospective randomized double-blind study.	Homocysteine-lowering therapy with B-vitamins did not change concentrations of: sCD40L, IL-6, CRP or neopterin.	Small sample size. Low dropout rate. Baseline analysis of vitamin B6 levels/ Small female representation.
Ulvik et al. (56)	Daily intake of: (1) 40 mg pyridoxine hydrochloride (vitamin B6) + 0.8 mg folic acid + 0.4 mg B12, (2) 0.8 mg folic acid + 0.4 mg B12, (3) 40 mg pyridoxine hydrochloride, and (4) placebo.	3,090 healthy participants (81.4% was men), the mean age 61.6 years.	Randomized, double-blind, placebo-controlled trial.	Vitamin B6 was negatively associated with CRP, WBC, KTR, and neopterin at baseline and with CRP and KTR at day 28.	Large sample size. High compliance/ Short intervention period. High dropout. No dietary assessment.
Mottaghi et al. (57, 58)	Two randomly allocated groups (vitamin A or placebo): Patients and controls with a daily intake of 25,000 IU retinyl palmitate, and patients in the placebo group for 4-months.	31 atherosclerotic patients (16 men and 15 women, aged 38–69 years; mean age 56 years) and 15 healthy controls (8 men and 7 women, aged 39–62 years; mean age 56.5 years).	Double-blind, placebo-controlled trial.	Patients with vitamin A-supplemented ↑Fox-p3 expression (*P* = 0.0001) ↑TGF-β gene expression (*P* = 0.001) ↓IL-17 (*P* < 0.05) and RORc gene expression (*P* = 0.0001).	Vitamins A intake was determined at baseline. Gene expression analysis/ Small sample size.
Sezavar et al. (59)	Healthy controls and patients in the vitamin A group received 25000 IU retinyl palmitate daily for 4 months. Control patients also received placebo per day up to 4 months.	31 patients and 15 healthy controls.	Double-blind, placebo-controlled trial.	Vitamin A intake: ↓IFN-γ gene expression in healthy control subjects (*P* = 0.0001) and atherosclerotic patients (*P* = 0.001).	Small sample size. No characterization of disease stage in patients group.
Salonen et al. (60)	(1) 91 mg of d-α-tocopherol twice daily; (2) 250 mg slow-release ascorbic acid twice daily; (3) both d-α-tocopherol and slow-release ascorbic acid and (4) Placebo for 3-years.	520 hypercholesterolemia smoking and nonsmoking men and postmenopausal women aged 45–69 years.	Clinical placebo-controlled two-by-two factorial trial.	Individual supplementation with vitamin E or C had no effect on the atherosclerosis progression in either men or women. Combined supplementation led a delay in the atherosclerosis progression (0.011 mm/ year).	Large sample size and intervention period. Relatively high retention and adherence rate./ Plasma vitamins levels were not determined. No dietary assessment.
Ellulu et al. (61)	Twice a day of 500 mg vitamin C or placebo during 8-weeks.	64 obese patients who were hypertensive and/or diabetic and had high levels of inflammatory markers, aged 50.6 years.	Open-label, parallel, randomized controlled trial.	Vitamin C group: ↓ hs-CRP, IL-6, fasting blood glucose and TG (overall: *P* < 0.001).	Compliance was ensured/ No dietary assessment. Plasma vitamin C levels were not determined.
Woollard et al. (62)	First, split into two groups based on their vitamin C status at baseline (< 50μM referred to reduced levels or ≥ 50μM referred to normal levels of vitamin C). Each subject received dietary supplements of 250 mg/day vitamin C or placebo for 6-weeks.	40 healthy non-smokers male volunteers, between 20 and 45 years (mean age 30).	A randomized double-blind crossover study.	Significant reduction in adhesion monocytes to ECs was observed from 0.88 ± 0.09 FU to 0.65± 0.11 FU after 6 weeks of supplementation with 250 mg vitamin C in the group with below average plasma vitamin C concentrations at baseline (*P* < 0.02).	Plasma vitamin C levels were determined/ Small sample size and short intervention period. No dietary assessment. No information about adherence and retention rate.
Bruunsgaard et al. (63)	(1) 91 mg of d-α-tocopherol twice daily; (2) 250 mg slow-release ascorbic acid twice daily; (3) both d-α-tocopherol and slow-release ascorbic acid and (4) Placebo for 3-years.	520 hypercholesterolemia smoking and nonsmoking men and postmenopausal women aged 45–69 years.	Clinical placebo-controlled two-by-two factorial trial.	No changes in circulating levels of TNF-α, IL-6 or CRP.	Large sample size and long intervention period/ No dietary assessment. No information about retention rate and supplementation adherence.
Mullan et al. (64)	Twice daily intake of 250 ml beverages containing 361 mg of (poly)phenols and 120 mg of vitamin C or placebo (no polyphenol/vitamin C) for 4-weeks.	39 healthy overweight or obese subjects (BMI > 25 kg/m^2^) and mean age 61.3 ± 4.4 y.	Randomized, double blind, placebo- controlled design.	No changes in markers assessed: leptin, apolipoproteins, cystatin C, insulin, adiponectin, CRP, ICAM-1, E-Selectin or t-PA. Compared to placebo IL-6 was increased in intervention group (0.32 vs. −0.18 pg/ml; *P* = 0.010). No differences between intervention group and placebo in PWV, SNP, and Ach.	Dietary assessment. High compliance/ Small sample size and short intervention period.
Gutierrez et al. (65)	Daily intake of: (1) placebo C, (2) low-dose vitamin C (250 mg/day), (3) medium-dose vitamin C (500 mg/day), and 4) high-dose vitamin C (1,000 mg/day) for two weeks.	8 volunteers (4 males, 4 females) noninsulin-requiring type 2 diabetes. Mean age was 49 ± 6 years.	Randomized, crossover, dose-response trial.	No changes on lipid profile, markers of oxidative stress, inflammation or hypercoagulability for any dosage of vitamin C.	Plasma vitamin C levels were measured/ Small sample size and short intervention period. Intervention was not blinded.
Dewell et al. (66)	Daily intake of: (1) usual diet with placebo; (2) usual diet and antioxidant supplements, and (3) antioxidant-rich foods for 8-weeks.	88 healthy adults with ≥1 elevated risk factor for cardiovascular disease. Mean age 51 ± 10 years.	Single-blind (diets)/double-blind (supplements), parallel-group study.	There were no significant changes in MCP-1, IL-6, or sICAM-1 among the 3 arms of the study.	Comparing food intake *vs*. supplements. High retention rate and adherence. Dietary assessment/ Relatively small sample size and short intervention period. Only diet group was blinded.
Beilfuss et al. (67)	The subjects were randomized into three groups: (1) 20,000 IU vitamin D (cholecalciferol) per week; (2) 40,000 IU vitamin D (cholecalciferol) per week; (3) Placebo. During 1-year.	332 healthy males and females 21–70 years old, with BMI between 28.0 and 47.0 kg/m^2^.	Placebo-controlled, double-blind, randomized trial.	Compared with placebo vitamin D supplementation: ↓ IL-6 and ↑ hs-CRP No changes in TNF-α and insulin resistance.	Large sample size and long intervention period. High compliance. Quantification of serum 25(OH)D levels/ Study groups received also calcium supplements. No information about dietary and exercise habits.
Tabesh et al. (68)	(1) 50,000 IU/wk vitamin D + calcium placebo; (2) 1000 mg/d calcium + vitamin D placebo; (3) 50,000 IU/wk vitamin D + 1000 mg/d calcium; or (4) vitamin D placebo + calcium placebo for 8 weeks.	118 Iranian patients with type 2 diabetes.	Double-blind, parallel, randomized placebo-controlled clinical trial.	Calcium, vitamin D, vitamin D+calcium: ↓IL-6, TNF-α, leptin vitamin D+calcium: ↓ hs-CRP.	Quantification of serum 25(OH)D levels. High compliance. Dietary and exercise assessment/ The study was conducted in summer. Relatively short intervention period.
Schleithoff et al. (69)	D (+) group received a daily supplement of 50 μg (2000 IU) cholecalciferol vs. the D(–) group received a placebo and cholecalciferol for 9 months.	123 congestive heart failure (CHF) patients (102 men and 21 women).	Double-blind, parallel, randomized placebo-controlled clinical trial.	D(+) group: ↓ TNF-α ↑ IL-10.	Relatively large sample size. Dietary assessment/ Optimal plasma vitamin D levels were not reached. High dropout rate. Calcium supplementation might have influenced cardiac function in both study groups.
Mousa et al. (70)	Daily intake of: (1) 100,000 IU of vitamin D; (2) 4,000 IU of vitamin D, or (3) placebo group for 16 weeks.	65 Australian overweight or obese, vitamin D-deficient (25-hydroxyvitamin D ≤ 50 nmol/L) adults. Aged 18–60 years.	Parallel-group, double-blind, randomized, placebo-controlled trial.	No differences were observed between groups (vitamin D and placebo) in any inflammatory markers or NF-κβ activity (all *P* > 0.05).	Detailed confounders description and analysis. High compliance. Dietary assessment/ Insulin sensitivity was calculated through sample size. Optimal plasma vitamin D levels were not reached (80–100 nmol/L). High dropout.
Waterhouse et al. (71)	Daily intake of: placebo, 750 μg or 1,500 μg of vitamin D for 1-year.	615 participants aged between 60- and 84-year-old.	Randomized, placebo-controlled, double-blind trial.	No differences were observed between groups (vitamin D and placebo) in any inflammatory markers or adipokines studied.	Large sample size and long intervention period. Two supplement doses. Good compliance rate. Baseline dietary vitamin D intake analysis/ No comparably to other populations. No fasting blood sampled were used. No dietary assessment during intervention period.
Plantinga et al. (72)	Daily intake of combined vitamin C (1g) and vitamin E (400 IU) or placebo, for 8 weeks.	30 never-treated, male, essential hypertensive patients (mean age, 50 years).	Randomized, double-blind, placebo-controlled, crossover study design.	Combined antioxidants: ↑ FMD ↓ PWV and Aix ↓ MDA, LOOH, FRAP ↑ Antioxidant capacity.	Combined vitamins supplements. Dietary habits and physical activity assessment. Vitamins plasma level analysis/ Small sample size. No dropout information.
Magliano et al. (73)	Daily intake of 500 IU of vitamin E or placebo for 4 years.	409 Australian male and female smokers aged 55 years without previously reported CVD.	Randomized, double-blind, placebo-controlled trial.	Vitamin E: ↓LDL oxidative susceptibility Not reduction the progression of carotid atherosclerosis.	Large sample size and long intervention period. Compliance was ensured/ Baseline vitamin E group showed higher BMI and different treatment. No dietary habits assessment.
Devaraj et al. (74)	Daily intake of high intake of α-tocopherol 1,200 IU or placebo for 2 y.	90 patients with CAD. Age of 40–70 y.	Randomized, controlled, double-blind trial.	Vitamin E: ↓ F(2)-isoprostanes (*P* < 0.001) ↓TNF-α, IL-6 (*P* < 0.005) and monocyte superoxide anion (*P* < 0.001) ↓ hs-CRP (−32%) vs. placebo; *P* < 0.001.	Relatively long intervention period. High compliance. Plasma tocopherol levels analysis/ No dietary habits assessment.
Wu et al. (75)	Daily intake of 500 mg of: (1) alpha-tocopherol; (2) mixed tocopherols rich in gamma-tocopherol, or (c) placebo for 6 weeks.	55 patients with type 2 diabetes. Mean age: 61.3.	Double-blind, placebo-controlled trial.	Tocopherol groups: ↓ F(2)-isoprostanes (*P* < 0.001) ↑Neutrophil alphaT and gammaT increased (both *P* < 0.001) ↓Neutrophil leukotriene B(4) production decreased significantly in the mixed tocopherol group (*P* = 0.02) No changes in hs-CRP, IL-6, TNF-α or MCP-1.	*In vitro* analyses were performed. High compliance/ Small sample size and short intervention period. A mixed tocopherols supplement was used. No dietary habits assessment.
Gutiérrez et al. (76)	Daily intake of placebo, low-dose (200 IU/d), medium-dose (400 IU/d), and high-dose vitamins (800 IU/d) for two weeks.	6 males and 5 females with T2DM.	Randomized placebo-controlled, crossover trial.	Low-dose of vitamin E No changes in glutathione, CRP, adiponectin, PAI-1, and fibrinogen levels. ↓ oxLDL production.	Small sample size and short intervention period/ Different dosage analysis.
Knapen et al. (77)	Daily intake of 180 μg of menaquinone vs. placebo for 3-years.	244 healthy postmenopausal women aged between 55 and 65 years.	Randomized, double- blind, placebo-controlled	Menaquinone group: ↓ Stiffness Index β No effect on hsCRP, IL-6 and TNF-α or on VCAM, E-selectin and AGE.	Large sample size and long intervention period. Low dropout rate/ Bone strength was used to calculate the sample size. No dietary habits assessment. Phylloquinone blood concentrations were not analyzed.
Kristensen et al. (78)	Daily intake of 500 μg of phylloquinone or placebo for 6-weeks.	48 healthy postmenopausal women (>5 years postmenopausal). Mean age 62.5 ± 4.0 y.	Randomized double-blind crossover study.	Menaquinone group: HDL-c decreased by 5% (*P* = 0.006) and triacylglycerols by 15% (*P* = 0.015). No changes in sICAM-1, sVCAM-1, PAI-1, fibrinogen and plasma factor VIIc.	Small sample size. Dietary habits assessment. Blood phylloquinone levels were analyzed/ Dietary phylloquinone intake was not estimated during intervention period. High dropout rate.
Shea et al. (79)	Daily multivitamin with 500 μg phylloquinone or a daily multivitamin without phylloquinone for 3-years.	388 healthy men and postmenopausal women aged 60–80 y.	Double-blind, randomized controlled trial.	Phylloquinone supplements reduced CAC progression by 6% (*P* = 0.04).	Large sample size and intervention period/ Results are not comparably to others populations.
**CAROTENOIDS**					
Colmán-Martínez et al. (80)	200 mL or 400 mL of tomato juice for 4-weeks.	28 participants at high cardiovascular risk (mean age = 69.7 years, BMI 31.5 ± 3.6 kg/m^2^).	Prospective, randomized, cross-over, and controlled clinical trial.	Both tomato juices: ↓ ICAM-1, VCAM-1 (these changes were correlated with trans-lycopene levels). No significant changes: CRP, IL-8, eotaxin, IFN-γ and CXCL10.	Dietary assessment. Analysis of plasma carotenoid levels/ Small sample size and short intervention period. High dropout rate.
Stonehouse et al. (81)	Palm carotene (21 mg of carotenes) for 8-weeks.	90 participants with type 2 diabetes. Aged between 18 and 70 years, and BMI between 20 and 45 kg/m^2^.	Double-blind, randomized, placebo-controlled trial.	No significant changes were observed: - Physiological markers of vascular function: FMD, PWV, and AI - Circulatory markers of vascular function: serum ICAM1, VCAM-1, E-selectin, plasma tPA and PAI-1 - Inflammatory markers: serum hsCRP, TNFα, IL-6, adiponectin.	High compliance. Plasma carotene and tocotrienol analysis. Relatively short intervention period. Sample diversity. Baseline differences (BP, lipid lowering drugs).
Coombes et al. (82)	12 mg astaxanthin/day for 12-months.	61 renal transplant recipients. Mean age 49.9 and BMI 26.9 kg/m^2^.	Double-blind, randomized, placebo-controlled trial.	No significant changes were observed: - PWV, CIMT, FMD, GTN, F2-isoprostanes, pentraxiω-3, CRP.	Relatively long intervention period. High compliance and subject retention. Astaxanthin blood level analysis. No dietary habit assessment.
Zou et al. (83)	20 mg lutein/day or 20 mg lutein/day + 20 mg lycopene/day for 12-months.	144 participants with subclinical atherosclerosis. Aged between 45 and 68 years and average BMI 24.7 kg/m^2^.	Double-blind, randomized, placebo-controlled trial.	↓ carotid artery IMT (lutein+ lycopene > lutein alone).	Relatively large sample size and long intervention period. Lutein serum levels analysis. Dietary habit assessment. High compliance.
**PHYTOSTEROLS**					
Lambert et al. (84)	Phytosterol-milk (1.6 g of plant sterols/250 mL of milk) or ω-3-milk (131.25 mg EPA + 243.75 mg DHA/250 mL of milk) for 4-weeks.	32 participants with overweight or obesity (BMI 25–35 kg/m^2^). Aged between 25 and 70 years.	Double-blind, randomized, crossover longitudinal trial.	Phytosterol-milk: ↓ expression of inflammatory molecules (MCP-1, IL-10R).	Compliance was checked. Small sample size and short intervention period.
Ho et al. (85, 86)	Two soymilk (20 g) treatments daily. 2.0 g free plant sterols equivalent of their palmitates (β-sitosterol, 55%; campesterol, 29%; stigmasterol, 23%) for 4-weeks.	18 healthy adults (67% female). Mean age 35.3 years.	Double blind, randomized, placebo-controlled crossover study.	Plant sterols treatment: ↓ lipid peroxidation, and inflammation: ↓ plasma hsCRP, ↑ LXA4, nitrite and nitrate.	Complemented with *in vitro* analysis. Small sample size and short intervention period. No information about FMD.
Heggen et al. (87)	Two sterol margarines (2 g phytosterol/day) and a control non-sterol margarine for 4-weeks.	58 volunteers with hypercholesterolemia. Aged between 25 and 75 years. BMI < 29 kg/m2.	Double-blind, randomized, placebo-controlled crossover trial.	Rapeseed-sterol margarine: ↓ E-selectin.	Food intervention. Small sample size and short intervention period. Limited information about double-blind process.
Ras et al. (88, 89)	Margarine supplemented with 3 g of phytosterol/day or placebo for 12-weeks.	240 participants with hypercholesterolemia. Aged between 40 and 65 years. BMI: 18–30 kg/m^2^.	Double-blind, randomized, placebo-controlled, parallel-group study.	Biomarkers of endothelial dysfunction and low-grade inflammation were not modified (CRP, serum amyloid A, IL-6, IL-8, TNF-α, and soluble intercellular adhesion molecule-1) and neither was FMD.	Relatively large sample size. High compliance. Phytosterol plasma level analysis. No dietary habit assessment. FMD significantly different among groups at baseline.
Macedo et al. (90)	100 mg/day *trans*-resveratrol (before and after a habitual physical fitness test) for 3-months.	60 healthy military firefighters. Mean age 21.88 years.	Double -blind, randomized, placebo-controlled trial.	Before physical fitness test: ↓ GPx After physical fitness test: ↑ plasma glucose, TG, ↓ TNF-α, GPx No significant changes vs. placebo group: - TC, LDL-C, HDL-C, AST, ALT, GGT plasma activities, LDH, serum iron, creatinine, uric acid, total plasma antioxidant activity (FRAP) - Plasma oxidative stress biomarkers: thiol, 8-isoprostane, 8OHdG - Pro-inflammatory cytokines: IL-1β, IL-6 - Antioxidant enzyme activities: SOD, catalase, glutathione reductase.	Homogeneous sample group. No withdrawal. Healthy group population. No dietary habit assessment.
Espinoza et al. (91)	1,000 mg/day resveratrol for 7-weeks.	9 healthy participants. Aged between 30 and 50 years and BMI 20 kg/m^2^.	Randomized clinical trial.	↑ total antioxidant capacity Circulating immune cells: ↑ circulating Treg cells (at 4 weeks), γδ+T cells (at 4 and 6 week). ↓ TNF-α, MCP-1 (at 4 weeks, both) No significant changes: bitem[-] CXCL-10, IL-1Ra.	Complementation with cell culture analysis. Small sample size, not blinded, control group did not take placebo, dietary habits during intervention were not reported.
Made et al. (92, 93)	150 mg trans-resveratrol/day for 4-weeks.	45 participants with obesity or overweight. Mean age 61 years. BMI 28.3 kg/m^2^.	Double-blind, randomized, placebo-controlled trial.	↑ DBP, heart rate No significant changes: - FMD, DBP, mean arterial pressure. - Fasting arterial diameters, arterial stiffness after test meal - TC, LDL-C, HDL-C, TC/HDL ratio, TG, apoA-I, apoB100 - BMI, plasma glucose, insulin, HOMA-IR - Markers of inflammation and endothelial function: hsCRP, IL-6, TNFα, E-Selectin, thrombomodulin, *P*-Selectin, ICAM-3, sICAM-1, sVCAM-1.	Dietary assessment. High compliance. Small sample size and short intervention period.
Kitada et al. (94)	20 mg/day piceatannol for 8-weeks.	39 participants with obesity or overweight. Aged between 20 and 70 years and BMI > 25 kg/m^2^.	Double-blind, randomized, placebo-controlled trial.	Male participants with overweight: ↓ serum insulin, HOMA-IR No significant changes: - Body weight, BMI, body composition - Fasting glucose, insulin, HOMA-IR, HbA1c, glycated albumin - AST, ALT and GGT, serum creatinine, glomerular filtration rate, and serum uric acid, TC, LDL-C, HDL-C, TG and free fatty acid levels - FMD, asymmetric dimethylarginine - Serum hs-CRP, IL-6, diacron reactive oxygen metabolite (dROM) and biological antioxidant potential - Sirt1 and *P*-AMPK expression.	PBMC gene expression analysis. Small sample size (sub-group analysis) and small intervention period. No dietary assessment.
Kjær et al. (95)	1,000 mg resveratrol/day (high) or 150 mg resveratrol/day for 16-weeks.	74 men with MetS. Mean age 49.5 and BMI 33.8 kg/m^2^.	Double-blind, randomized, placebo-controlled trial.	High dose resveratrol: ↑ TC, LDL-C, fructosamine No significant changes (both doses): - hs-CRP - IL-6, soluble urokinase plasminogen activator receptor.	Two intervention dosages. Compliance was checked/ Small sample size, no information on plasma resveratrol levels.
Bo et al. (96, 97)	500 mg resveratrol/day or 40 mg resveratrol/day for 6-months.	192 participants with type 2 diabetes. Mean age 66 years, BMI < 35 kg/m^2^.	Double-blind, randomized, placebo-controlled trial.	↑ dose-dependent PTX3 ↑ total antioxidant status High dose: ↑ TC No significant changes (both doses): - CRP, IL-6, C-peptide - fasting glucose glycated hemoglobin, insulin, free fatty acids, liver transaminases, uric acid.	Two intervention dosages. Dietary habits assessment. Higher female representation in 40 mg intervention group. No plasma resveratrol levels analyzed.
Seyyedebrahimi et al. (98)	800 mg/day resveratrol for 2 months.	48 Participants with type 2 diabetes. Aged between 30 and 70 years and average BMI 28.94 kg/m^2^.	Double-blind, randomized, placebo-controlled trial.	↓ plasma protein carbonyl content and PBMCs O2- ↑ plasma total antioxidant capacity and total thiol content ↑ Nrf2, SOD expressions ↓ SBP, DBP, weight, BMI.	Compliance was checked/ Small sample size. No dietary habit assessment during intervention period.
Imamura et al. (99)	100 mg resveratrol tablet/day (total resveratrol: oligo-stilbene 27.97 mg/100 mg/day) for 12-weeks.	50 participants with type 2 diabetes. Mean age 57.8 years and BMI 25.1 kg/m^2^.	Double-blind, randomized, placebo-controlled trial.	↓ Cardio-ankle vascular index (CAVI) ↓ diacron-reactive oxygen metabolites (d- ROMs) No significant changes: - Fasting plasma glucose, HbA1c, TC, TG, HDL-C and LDL-C - Weight, BMI, DBP, SBP.	No dropouts. Small sample size and relatively short intervention period. No previously established reference CAVI cut-off value. Limited information about dietary habits modification.
Chen et al. (100)	300 mg/day resveratrol for 3-months.	60 patients with non-alcoholic fatty liver disease. Mean age 44.3 and BMI 25.7 kg/m^2^.	Double-blind, randomized, placebo-controlled trial.	↓ TNF-α, adiponectin, FGF21, cytokeratin 18 ↓ TC, LDL-C ↓ ALT, AST ↓ glucose, HOMA-IR No significant changes: - Weight, BMI, waist and hip circumference, waist:hip ratio, SBP, DBP - Red blood cells, WBC, hemoglobin, platelet, blood urea nitrogen, creatinine, GGT, insulin, C-peptide, TG, HDL-C, Apo B, Apo A-I.	Compliance was checked/ Ultrasound diagnosis. Not enough dietary habits control during intervention period.
Heebøll et al. (101)	1,500 mg resveratrol/day for 6-months.	28 patients with non-alcoholic fatty liver disease. Aged between 18 and 70 years and BMI ≥ 25 kg/m^2^.	Double -blind, randomized, placebo-controlled trial.	↓ GGT, DBP, TG No significant changes: - ALT, AST, alkaline phosphatase, CD163, TNFα - weight, BMI or waist-hip ratio, SBP, heart rate, fasting glucose, insulin, HOMA-IR, TG, LDL-C, HDL-C.	mRNA expression analysis in hepatic tissue/ Small sample size, minimum target size was not met.
Faghihzadeh et al. (102)	500 mg/day resveratrol for 12-weeks.	50 patients with alcoholic fatty liver disease, ≥ 18 years and mean BMI 28.55 kg/m^2^.	Double-blind, randomized, placebo-controlled trial.	↓ ALT ↓ hs-CRP, TNF-α, IL-6, and NF-κB, cytokeratin-18 No significant changes: - Weight, BMI, waist circumference, hip circumference, waist-to-hip ratio. - AST, GGT, bilirubin direct, bilirubin total.	High compliance, dietary habit assessment/ Small sample size.
**ISOFLAVONES**					
Sathyapalan et al. (103)	SPI group (15 mg soy protein with 66 mg of isoflavones). SP group (15 g soy protein alone, isoflavone free) for 6-months.	200 women within two years of the onset of menopause (FSH > 20 mU/L and amenorrhea for 1 year). Non-smokers and non-T2DM.	Double-blind, randomized parallel study.	↓ SBP ↓ CHD, MI CVD and CVD death risk No significant changes in DBP and lipid profile, hs-CRP.	Relatively large sample size. High compliance/ High dropout rate and no dietary habit assessment.
Hodis et al. (104)	25 g soy protein containing 91 mg aglycon isoflavone equivalents or placebo for 2.7-years.	350 postmenopausal women (>1 year) and serum estradiol < 20 pg/mL. No DM, no CVD.	Double-blind, placebo-controlled trial.	ISP supplementation over 2,7-year period did not significantly reduce the progression of subclinical atherosclerosis in postmenopausal women.	Long intervention period and large sample size. High compliance/ Exclusion criteria do not specifically include taking soy supplementation. No withdrawal reason reported.
Byun et al. (105)	Chungkookjang group (35 g freeze-dried Chungkookjang/daily) and placebo group for 12-weeks.	120 students (men and women) between 19 and 29 years of age, overweight/obese.	Double-blind, randomized, controlled crossover trial.	Women: ↓ % body fat, lean body mass, waist circumference, waist-to-hip ratio. Improved lipid profile. Men: Apo B/Apo A1 ratio improvement.	Relatively high sample size. Dietary habit and exercise assessment/ Young sample (low inflammation).
Back et al. (106)	Chungkookjang group (26 g of freeze-dried Chungkookjang daily) and placebo group for 12-weeks.	55 overweight/obese male and female subjects not diagnosed with any disease.	Randomized, double-blind, placebo-controlled clinical trial.	No significant changes in abdominal fat and plasma lipids.	Small sample size. No dietary habit or exercise assessment
Chan et al. (107)	Isoflavone supplement group (80 mg isoflavone/daily) and placebo group for 12-weeks.	102 patients with primary or recurrent ischemic stroke (>6 months).	Randomized, double-blind, placebo-controlled trial.	↓ serum hs-CRP ↑ FMD in patients with clinically manifest atherosclerosis No changes on NMD, BP, heart rate, glucose and insulin levels, HbA1c, SOD, 8-isoprostane, and MDA.	Relatively large sample size. Dietary habit assessment. Limited information about similarities between two groups at baseline.
Törmälä et al. (108)	Soy powder group (52 g of soy protein containing 112 mg isoflavones expressed as aglycone) and placebo powder group (52 g of milk protein) for 8-weeks.	40 healthy non-smoking postmenopausal women on tibolone treatment (≥3 months).	Randomized, placebo-controlled cross-over trial.	Soy supplementation did not improve vascular function in either equol producers or non-equol producers. The capacity to produce equol seems to be an independent determinant of vascular health in tibolone users.	Difference between equol producers and non-equol producers. Compliance was checked and genistein levels were analyzed/ Small sample size. No dietary habits assessment. Only generalizable to tibolone treated women.
Fuchs et al. (109)	Isoflavone-enriched cereal bar group (50 mg isoflavone/daily) and placebo group for 8-weeks.	27 healthy postmenopausal woman between 45 and 70 years of age.	Placebo-controlled sequential design.	Soy isoflavones may increase anti-inflammatory response in blood mononuclear cells that might contribute to the atherosclerosis-preventive activities of a soy-rich diet.	Proteomic approach/ Small sample size. Low inflammatory levels in healthy women. No dietary habit assessment.
**FLAVONOLS**					
Brüll et al. (110)	162 mg/day quercetin from onion skin extract, for 6-weeks.	70 participants with overweight or obesity and pre-hypertension. Aged between 25 and 65 years and average BMI 31.1 kg/m^2^.	Double-blinded, randomized, placebo-controlled crossover trial.	No significant changes: - Leptin, adiponectin, HOMA-AD, ratio leptin/adiponectin and ratio adiponectin/leptin - CRP, TNFα - Plasma glucose, insulin, HOMA-IR, biomarkers of liver and renal function.	Relatively large sample size. Males and females were equitably represented. Plasma flavonol concentrations were analyzed, high compliance. No dietary habit assessment during intervention periods. High inflammation biomarkers variance.
Dower et al. (111)	160 mg/day quercetiω-3-glucoside, 100 mg/day (–)-epicatechin for 4-weeks.	37 healthy participants. Aged between 40 and 80 years and average BMI 26.7 kg/m^2^.	Double-blinded, randomized, placebo-controlled crossover trial.	(–)-epicatechin: ↓ insulin resistance No significant changes, both: - FMD - Plasma glucose, (insulin resistance quercetiω-3-glucoside), HOMA-IR, nitric oxide chronic and acute, TC, LDL-C, HDL-C, TG - Endothelin-1.	Pure quercetin-3-glucoside and (–)-epicatechin were used. Compliance and urine and plasma flavonoid concentrations were checked. 24-h BP was assessed. Small sample size and short intervention period. There were two major adverse events (during washout periods).
Pfeuffer et al. (112)	150 mg/day quercetin dihydrate for 8-weeks.	49 healthy men with different APOE genotypes: 3/3 (*n* = 19), 3/4 (*n* = 22) and 4/4 (*n* = 8).	Double-blinded, randomized, placebo-controlled crossover trial.	↓ waist circumference Fasting parameters: ↑HDL-C, TNF-α Postprandial parameters: ↓SBP, TG, ↑ HDL-C No significant changes: - BMI, body weight, endoPAT, fasting SBP, DBP, glucose, insulin, HOMA-IR, TG, TC, LDL-C - sE-selectin, sVCAM, sICAM, oxLDL, GSH, CPR, 8-iso-PGF2α.	Compliance was checked. Postprandial response analyzed. Showed genotype interaction effect. Small sample size. No dietary habit assignment. Baseline CRP group differences.
**OTHER FLAVONOIDS**					
Huang et al. (113)	Green tea extract: 856.8 mg EGCG /day, 236.1 mg ECG/day, 115.5 EGC/day, etc. For 6 weeks.	73 women with overweight or obesity and high LDL-C levels. Aged between 18 and 65 years and BMI ≥ 27 kg/m^2^.	Double-blinded, randomized, placebo-controlled crossover trial.	↑ leptin ↓ LDL-C No significant changes: - BMI, fasting blood sugar, total cholesterol, triglyceride, high density lipoprotein, adiponectin and ghrelin.	Precise description of extract characteristics./ Short intervention period, exclusion criteria did not specifically include cholesterol treatment drugs, supplementation intake or excessive dietary intake such as tea, coffee, and no dietary habit assessment.
Venkatakrishnan et al. (114)	Catechin-enriched green tea (780.6 mg of catechin) or catechin-enriched oolong tea (640.4 mg of catechin), daily. For 12 weeks.	60 mildly hypercholesterolemic subjects (180–220 mg/dL). Aged between 35 and 55 years.	Double-blinded, randomized, placebo-controlled trial.	Both teas: - ↓ weight, body fat and BMI, TC, LDL-C, TG - ↑ Trolox equivalent antioxidant capacity (TEAC), glutathione, ↓lipid peroxidation products - ↓oxLDL - ↑ SOD, CAT, and GPx activity No significant changes: - HDL-C, glutathione reductase, AST, ALT.	High compliance. Total phenolic blood levels measured during the study. Small sample size (3 groups). Limited information on beverage composition (placebo included). No dietary intake assessment.
Saarenhovi et al. (115)	Apple polyphenol extract: 100 mg epicatechin/day for 4-weeks.	60 participants with borderline hypertension (BP 130–139/85–89 mmHg) or unmedicated mild hypertension (BP 140–165/90–95 mmHg) Aged between 40 and 65 years, average BMI 25.5 kg/m^2^.	Double-blinded, randomized, placebo-controlled crossover trial.	No significant changes: - FMD, NMD, SBP, and DBP, plasma lipids (TC, LDL-C, HDL-C, TG), ALA, ASA, GGT, and other biochemical parameters. - sICAM-1, sVCAM-1, PAI-1, CRP, ADMA, vWf, and sE-selectin.	Precise description of extract characteristics. Dietary intake assessment. Predose epicatechin blood levels measure. Short intervention period. No ambulatory BP data. Sequence of administration had effect on FMD.
Samavat et al. (116, 117)	Green tea extract: containing 1,315 mg catechins (843 mg EGCG) for 12 months.	936 healthy postmenopausal women. Aged between 50 and 70 years, and BMI 18.5–40 kg/m^2^.	Double-blinded, randomized, placebo-controlled trial.	↓ TC, LDL-C ↑ TG No significant changes: - TC:HDL-C ratio, Non-HDL cholesterol - energy intake, body weight, BMI, or waist circumference - circulating leptin, ghrelin, adiponectin, or glucose concentrations.	Large sample size and relatively long intervention period, high compliance. Limited generalizability (predominantly non-Hispanic white and educated), long blood samples storage (1–3 y), significant differences in supplement intake at baseline.
Homayouni et al. (118, 119)	500 mg/day of hesperidin for 6-weeks.	64 participants with T2DM aged between 30–65 years and BMI < 30 kg/m^2^.	Double-blinded, randomized, placebo-controlled trial.	↓ SBP, mean BP ↑ total antioxidant capacity ↓ TNF-α, IL-6, hs-CRP ↓ froctosamine No significant changes: - DBP, fasting blood glucose, HOMA-IR, 8OHDG.	Dietary intake assessment and high participant compliance. Lost follow-up prevented intention-to-treat analysis.
Salden et al. (120)	450 mg/day of hesperidin 2S for 6-weeks.	68 participants with overweight or obesity, aged between 18 and 65 years and BMI 25–35 kg/m^2^.	Double-blinded, randomized, placebo-controlled trial.	No significant changes: - FMD, SBP, DBP, heart rate - sVCAM, sICAM, sE-selectin, sP-selectin - TC, LDL-C, HDL-C, TG - Glucose, insulin, QUICKI.	Relatively large sample size. Males and females were equitably represented. Plasma flavonol concentrations were analyzed, high compliance. No dietary habit assessment during intervention periods. High inflammation biomarkers variance.

*AA, arachidonic acid; Ach, acetyl choline; ADMA, asymmetric dimethylarginine; AGEs, advanced glycation endproducts; Aix, augmentation index; ALA, α-linolenic acid; AP, augmented pressure; ASCVD, atherosclerotic cardiovascular disease; BA, below average; BMI, body mass index; BP, blood pressure; CAD, coronary artery disease; CHD, coronary heart disease; CI, confidence interval; CLA, conjugated linoleic acid; CRP, C-reactive protein; CVD, Cardiovascular diseases; DGLA, dihomo-γ-linolenic acid; DHA, docosahexaenoic acid; DTM, digital thermal monitoring; EC: endothelial cells; EPA, eicosapentaenoic acid; FA, fatty acids; FMD, flow-mediated dilation; Foxp3, forkhead box protein-3; FRAP, ferric-reducing antioxidant power; GPx, glutathione peroxidase; HbA1c, glycated hemoglobin; HDL-C, high-density lipoprotein cholesterol; HF, Heart failure; HR, Hazard ratio; hs-CRP, high sensitivity C-reactive protein; IDLs, intermediate-density lipoprotein cholesterol; IFN-α, Interferon alpha; IFN-γ, Interferon gamma; IL-6, interleukin-6; IMT, intima-media thickness; KTR, kynurenine-to-tryptophan ratio; LDL-C, low-density lipoprotein cholesterol; LOOH, plasma lipoperoxides; Lp(a), lipoprotein a; LPDP, lipoprotein-depleted-plasma; LXA4, lipoxin A4; MDA, malondialdehyde; MCP-1, monocyte chemoattractant protein-1; MetS, metabolic syndrome; MI, myocardial infarction; MMP, metalloproteinases; NDM, nitroglycerin-mediated dilation; NEFA: non-esterified fatty acids; NF-κβ: nuclear transcription factor signaling; NMD: nitroglycerin-mediated dilation; NO, nitric oxide; NT-pro-BNP, N-terminal pro b-type natriuretic peptide; oxLDL, oxidized low-density lipoprotein; PAI-1, plasminogen activator inhibitor type 1; PBMCs, peripheral blood mononuclear cells; PGE1, prostaglandin E1; PWV, pulse wave velocity; RCT, randomized controlled trials; RORc, retinoid-related orphan receptor-c ; RR, relative risk; SAA, serum amyloid A; SAP, serum amyloid P component; sCD40L, soluble CD40 ligand; sICAM-1, soluble intercellular adhesion molecule 1; SMD, standardized mean difference; SNP, sodium nitroprusside; SOD, superoxide dismutase; sVCAM-1, soluble vascular cell adhesion molecule-1; T2DM, diabetes mellitus type 2;T-bet, T helper 1 cell lineage commitment; t-PA, tissue plasminogen activator; TC, Total cholesterol; TG, triglycerides; TGF-β, transforming growth factor-beta; Th17, T-helper 17;TNF-α, tumor necrosis factor-alpha; TXA2, thromboxane; TXB2, thromboxane B2; VEGF, vascular endothelial growth factor; VLDL, very low-density lipoprotein; WBC, white blood cell count; VSMC, vascular smooth muscle cells; WMD, weighted mean difference; vWf, Von Willebrand factor*.

On the other hand, there is a large amount of evidence showing that ω-3 FA can reduce the concentrations of several inflammatory markers related to atheroma development and plaque stability. Tousoulis et al. (35) performed a randomized, placebo-controlled, double-blind, cross-over study in 29 subjects with metabolic syndrome (MetS) in which the participants were supplemented with 2 g/ day of ω-3 FA for 12-weeks. The results showed a significant reduction in the plasma concentrations of IL-6 and a significant increase in PAI-1 levels after ω-3 FA treatment. A large number of studies have also reported an improvement in FMD as a measure of endothelial function after ω-3 FA supplementation (121–124). In contrast, in a population of 36 very high-risk participants with established atherosclerotic cardiovascular disease (ASCVD) and T2DM, Siniarski et al. (36) did not observe any significant changes in endothelial function indices (FMD and nitroglycerin-mediated dilation, NMD) after administering 2 g of ω-3 FA (1,000 mg of DHA + 1,000 mg of EPA) during 3 months. Cawood et al. (37) showed that a higher EPA content is associated with less inflammation, greater stability plaque and less T cell infiltration, as well as a smaller number of foam cells. Similar results were described by Thies et al. (38) in a randomized controlled trial including patients awaiting carotid endarterectomy. The participants were randomized to receive fish oil (ω-3), sunflower oil (ω-6) or placebo capsules during a median of 42 days before surgery. Those in the fish oil group showed higher plaque stability with the presence of thinner fibrous caps and fewer signs of inflammation, less lymphocyte infiltration, and greater inhibition of macrophages compared with the control and sunflower oil groups. In another study Nozue et al. (125) showed that progression of atherosclerosis was directly linked with an increase in the ω-6/ω-3 ratio. Thus, Zhao et al. (39) investigated the effect of ω-3 FA on circulating pro-inflammatory markers and NT-proBNP in volunteers with heart failure. They found that after 3 months with ω-3 FA treatment, plasma levels of TNF-α, IL-6, sICAM-1, and NT-proBNP significantly decreased in the participants allocated to the ω-3 FA intervention. Finally, Allaire et al. (40) compared, the effects of EPA vs. DHA supplementation on inflammatory markers and blood lipids in a population at high risk of CVD. They concluded that compared to EPA, DHA has a greater modulating effect, producing a larger reduction of CRP, IL-6, TNF-α, and TG levels, with a higher increase of adiponectin and high-density lipoprotein cholesterol (HDL-C) levels. In other double-blind trial (41), 111 healthy elderly subjects were randomly allocated to one of three dietary interventions: (1, 2) daily consumption of EPA+DHA at different doses (1.8 or 0.4 g), or (3) daily consumption of 4 g of high–oleic acid sunflower oil. A high consumption of EPA + DHA led to a change in the expression of 1,040 genes. In addition, the group receiving 1.8 g of EPA + DHA showed a significant reduction in the expression of peripheral blood mononuclear cells (PBMCs) genes involved in inflammatory- and atherogenic-related pathways, including eicosanoid synthesis, nuclear transcription factor signaling (NF-κβ), scavenger receptor activity, adipogenesis, and hypoxia signaling.

The heterogeneity of the results could be explained by various factors such as insufficient dose (< 1,000 mg/d), origin (lean fish, fish oil, fatty fish, etc.), the type of supplementation (EPA, ω-3 FA, DHA, EPA + DHA, etc.), whether ω-3 FA were given alone or in combination with other bioactive compounds, and thus, synergistic effects might explain some of effects observed. In addition, the target population (healthy, MetS, ASCVD, CAD, T2DM, etc.), sample size, the long follow-up period and high adherence to study supplementation differs among the trials. Therefore, ω-3 FA supplementation may be effective at an earlier stage of atherosclerosis disease, while in a very high-risk population with advanced atherosclerotic disease its effectiveness may be limited. Taking this into account, the additional benefits of ω-3 FA on endothelial function might have been reduced by optimal treatment such as concomitant cardioprotective therapies which the patients had already received.

## Omega-6

There is evidence suggesting that a higher intake of ω-6 fats, together with a lower intake of saturated fat may reduce the incidence of CHD. On the other hand, a large body of literature has suggested that a higher intake of ω-6 may promote inflammation and contribute to the pathogenesis of many diseases, including CVD, because AA promotes the synthesis of a variety of pro-inflammatory eicosanoids (126). Therefore, a reduction of tissue AA content (reducing linoleic intake) should lead to a lower risk of CHD reduction since the production of inflammatory molecules would also be reduced (127). However, since dihomo-γ-linolenic acid (DGLA) can be metabolized into prostaglandin E1 (PGE1), a potent anti-atherogenic compound, it confers anti-atherogenic properties to ω-6 FA (128).

To date, there is not enough evidence related to the harm or the benefit of ω-6 on CVD, and more concretely, on atherosclerosis. In a recent systematic review (129) on the effects of ω-6 FA on cardiovascular health, mortality, lipids, and adiposity (19 RCTs including 6,461 participants followed for 1–8 years) found no evidence of effects of dose-response or duration for any primary outcome (all-cause mortality, CVD mortality, CHD events, CHD events, stroke or major adverse cardiac, and cerebrovascular events). However, the authors observed that participants with lower ω-6 FA intake at baseline seemed to have greater protection, and an increased intake of ω-6 FA may reduce the risk of myocardial infarction (MI) (RR 0.88, 95%CI 0.76 to 1.02). In addition, a meta-analysis (130) of 11 RCTs including 420 subjects showed that conjugated linoleic acid (CLA) supplementation increased blood levels of CRP by 0.89 mg/L (95% CI: 0.11, 1.68; *P* = 0.025) and TNF-α levels by 0.39 pg/mL (95% CI: 0.23, 0.55; *P* < 0.0001). Nonetheless, another meta-analysis (131) concluded that CLA supplements had a proinflammatory effect after observing an increase in plasma CRP concentrations and significant reductions in serum adiponectin concentrations independently of the dosage of CLA supplementation (0.63 mg/dL, 95% CI: 0.13, 1.13, heterogeneity *P* = 0.026; *I*^2^ = 52.3%). In contrast, after analyzing 15 RCTs, Johnson et al. (132) concluded that there is insufficient evidence to show that a diet supplemented with linoleic acid increases the concentrations of pro-inflammatory markers [adiponectin, complement, CRP, E-selectins, fibrinogen, interleukins, lipoprotein-associated phospholipase A2, lipoxins, monocyte chemoattractant protein-1 (MCP-1), PAI-1, platelet-derived growth factor-A, serum amyloid A protein (SAA), soluble CD-40 ligand, soluble IL-6 receptors, ICAM-1, soluble TNF receptor-1, soluble TNF receptor-2, sVCAM-1, thromboxane A2 (TXA2), thromboxane B2 (TXB2), transforming growth factor-β (TFG-β), TNF-α, among others].

Although *ex-vivo* studies (133) have shown that ω-6 FA-enriched diets seem to be linked to the formation of oxidized low-density lipoproteins (oxLDL), there is growing evidence that ω-6 FA could exert an anti-inflammatory effect, reducing the development of atherosclerosis (128).

Interventional studies with AA supplementation (840 mg/d for 4 weeks) showed no effect on any metabolic parameter or platelet function (42). Neither have studies on supplementation with linoleic acid found any effect related to the reduction of atherosclerosis or cardiovascular risk factors (43). Sluijs et al. (43) performed a RCT in 401 overweight subjects who were randomly assigned to receive 4 g of cis-9, trans-11 (c9,t11) CLA or placebo supplements for 6 months. They reported that c9, t11 CLA supplementation did not produce any effect on BP, body composition, lipid or glucose metabolism, insulin resistance or CRP levels. However, Hassan Eftekhari et al. (44) found that a diet supplemented with both CLA and ω-3 FA could have a beneficial effect on inflammatory markers of high sensitivity C-reactive protein (hs-CRP) and oxidative stress [malondialdehyde (MDA), and glutathione peroxidase, (GPx)] in atherosclerotic patients.

Again, the heterogeneity of the RCTs, the relatively short duration of some of these studies, the great variability in the concentration of ALA supplementation, as well as limited statistical power because of the small number of subjects included and a considerable intra- and inter-individual variability among the inflammatory markers studied might not allow the detection of subtle changes. In addition to diet, several authors have reported that genetics might influence circulating/tissue AA (134, 135). Indeed, most African Americans carry a genetic variant of the FA desaturase gene that enhances the ability to convert LA to AA, which is associated with greater circulating CRP and a higher risk of CVD. Overall, these different studies highlight the need for further human trials evaluating the role of ω-6 FA in the prevention of CVD.

## Coenzyme Q_10_

Coenzyme Q (CoQ) or ubiquinone is an effective natural antioxidant that is produced *de novo* in animals. Many food sources such as meat, fish, nuts, and some oils are CoQ-enriched, but this antioxidant is most frequently found in dairy products, vegetables, fruits, and cereals (136). Ubiquinone plays a key role in the electron transport chain within the mitochondria (137). CoQ10 and the cholesterol biosynthesis pathway share intermediate products such as mevalonate, which is key in the synthesis of cholesterol. Individuals receiving statin treatment may present by a reduction in CoQ10 levels (126, 137). Deficiencies in CoQ10 have been associated with CVD, and therefore, CoQ10 supplementation may be an effective tool in the primary prevention of CVD (138, 139).

Taking into account the difficulty in establishing a usual safe upper level of intake (UL), several studies have used the observed safe level (OSL) risk assessment method and reported strong evidence of safety at intakes up to 1,200 mg/day. Nevertheless, higher levels of CoQ10 (3,000 mg/day) have been tested without adverse effects and may be safe (137).

Several meta-analyses and systematic reviews have reported the benefits of CoQ10 on health. In a meta-analysis including 15 studies involving 765 individuals, Zhang et al. (140) reported an improvement in glycemic control, and TG and HDL-C levels in patients with T2DM supplemented with CoQ10. Jorat et al. (141) observed a reduction in total-cholesterol (standardized mean difference (SMD) −1.07; 95% CI, −1.94, −0.21, *P* = 0.01) and an increase in HDL-C levels (SMD 1.30; 95% CI, 0.20, 2.41, *P* = 0.02) in patients receiving CoQ10 supplementation, while no changes were observed in LDL-C, lipoprotein a [Lp(a)] or TG levels. On the other hand, in a meta-analysis including 6 RCTs and 218 participants at high risk of CVD, Flowers et al. (142) only observed significant reductions in systolic BP but no improvement in other risk factors such as diastolic BP, total-cholesterol, LDL-C, HDL-C or TG. In addition, Gao et al. (143) reported that CoQ10 supplementation was associated with a significant improvement in endothelial function assessed by FMD (SMD 1.70, 95% CI: 1.00, 2.4, *P* < 0.0001). In another meta-analysis (144) including 17 RCTs and 412 subjects allocated to a CoQ10 group and 399 subjects to a control group, a diet supplemented with CoQ10 (60 to 500 mg/day for 1–4 weeks of intervention) led to a decrease in CRP levels [weighted mean difference (WMD): −0.35 mg/L, 95% CI: −0.64 to −0.05, *P* = 0.022), IL-6 (WMD: −1.61 pg/mL, 95% CI: −2.64 to −0.58, *P* = 0.002) and TNF-α (WMD: −0.49 pg/mL, 95% CI: −0.93 to −0.06, *P* = 0.027). Finally, the meta-analysis performed by Zhai et al. (145) also showed that CoQ10 supplementation may partly improve inflammatory status. They found that CoQ10 supplementation improved CoQ10 plasma levels by 1.17 μg/mL and decreased TNF-α levels (−0.45 pg/mL). However, no changes were observed for CRP or IL-6. Finally, in patients with CVD with baseline serum hs-CRP levels > 3 mg/L, these levels improved after receiving CoQ10 supplementation for more than 12 weeks (146).

On the other hand, several interventional studies have provided large scientific body evidence on the possible benefits of CoQ10 supplementation. On one hand, Mohseni et al. (45) performed a randomized double-blinded controlled clinical trial to investigate if CoQ10 supplementation can improve BP and serum lipoprotein concentrations in Iranian individuals with hyperlipidemia and MI after 12 weeks of intervention. The group receiving CoQ10-supplementation showed significant reductions of total-cholesterol, LDL-C and fibrinogen concentrations, as well as an increase in HDL-C concentrations (*P* < 0.001). A significant increase in plasma HDL-C (1.44 ± 0.18 vs. 1.14 ± 0.18 mmol/L) levels and systolic BP and diastolic BP was also observed in the two groups. More recently, Pérez-Sánchez et al. (46) reported that CoQ10 supplementation (200 mg/d for 1 month) improved endothelial function and mitochondrial activity in patients with antiphospholipid syndrome. In addition, Lee et al. (47) investigated the effects of CoQ10 supplementation on inflammatory markers such as hs-CRP, IL-6 and homocysteine and oxidative stress markers including MDA and superoxide dismutase (SOD) in 51 patients with CAD. The participants were randomized into three groups: (1) placebo or control group, (2) Q10–60 group, which received 60 mg/d of CoQ10, and (3) Q10–150 group which received 150 mg/d of CoQ10 for 12 weeks. Significant reductions of IL-6 (−14%, *P* = 0.03) were observed after the Q10–150 group intervention. Nevertheless, CoQ10 supplementation (200 mg/d) in 51 obese subjects with a body mass index (BMI) ≥ 25 kg/m^2^ did not significantly improve the lipid profile, arterial stiffness, oxidative or inflammatory markers as Lp(a), serum levels of oxLDL, white blood cell count or CRP after 12 weeks of intervention (48). In 65 intermediate risk firefighters, the FAITH randomized clinical trial (49, 50) evaluated the combined effect of CoQ10 with aged garlic extract (AGE) on pro-inflammatory markers and progression of atherosclerotic disease. The authors reported a significant reduction in serum CRP levels and an improvement in both endothelium function and pulse wave velocity after 1 year of intervention.

Although the results of several meta-analyses and intervention studies have suggested that CoQ10 may significantly reduce CRP, IL-6, and TNF-α levels and improve oxidative stress markers, lipid profiles and BP, these results should be interpreted with caution because of their heterogeneity, the short intervention period in some of them, the different doses for intervention, the small number of subjects enrolled in the RCTs and the limited number of studies performed. All these factors might contribute to the null effect observed by CoQ10 on proinflammatory biomarkers. Therefore, at present, the lack of consistent studies demonstrating the potential benefit of CoQ10 supplementation in the prevention of atherosclerosis, limit the use of CoQ10 as a nutraceutical. Nevertheless, there is sufficient scientific evidence demonstrating that statin therapy combined with CoQ10 supplementation might be useful to further reduce the atherosclerotic process.

## Vitamins

There is a large body of scientific evidence showing that vitamin intake may be beneficial in the prevention of cardiovascular events (147, 148). Among the possible mechanisms proposed, vitamins can reduce endothelial cell (EC) damage, modulate immune system response, retain vascular smooth muscle cell (VSMC) proliferation and migration, improve nitric oxide (NO) production, and inhibit oxLDL formation (147–150). In fact, vitamin A, C, E, and K deficiency are associated with a higher risk of CVD (151–156). It should be taken into account that vitamin A, C, and E supplementation has shown to be effective in the prevention of atherosclerosis in experimental animal models, but this remains to be demonstrated in clinical trials in humans. These studies were mainly performed in young/adult animal models based on early stages of atherosclerosis or *in vitro* studies, while clinical trials would involve older participants in advanced stage atherosclerosis (157). In addition, several studies have reported that low dietary consumption of antioxidant vitamins are linked to greater progression of atherosclerosis (158).

### Vitamin B Group

A large number of epidemiological studies have reported that high intake or circulatory concentrations of specific micronutrients such as vitamin B group (folate, vitamin B-6, and vitamin B-12, and homocysteine) may also be associated with reduced progression of carotid intima-media thickness (IMT) (158, 159).

To date, observational studies, RCTs and meta-analyses have failed to demonstrate that vitamin B supplementation can reduce cardiovascular risk factors or the morbidity and mortality associated with stroke, CHD and peripheral artery disease (160–163).

In the 2003–2004 NHANES study, consumption of vitamin B6 via diet or supplementation was inversely related to CRP levels after analyzing 2,686 eligible participants (164). Numerous interventional studies have investigated the role of vitamin B supplementation in the prevention of atherosclerosis. The results of the Women's Antioxidant and Folic Acid Cardiovascular Study (51) showed that the consumption of the combination of folic acid (2.5 mg), vitamin B6 (50 mg), vitamin B12 (1 mg) daily for 7.3 years led to a significant reduction of homocysteine concentrations without altering the concentrations of biomarkers of vascular inflammation (CRP, IL-6, ICAM-1, and fibrinogen). Peeters et al. (52) investigated the effects of 8 weeks of multivitamin supplementation (vitamin B6, B12, and folic acid) on plasma homocysteine concentrations and IL-6, IL-8, hs-CRP, and MCP-1. They only found a significant reduction in homocysteine concentration but not in the pro-inflammatory biomarkers. Similar results were found in another interventional study performed in 522 elderly patients with hyperhomocysteinemia, who were treated with vitamin B12 (500 μg) and folic acid (400 μg) or placebo daily for 2 years (53). In this case, the study failed to show improvement in endothelial function [sICAM-1, sVCAM-1, and vascular endothelial growth factor (VEGF)] or low-grade systemic inflammation (SAA and CRP) after the multivitamin treatment. On the other hand, supplementation with folic acid (0.8 mg/d) for 1 year led to a significant 28% reduction in homocysteine concentrations compared to the placebo group, but no changes were observed in the plasma concentrations of the inflammatory markers (54). In another study, patients with stable CAD were randomized into 3 groups: (A) folic acid plus vitamin B12 and B6, (B) folic acid plus vitamin B12, and (C) vitamin B6 alone, and it was found that vitamin B did not affect the levels of pro-inflammatory markers (soluble CD40 ligand, sCD40L, IL-6, CRP, and neopterin) related to atherosclerosis (55). Finally, according to the results of a study in which patients received pyridoxine treatment (40 mg) for 28 days, Ulvik et al. (56) suggested that pyridoxine preserved or increased the association between plasma vitamin B6 and inflammatory markers [CRP, white blood cell count (WBC), kynurenine-to-tryptophan ratio (KTR), and neopterin].

Although observational studies have shown a positive association between homocysteine concentrations and cardiovascular events, the findings of RCTs have currently shown no clear evidence of a protective effect of antioxidant B vitamin supplementation on the progression of atherosclerosis. The discordance among the different studies may be the result of different timing of B-vitamin supplementation according to the stage (early vs. advanced) of atherosclerosis. Nonetheless, the positive effect of vitamin B supplementation on the progression of atherosclerosis has only been studied in a few small and highly heterogeneous studies. Therefore, vitamin B supplementation should not as yet be used for the prevention of CVD until future research can demonstrate the real role of supplementation in the prevention of chronic disease.

### Vitamin A

Vitamin A is a fat-soluble vitamin, constituted by 3 active forms (retinoids): retinol, retinal, and retinoic acid, the most important being beta-carotene (β-carotene) because of its high antioxidant effect (165). The cardioprotective effects of carotenoids in humans have been related, among others, to an improvement in BP, glucose metabolism and the lipid profile, the harmful effects of smoking and every step of atherosclerotic progression including endothelial dysfunction, LDL oxidation, leukocyte, and smooth muscle cell activity (166).

However, to date, the results of many clinical trials on vitamin A supplementation against CVD are contradictory. In fact, several meta-analyses do not support the benefits of antioxidant vitamins such as vitamin A or β-carotene supplementation in the prevention of CVD (158, 167–171). One meta-analysis which analyzed different antioxidants such as vitamins A, C, E, or selenium as well as folate, vitamin B6 or vitamin B12 separately to evaluate the progression of atherosclerosis disease using B-mode ultrasound, intravascular ultrasound, or angiography, found no evidence of a protective effect of antioxidants or B vitamin supplements on atherosclerotic disease (158). Neither could another meta-analysis including 179 RCTs demonstrate any benefit of the intake of dietary supplements on CVD outcomes and all-cause mortality (171).

Few interventional studies have been performed on vitamin A supplementation. However, one interventional study including 31 atherosclerotic patients and 15 healthy controls (57) found that 4 months of vitamin A supplementation reduced the production of inflammatory cytokine IL-17 and the gene expression of the main transcriptor factor that controls T-helper 17 (Th17) cell differentiation, and retinoid-related orphan receptor-c (RORc). In another study, Sezavar et al. (59) evaluated the efficacy of vitamin A supplementation (25,000 IU of retinyl palmitate/day) in reducing the gene expression of interferon γ (IFN-γ) and T helper 1 cell lineage commitment (T-bet) in 16 atherosclerotic patients and 15 healthy controls who received supplemental of vitamin A daily for 4 months. They found that vitamin A supplementation was able to suppress Th1 cell activity in both the atherosclerotic and healthy participants. Finally, Mottaghi et al. (58) analyzed the role of vitamin A (25,000 IU retinyl palmitate per day, for 4 months) in forkhead box protein-3 (Foxp3) and TGF-β gene expression 31 atherosclerotic patients. They found a significant increase in the gene expression of TGF-β and concluded that vitamin A supplementation may delay the progression of atherosclerosis.

The apparent discrepancy between the results of observational and interventional studies may depend on several factors. Inadequate doses or treatment duration (usually short study periods) in addition, to the nature of the different populations studied (e.g., atherosclerotic or healthy participants), age or the sample size might explain the null findings. Studies on the administration of β -carotene in apparently healthy participants showed no evidence of benefits or harm in patients with CVD. However, the results of the administration of β -carotene to subjects with atherosclerosis or CAD suggest that β -carotene might provide significant benefits in CVD, because of a reduction of pro-inflammatory markers related to atherosclerosis disease. Nevertheless, depending on the concentrations, vitamin A can work as either an antioxidant or pro-oxidant [at a dose ≥ 25,000 IU/Kg of body weight (172)] and lead to cases of hypervitaminosis and even to intoxication, while supplementation with provitamin A, (i.e., β-carotene) has shown to be safer (173). Nonetheless, the results of some interventional studies seem to be encouraging and justify further long-term studies to assess the clinical effects of vitamin A supplementation in a larger cohort of patients.

### Vitamin C

The daily diet should include a high content of foods rich in vitamin C or ascorbic acid such as fruits (especially citrus fruits such as oranges or lemons) and vegetables such as green and red peppers, tomatoes, as well as broccoli or blackcurrants, among others. Cardiovascular risk can be reduced by vitamin C through different mechanisms such as inhibition of LDL oxidation, thereby reducing the development or progression of atherosclerosis. Additionally, vitamin C has been shown to reduce monocyte adhesion to the vascular endothelium (62, 174), which is an early step in the development of atheroma plaque. Furthermore, vitamin C is associated with an improvement in NO production, increasing vasodilation and lowering the BP (175, 176). Moreover, vitamin C seems to contribute to maintaining the stability of atheroma plaque (177, 178).

Many epidemiologic studies have investigated the role of vitamin C in CVD and have shown that increased vitamin C intake is linked to a lower prevalence of CHD (179–183) and cardiovascular risk factors (184, 185). Nevertheless, a recent meta-analysis suggested that vitamin C supplementation did not reduce major cardiovascular events [hazard ratio (HR) 0.99, 95% CI 0.89–1.10] (186). Neither have any major long-term clinical trials been able to demonstrate the positive benefits of vitamin C in heart disease (187–189) or related risk factors (61, 190). In relation to endothelial function, Ashor et al. (191) concluded that vitamin C supplementation improved endothelial function and this improvement was higher in individuals at higher cardiovascular risk such as those with atherosclerosis (SMD: 0.84, 95% CI: 0.41–1.26, *P* < 0.001), diabetics (SMD: 0.52, 95% CI: 0.21–0.82, *P* < 0.001) and patients with heart failure (HF) (SMD: 0.48, 95% CI: 0.08–0.88, *P* < 0.02).

In a 3-year observational study of 573 healthy individuals (50% women) from 40 to 60 years of age, Agarwal et al. (192) reported that contrary to vitamin C contained in natural food, vitamin C supplementation was linked with early accelerated progression of atherosclerosis measured by carotid IMT. Thus, subjects in the highest quartile showed a 3-fold higher progression than those in lowest quartile [20.3 ± 2.6 vs. 7.6 ± 1.8 μm/year (mean ± SD); *P* < 0.001]. Furthermore, carotid IMT progression increased according to the dose in individuals taking vitamin C supplements (*P*-trend = 0.0009). The consumption of dietary vitamin C and vitamin C supplementation was measured by different 24-h recalls.

Interventional studies have also shown mixed results. On one hand, the Antioxidant Supplementation in Atherosclerosis Prevention (ASAP) study (193) described a significant delay in the progression of atherosclerosis measured by a mean common carotid artery IMT of 74% (95% CI 36–89%, *P* = 0.003) in 520 hypercholesterolemic smoking and nonsmoking men after twice daily consumption of a combined supplementation of d-α-tocopherol (136 IU) and 250 mg of vitamin C during 3 years. These findings were later reproduced by Salonen et al. (60) who confirmed that combined supplementation of vitamin E and C delays atherosclerotic progression in hypercholesterolemic individuals. A RCT also reported significant improvement in serum levels of hs-CRP, IL-6, fasting blood glucose, and TG after 8 weeks of treatment with 500 mg vitamin C twice a day in hypertensive and/or diabetic obese patients (61). In addition, Woollard et al. (62) studied the effect of vitamin C supplementation on monocyte adhesion to ECs in healthy non-smokers. All individuals, with normal or below average (BA) plasma vitamin C concentrations at baseline received 250 mg of vitamin C daily during 6 weeks. The BA group showed greater monocyte adhesion to ECs (30%). After vitamin C supplementation, the BA group showed a great reduction in monocyte adhesion to ECs (−37%, *P* < 0.02), which were reduced to normal baseline levels. Despite numerous findings of the benefits of vitamin C supplementation, many other interventional studies have reported inconsistent results. The long-term results obtained by Bruunsgaard et al. (63) in the 3-year ASAP study did not show any anti-inflammatory effect in healthy men with slight hypercholesterolemia after combined daily intake of vitamin C (250 mg) and E (136 IU). After assessing different inflammatory markers, the authors did not observe any change in the circulating levels of TNF-α, IL-6, or CRP. In addition, Mullan et al. (64) found no short-term evidence (4 weeks) that consumption of a beverage with a high polyphenol content and supplementation with vitamin C provided any benefits in traditional or novel risk factors in overweight or obese subjects. Moreover, in a crossover study, Gutierrez et al. (65) did not find significant changes in the lipid profile, markers of oxidative stress (oxLDL, non-esterified fatty acids, NEFAs) inflammation (CRP, adiponectin, IL-6) or hypercoagulability (PAI-1 and fibrinogen) after treatment with different doses of vitamin C for 2-weeks. Finally, similar results were found in another interventional study performed by Dewell et al. (66) in which after 8-weeks of intervention with (1) usual diet with placebo; (2) usual diet and antioxidant supplements or (3) antioxidant-rich foods, there were no significant within-group changes or among-group differences in the inflammatory marker concentrations studied (IL-6, MCP-1, sICAM-1) (66).

Many studies (cohort and RCT) have suggested an inverse relationship between vitamin C intake and the risk of heart disease, while others have reported slight increases in the risk or have failed to show any effects. Although several studies have reported similar absorption of vitamin C supplementation and food sources, at present, the underlying mechanisms involved in the absorption of vitamin C from supplements remain unclear, and thus, more studies are needed. In addition, it should be noted that most of the evidence about the potential benefits of vitamin C supplementation is based on animal and observational studies. Nonetheless, continued investigation into the role of vitamin C in atherosclerosis progression and its relationship with anti- or- pro-inflammatory biomarkers related to disease is needed.

### Vitamin D

Despite encouraging results from observational studies, RCTs on vitamin D supplementation have shown mixed results (194–198). A meta-analysis of 51 trials by Elamin et al. (199) analyzed the possible benefits of vitamin D supplementation on CVD. Dietary vitamin D supplementation (400 IU/d−500,000 IU/year) did not improve glucose levels, the lipid profile or BP. Neither was greater protection against MI or stroke observed. On the other hand, it is known that vitamin D deficiency is associated with a pro-inflammatory profile (IL-1, IL-2, IL-6, or TNF-α) which is modulated by calcitriol (200). A recent meta-analysis of 20 RCTs including 1,270 participants (201) reported that vitamin D supplementation (200 IU/d to a single bolus dose of 300,000 IU) may reduce chronic low-grade inflammation in patients with T2DM. The data showed reduced levels of CRP (SMD −0.23; 95% CI, −0.37 to −0.09; *P* = 0.002) and TNF-α (SMD −0.49; 95%CI, −0.84 to −0.15; *P* = 0.005), as well as a diminished erythrocyte sedimentation rate (SMD −0.47; 95%CI, −0.89 to −0.05; *P* = 0.03). In addition, the group receiving vitamin D supplementation showed higher leptin concentrations (SMD: 0.42; 95% CI, 0.04–0.81; *P* = 0.03) compared with control group. More modest results were obtained in another meta-analysis (202) that included 17 RCTs and 1,012 patients with HF receiving daily doses ranging from 1,000 to 2,000 IU. In this case, the data analyzed only showed significant reductions of TNF-α concentrations (*P* = 0.04). No changes were observed in the concentrations of CRP, IL-6 or IL-10. Another meta-analysis including 13 RCTs and 1,955 obese and overweight participants suggested that there were no changes in the levels of inflammatory markers such as CRP, TNF-α, and IL-6 (203) after supplementation with vitamin D (700 IU/d to 200,000 IU/d). Finally, Beveridge et al. (204) reported that vitamin D supplementation (ranging from 900 to 5,000 IU; for was 4 weeks to 12 months) had no significant effect on the markers of vascular function studied [brachial artery FMD; reactive hyperemia index measured using finger plethysmography; pulse wave velocity (PWV) and pulse wave analysis; central aortic BP derived from peripheral artery tonometry; microvascular function measured using acetylcholine iontophoresis; and laser Doppler perfusion imaging] after 4 weeks of intervention.

Several observational studies have reported that lower levels of vitamin D are associated with pro-inflammatory status in healthy individuals (205–207) and those with inflammatory diseases such as T2DM, arteriosclerosis and inflammatory polyarthritis (208). Vitamin D levels are also inversely correlated with leptin (209, 210) and positively with adiponectin (210, 211).

Interventional studies have also reported mixed results. One study performed by Beilfuss et al. (67) investigated the possible relationship between vitamin D status and pro-inflammatory biomarkers (IL-6, TNF-α, and hs-CRP) in 332 overweight and obese individuals. The participants were randomized into one of three groups: (1) 40,000 IU vitamin D (cholecalciferol) per week; (2) 20,000 IU vitamin D per week, or (3) placebo. After 1 year of intervention, supplementation with vitamin D led to significant reductions of IL-6 levels and a significant increase of hs-CRP concentrations. In 118 diabetics with vitamin D deficiency, Tabesh et al. (68) examined the effect of vitamin D-calcium co-supplementation on pro-inflammatory markers (IL-6, TNF-α, hs-CRP) and adipocytokines (leptin and adiponectin). The participants were randomized in one of four intervention groups: (1) vitamin D + calcium placebo; (2) calcium + vitamin D placebo; (3) vitamin D + calcium; and (4) vitamin D placebo+ calcium placebo. The results showed significant reductions of leptin (−75, −56, and −92 ng/mL, respectively), TNF-α (−3.1, −3.1, −3.4 pg/mL) and IL-6 (−2, −4, −4 pg/mL, respectively) concentrations for calcium and vitamin D alone, and combined calcium-vitamin D supplementation (*P* < 0.05; all). Only the group receiving vitamin D-calcium supplementation showed a reduction in hs-CRP levels (−1.14 ± 0.25 vs. 0.02 ± 0.24 ng/mL, *P* = 0.09) compared to the control group. In another study, Schleithoff et al. (69) reported significant reductions of serum TNF-α concentrations as well as an increase in IL-10 concentrations after daily treatment with 2,000 IU in patients with HF. In an interventional study, Mousa et al. (70) found no effect of vitamin D supplementation on inflammatory markers (TNF-α, MCP-1, IFN-α and IFN- γ, and IL-1β, IL-6, IL-8, IL-10, IL-12, IL-17A, IL-18, IL-23, and IL-33) or *in vivo* NF-κβ activity in humans. Similar results were described by Waterhouse et al. (71) who found no significant changes in any of the cytokines (IL-6, IL-10, and CRP) or adipokines (leptin, adiponectin) studied, except for IL-6 which showed levels 2.8 pg/mL higher in the 1,500 μg group compared to the placebo group (75th percentiles: 11.0 vs. 8.2 pg/mL).

The biological or sociological differences between population subgroups might explain the effects observed, or lack thereof on proinflammatory biomarkers related to atherosclerosis disease. Several RCTs included a small sample (< 100 participants) and only a few described factors that might influence their results such as smoking status, season or sunlight exposure, physical activity or dietary vitamin D consumption. The type of vitamin D used (cholecalciferol or ergocalciferol) and the dosing protocols may introduce some confounding variables in the results reported. Furthermore, the absorption of vitamin D differs according to the ethnicity, age or healthy status of the individual. At least 4,000 IU of vitamin D daily, during 2–3 months, are required to obtain optimal levels of this vitamin (212). Vitamin D supplementation seems to improve inflammatory marker concentrations in subjects with chronic disease such as heart failure (213), systemic lupus erythematosus (214), inflammatory bowel disease (215), and chronic obstructive pulmonary disease (216). Nevertheless, the lack of a biological effect of vitamin D on these markers could be explained by the health status of the study population (higher or lower grade of inflammation). In addition, many RCTs have used low doses (700–2,000 IU daily), which could be insufficient to observe any positive effect on inflammatory markers. Although vitamin D supplementation could be an effective treatment to improve inflammation or atherosclerosis, further, well-designed large-scale, long-term studies are needed.

### Vitamin E

Although several animal studies have reported that vitamin E (α-tocopherol) supplementation is associated with an improvement in immune response in older animals following infection (217–219), previous interventional studies have yielded mixed results (75, 220, 221). Vitamin E is considered a potent antioxidant with anti-inflammatory properties against CVD. Supplemental vitamin E in animals models and human individuals exerts its benefits through several mechanisms that include a decrease in lipid peroxidation, and superoxide (O2-) production, as well as a reduction in the expression of scavenger receptors (SR-A and CD36), both of which are important in foam cell formation (222). High doses of vitamin E supplementation have been associated with a lower release of pro-inflammatory molecules such as IL-8, PAI-1, CRP, as well as a significant decrease in the adhesion of leukocytes to the endothelium (222).

Although many clinical trials in humans (223–225) have reported possible positive benefits of vitamin E intake in CVD, meta-analyses have not found any evidence of the atheroprotective effects of vitamin E (168, 226). Furthermore, some meta-analyses have suggested that high doses of vitamin E may increase all-cause mortality (227, 228).

A cross-sectional study examined association between the intake of vitamin E and other antioxidants such as vitamin C, carotenoids, Se, and Zn and hs-CRP levels in 2,924 participants from the region of Augsburg (Germany). Information regarding the intake of dietary supplements and medication in the last 7 days was collected in personal interviews. The authors reported that participants in the upper quartile (78 mg vitamin E/day) had 22% lower hs-CRP levels, when vitamin E was taken in combination with other antioxidants, compared with those without any vitamin E supplementation (229).

In a crossover study, Plantinga et al. (72) investigated the combined effect of vitamin C and E on endothelial function, arterial stiffness, and oxidative stress in 30 males with essential hypertension in the short term (8 weeks). After vitamin supplementation, FMD was significantly improved (*P* < 0.001) compared to placebo group, while arterial stiffness measured as central PWV was reduced (*P* < 0.01) and the augmentation index (AIx), measured as the ratio between augmented pressure (AP) and pulse pressure (PP), tended to decrease. In addition, serum vitamin concentrations and antioxidant capacity were significantly increased and levels of oxidative stress decreased. In a 4-year clinical study of 409 smokers, Magliano et al. (73) randomized the participants into one of two groups: those who received 500 IU per day of vitamin E or placebo. The results showed that vitamin E supplementation did not delay the advance of atherosclerotic disease measured by carotid IMT. However, vitamin E significantly reduced LDL oxidative susceptibility. Another RCT in 90 patients with CAD reported that high intake of α-tocopherol (1,200 IU of /d) for 2 years led to significant reductions of plasma biomarkers of inflammation and oxidative stress (74). Another study demonstrated the ability of tocopherols to reduce systemic oxidative stress, but not inflammatory markers such as hs-CRP, IL-6, TNF-α, or MCP-1 in patients with T2DM after a daily intake of 500 mg/day of α-tocopherol or mixed tocopherols rich in γ-tocopherol for 6 weeks (75). In addition, Gutiérrez et al. (76) attempted to clarify the effects of different doses of vitamin E [low-dose (200 IU/d), medium-dose (400 IU/d), and high-dose vitamins (800 IU/d)] combined with vitamin C for two weeks on the prevention of atherosclerosis in 11 diabetics. The primary outcomes studied were markers of oxidative stress including oxLDL and glutathione, inflammation (adiponectin and hs-CRP) and hypercoagulation (PAI-1 and fibrinogen). It was found that only low-dose vitamin intake reduced oxLDL production compared to the other study arms (*P* = 0.002).

It has been postulated that the mechanism by which vitamin E exerts its anti-inflammatory effects might be related to protein kinase C (PKC) dephosphorylation. *In vitro* studies have shown that the administration of RRR-α-tocopherol or d-α-tocopherol (natural) leads to a significant reduction of PKC activity and platelet aggregation compared to some types of rac-α-tocopherol (synthetic) (230). Some studies do not distinguish between the sources of the α-tocopherol, natural or synthetic, and this can induce important bias. The dose of vitamin E administered is also important. Previous studies have reported that supplementation with vitamin E at doses ≤ 400 IU/day does not lead to a decrease in inflammatory biomarkers (231). On the other hand, vitamin E doses between 600 and 1,200 IU/day can significantly reduce concentrations of IL-6 or TNF-α (232). It should be noted that doses of vitamin E > 400 IU/day are directly related to a significant increase in all-cause mortality (228).

In summary, studies should specify which isomers (α- or γ- tocopherol) are tested since different vitamin E isomers can have different biological effects on atherosclerosis. However, studies on isoforms other than α-tocopherol are limited. On the other hand, high doses of vitamin E might be linked to potential pro-oxidant effects and thus, consumption should be cautioned. Although α-tocopherol may have antiatherosclerotic effects in *in vitro* and animal studies, supplementation in humans continues to be controversial.

### Vitamin K

Vitamin K is a fat soluble which can be found in two natural forms: phylloquinone (vitamin K1) and menaquinones (collectively known as vitamin K2). Phylloquinone is mainly found in dark green leafy vegetables and vegetable oils (olive oil and soybean oil), while fermented dairy products such as cheese and fermented soy beans (natto) and animal products (chicken, butter, egg yolks) contain menaquinones. These two natural forms differ in side-chain length and degree of saturation. Vitamin K2 is the most biologically active form (233, 234). Vitamin K as well as vitamin D have been implicated in CVD and the activity of proinflammatory cytokines. Thus, several *in vitro* and animal studies have reported that vitamin K seems to suppress the production of these cytokines. However, the role of this vitamin in humans remains unclear (235, 236).

There is a large body scientific evidence showing that high intake of vitamin K2 is associated with a lower risk of CHD such as coronary vascular disease and vascular calcification (234, 237–242). The case-control Multi-Ethnic Study of Atherosclerosis (MESA) showed that lower serum vitamin K1 concentrations were associated with greater progression of coronary artery calcification (CAC) in participants receiving anti-hypertensive medication [OR (95% CI): 2.37 (1.38, 4.09)] (243).

A recent meta-analysis (244) evaluated the possible effects of vitamin K on cardiometabolic risk factors. The authors concluded that there was insufficient evidence about any beneficial effect of vitamin K supplements on cardiometabolic risk factors because vitamin K showed no significant effect on the lipid profile, BP, or glucose metabolism. Vitamin K supplementation only led to an improvement in CRP levels (*P* = 0.01) and the insulin sensitivity index (*P* < 0.001). Neither did Suksomboon et al. (245) (8 RCTs and 1,077 participants) find any effect of vitamin K supplementation on insulin sensitivity after observing no changes in the parameters analyzed such as insulin resistance, fasting plasma glucose, fasting plasma insulin, CRP, adiponectin, leptin, or IL-6 levels. Similar results were described in the meta-analysis by Shahdadian et al. (246) in which vitamin K supplementation had no significant effect on glycemic control in healthy subjects.

Very few intervention trials on vitamin K supplementation have been carried out. One intervention trial by Knapen et al. (77) investigated if menaquinone supplementation (180 μg/ day) had any effect on arterial stiffness in 120 healthy post-menopausal women in the long term (3-years). They authors reported a significant reduction in the beta stiffness index as a measure of mechanical arterial properties in the group receiving vitamin K compared to the placebo group. Nevertheless, no changes were observed in the concentrations of markers related to endothelial dysfunction [VCAM, E-selectin, and advanced glycation endproducts (AGEs)] or inflammation (hs-CRP, IL-6, and TNF-α). Kristensen et al. (78) did not observe any improvement in any of the risk markers analyzed (sICAM-1, sVCAM-1, PAI-1, fibrinogen, and plasma factor VII c). Finally, another interventional study evaluated the effect of vitamin K supplementation on CAC progression in 388 healthy older men and women. Two hundred individuals received multivitamin supplementation with 500 μg of phylloquinone, and the control group received a multivitamin alone daily for 3 years. Compared to the control group, the participants receiving phylloquinone supplements showed less CAC progression (−6%, *P* = 0.04) (79).

Animal and *in vitro* studies have reported the role of vitamin K in vascular calcification, while the evidence in humans is less clear. The discrepancies between the results obtained may be explained by the heterogenic populations studied. Indeed, the populations studied usually include postmenopausal women without established CVD and therefore, the lack of effect of vitamin K supplementation on carotid IMT might only be manifested in individuals with well-established atherosclerosis. Furthermore, in order to observe substantial changes on IMT longer intervention periods may be necessary. On the other hand, observational (241, 247), *in vitro* (248) and animals studies (249) have shown an inverse association between vitamin K status and inflammatory biomarker (IL-6 and CRP) concentrations. The inclusion of healthy individuals free of chronic diseases or elderly subjects at high cardiovascular risk may explain why inflammatory cytokine values remained unchanged. Specific studies are needed to obtain more in depth understanding of the use of vitamin K supplementation on atherosclerosis progression.

## Carotenoids

Carotenoids are a wide family of natural pigments that can be classified as carotenes (α-carotene, β-carotene, lycopene) or xanthophylls (lutein, fucoxanthin, canthaxanthin, zeaxanthin, β-criptoxanthin, capsorubin, and astaxanthin) depending on their chemical structure. Although there are more than 500 carotenoids, humans can only absorb 20 (250). The main dietary source of carotenoids are fruits and vegetables (251). These compounds have been related to positive effects on health mainly due to their antioxidant proprieties but also because of their role in intracellular communication and the immune system (252, 253). In addition, carotenoids are associated with a slowdown of atherosclerosis progression (250, 254).

Cheng et al. (255) analyzed 21 clinical trials and observed that supplementation with tomatoes, a carotenoid-rich food, was related to significant improvement in LDL-C levels [−0.22 mmol/L (95% CI −0.37, −0.06), a reduction in IL-6 (−0.25, 95% CI −0.49, −0.02) and a 2.53% increase in FMD. On analysis of lycopene carotenoid supplementation trials, they observed a relevant reduction in systolic BP (−5.66 mmHg: *P* < 0.002). Nevertheless, no relevant changes were found in other inflammation markers such as oxLDL, CRP, IL-6, or ICAM-1 (*P* > 0.05; all) (255).

On the other hand, a meta-analysis of observational studies concluded that higher dietary lutein intake was correlated with cardiovascular health, probably in relation to an effect on atherosclerosis and inflammatory markers (256). Another observational meta-analysis reported that circulating lycopene levels were inversely associated with the risk of stroke (RR: 0.693, 95% CI 0.503, 0.954) (257). These results coincide with those of Song et al. (258) RR: 0.83 (95% CI 0.69, 0.96) who also described a lower risk of CHD with lycopene intake (RR: 0.87; 95%CI 0.76, 0.98).

A recent interventional study conducted by Colmán-Martínez et al. (80) showed that supplementation with tomato juice, which is rich in lycopene, significantly reduced ICAM-1 and VCAM-1 levels (*P* < 0.001, both). These reductions were mainly associated with the presence of *trans*-lycopene (*r* = − 0.625 and *r* = −0.697; *P* < 0.001, respectively). By contrast, 8 weeks of supplementation with palm carotene was not associated with similar observations (81). ICAM-1 and VCAM-1 concentrations remained unaltered *(P* > 0.05, both) along with other physiological, circulatory and inflammatory markers of vascular function. In a longer clinical trial in renal transplant recipients receiving astaxanthin supplementation, Coombes et al. (82) did not observe changes in physiological markers of vascular function (PWV, FMD, and carotid artery IMT; *P* > 0.05, all). Nevertheless, Zou et al. (83) found a reduction in carotid artery IMT after a 12-month intervention with a lutein supplement (0.035 mm, *P* = 0.042) or lutein plus lycopene supplementation (0.073 mm; *P* < 0.001). Moreover, modifications in carotid artery IMT were negatively associated with serum lycopene levels, and therefore, this response seems to be more related to this carotenoid.

The lack of effectiveness of carotenoids on inflammatory biomarkers and the atherosclerostic process might be explained by their low bioavailability (~10–40%) and low plasma concentrations [~2 μmol/L (259)]. Furthermore, interindividual differences related to carotenoid absorption, degradation, metabolism, and excretion, in addition to the type of carotenoid studied (lutein, lycopene or β-carotene) as well as dose, and health status of the study population could partly explain the differences observed among the studies carried out. The scientific evidence currently available on the role of carotenoids in atherosclerosis remains unclear, making further randomized controlled clinical trials necessary.

## Phytosterols

Although there are few differences in the chemical structure of phytosterol, phytostanol, and cholesterol, these differences have a distinct functionality (260). The human organism is not able to synthetize these bioactive compounds, and therefore, they can only be incorporated from vegetal dietary sources (261). Composition analysis has shown that the largest amounts of these compounds can be found in vegetables oils, followed by tubers, legumes, and nuts and the lowest amounts are found in cereals, vegetables and fruits (262). However, nuts have the highest free phytosterol content (262), which are more bioavailable (263). The average daily phytosterol intake in the Western diet is estimated to be 296 mg (264), with the main plant sterols in the human diet being campesterol, β-sitosterol, and stigmasterol (265, 266).

Phytosterol intake is associated with a dose-dependent decrease in total cholesterol and LDL-C (267), and the consumption of 2 g of phytosterols per day is related to significant changes in cholesterol absorption and LDL-C plasma levels of 8–10% (267). However, results regarding the ability of phytosterols to diminish low-grade inflammation are controversial.

A meta-analysis of 20 RCTs including mainly overweight and obese adults from 44.5 to 66 years of age with hypercholesterolemia found that after an intake mean of 2.24 g/day (1.4–4 g/day) of phytosterol-rich foods, the absolute changes in plasma CRP concentrations were not significant (−0.10 mg/dL: 95% CI −0.26, 0.05). Neither were HDL-C plasma levels significantly modified (0.5 mg/dL −0.2; 1.2). However, plasma LDL-C and total-cholesterol levels were significantly reduced [−14.3 mg/dL; 95%CI −17.3; −11.3 and −16.4 mL;95% CI −20.1; −12.8, respectively (268)], coinciding with the results of previous meta-analyses (269–271). Plasma TG levels showed a significant decrease (−7.9 mg/dL: 95% CI −12.7; −3.1).

Although there are no further meta-analyses regarding phytosterol intake and cholesterol levels, several intervention studies have been carried out. In 32 overweight or obese subjects, Lambert et al. (84) investigated the effect of the intake of milk supplemented with phytosterols (1.6 g of plant sterols/250 mL of milk) vs. milk supplemented with ω-3 in a 4-week crossover trial. At a proteomic level, determination of the lipoprotein-depleted-plasma (LPDP) fraction showed a decrease of pro-inflammatory serum amyloid P component (SAP) levels. A significant reduction of MCP-1 gene expression (*P* = 0.026) was also observed after phytosterol-milk intake as well as a trend to an increase in interleukin 10 receptor (IL-10R) expression levels (*P* = 0.06) (84). These results suggest a relationship between phytosterols and activation of anti-inflammatory response. Another study including 18 healthy participants (85) undergoing a milk supplemented with plant sterols intervention (2.0 g free phytosterols) during 4 weeks found results following a similar trend. Hs-CRP serum levels significantly diminished after the intervention −0.32 mg/L (*P* < 0.05), and plasma lipoxin A4 (LXA_4_) concentrations increased (0.12 nmol/L, *P* < 0.05) as did nitrite and nitrate levels (*P* < 0.05, both). However, no relevant changes were observed in TNF-α plasma levels or markers of oxidative damage after a 4-week intervention with phytosterol-enriched milk (85). Daily phytosterol intake of 3.0 g of phytosterol-supplemented margarine during 18 weeks showed no changes in inflammatory biomarkers (CRP, SAA, IL-6, IL-8, TNF-α, and soluble intercellular adhesion molecule-1) compared to placebo in patients with hypercholesterolemia (88). The *z*-scores for low-grade inflammation (−0.04; CI 95% −0.16; 0.07) and endothelial dysfunction (−0.2, CI95% −0.15, 0.11) were not significant (88). Likewise, Heggen et al. (87) performed a study including two phytosterol-enriched margarines to evaluate endothelial marker function and inflammation. E-selectin serum levels reduced −8.5% (*P* = 0.012) with rapeseed-sterol margarine vs. controls. The other inflammatory markers analyzed (VCAM-1, TNF-α, total PAI-1, and activated PAI-1) showed no significant changes after the intervention (87).

At present, the data available on effects of the use of plant sterols alone or combined with statins to reduce cardiovascular risk is limited. On the other hand, while *in vitro* and experimental animal studies have reported anti-inflammatory effects derived from sterols, the current knowledge on the anti-inflammatory and anti-atherogenic effects of phytosterols/stanols derived from RCTs is scarce and inconsistent. It should be noted that when phytosterols are incorporated into high-fat spreads, their absorption produces higher reductions of cholesterol concentrations than those absorbed as free phytosterols (272). In addition, in order to avoid possible bias, it is important to consider the type of sterols administered (phytosterols or phytostanols), the study sample size, the ethnicity or health status of the individuals included in the study, follow-up duration, as well as the optimal dosage of phytosterol supplementation. Thus, although phytosterol supplementation has been consistently related to a reduction in blood lipid levels, especially total-cholesterol and LDL-C, there is currently insufficient evidence to identify any solid modulation in inflammation markers, making further studies necessary.

## Stilbenes

Stilbenes are a polyphenol group characterized by a 1,2-diphenylethylene nucleus (273), which can be obtained in the diet mainly from red wine, grapes, peanuts and berries (274). The anti-inflammatory and anti-oxidative effects of these compounds, especially resveratrol, have frequently been related to health benefits, including in atherosclerosis (275). Numerous *in vitro* and animal studies have been carried out with promising results, but these must be corroborated by clinical trials.

The results of one recently published meta-analysis show that high doses of resveratrol supplementation (≥150 mg/day) were associated with a significant reduction of systolic BP by −11.90 mmHg (95% CI −20.99, −2.81) (276). Similar results were found by Hausenbas et al. (277) and Harm et al. (278). The latter evaluated 9 intervention trials with resveratrol-enriched grape extract supplementation and found that systolic BP was reduced by−1.54 mmHg (*P* = 0.02), and the heart rate also diminished (−1.42 bpm, *P* = 0.01). Nevertheless, diastolic BP, blood lipid, and CRP levels were not modified (278), coinciding in part with the report by Sahebkar et al. (279). The results of the analysis of 10 RCTs showed that supplementation with resveratrol did not significantly modify plasma CRP levels [−0.144 mg/dL (95% CI −0.968, 0.680)], diastolic BP and systolic BP, or total-cholesterol, LDL-C, TG and glycemia, (*P* ≥ 0.05, all). Nonetheless, HDL-C showed a negative response with a significant reduction in these levels [−4.18 mg/dL; 95% CI−6.54; −1.82) (279)]. An large meta-analysis by Haghighatdoost and Hariri (280) studied the response of blood lipid levels to resveratrol supplementation. These authors analyzed 21 randomized clinical trials in which no significant reduction was observed in total cholesterol or LDL-C levels (−0.08 mmol/L; 95%CI: −0.23; 0.08 and −0.04 mmol/L; 95% CI: −0.21; 0.12, respectively), and HDL concentration were not modified (*P* = 0.269). Only TG showed a significant reduction after the intervention, but these were not robust (280).

Adipokine levels have also been related to atherosclerosis and cardiovascular risk, mainly in the leptin and adiponectin ratio (281). Several studies have also associated resveratrol with changes in these cytokines. In a recent meta-analysis of 9 RCTs, Mohammadi-Sartang et al. (282) observed that a high intake of a resveratrol supplement (≥100 mg/day) was associated with a significant increase of adiponectin levels [1.11 μg/mL (95% CI 0.88, 1.34)]. However, plasma leptin levels were not significantly modulated by resveratrol supplementation, independently of the dose (282).

In the last years, numerous RCTs have been carried out to study the effects of stilbene supplementation (mainly resveratrol). However, the supplementation doses and intervention periods ranged from 40 to 1,500 mg/day and from hours up to 3 months. Moreover, the responses observed varied among the different studies.

In a study including healthy adults, Macedo et al. (90) observed the effect of 100 mg *trans-*resveratrol supplementation daily over 3 months, but they found no significant changes in the metabolic parameters and inflammatory and oxidative markers analyzed vs. controls. Only GPx activity, a biomarker of oxidative stress, was significantly reduced compared with placebo (*P* < 0.05), but the meaning of this change was not clear. After a physical fitness test, GPx activity and TNF-α concentration were also reduced, while plasma glucose levels increased. The authors thereby concluded that the physical fitness test applied may have been insufficient to determine whether resveratrol had any relevant effect on the antioxidant systems of the participants. On the other hand, one small study (*N* = 9) with a higher resveratrol dose (1 g/day) and longer intervention period conducted by Espinoza et al. (91) found a significant, albeit small, reduction in TNF-α and MCP-1 (*P* < 0.05) after 4 weeks of intervention; however, these changes did not continue over time. Contrary to Macedo et al. (90) they found an increase in the total antioxidant capacity (91). Response to resveratrol supplementation has also been studied by Van der Made et al. (92, 93) in overweight and obese subjects (28.3 ± 3.2 kg/m^2^). As in healthy adults, no relevant significant metabolic changes were found in inflammatory and/or endothelial function markers after 4 weeks of 150 mg of *trans*-resveratrol supplementation and only diastolic BP and heart rate increased (*P* < 0.05). The results of subgroup analysis by gender or body mass index (≥ or < 30 kg/m^2^) did not differ (92, 93). Similar findings were obtained when Kitada et al. (94) used piceatannol (hydroxylated analog of resveratrol), instead of resveratrol, as a supplement. Only insulin sensitivity improved after the intervention in overweight men: plasma insulin levels were reduced by −18.8 ± 11.2% (*P* = 0.02) and HOMA-IR by −17.2 ± 11.5% (*P* = 0.02) (94). Neither have studies carried out in T2DM patients found changes in this regard (96–98). Bo et al. (96, 97) analyzed the effects of resveratrol (500 and 40 mg/day) in T2DM patients over 6 months, but failed to identify significant differences at a metabolic or inflammatory level. They did, however, observe that pentraxin 3, an acute phase protein related to the CRP in humans, increased 4.7–26.3% (*P* < 0.05) and the total antioxidant status also increased (28.5–44.8; *P* < 0.05). In addition, in participants receiving high doses of resveratrol supplementation total-cholesterol levels significantly increased (11.94 mg/dL; 95% CI 2.55; 21.33) (96, 97). This coincides with the results of Kjær et al. (95), who also observed an increase in total cholesterol, LDL-C and fructosamine levels in patients with MetS after supplementation with 1 g/day of resveratrol during 16 weeks. With respect to antioxidant capacity, the results of a study by Bo et al. (96) were in concordance with those of Seyyedebrahimi et al. (98) who observed an antioxidant effect in PBMCs and an increase in the expression of Nrf2 and SOD (*P* = 0.047 and *P* = 0.005, respectively) in patients with T2DM after resveratrol supplementation. These results also agree with those of Imamura et al. (99), who identified a reduction in oxidative stress and arterial stiffness (*P* < 0.01) in patients with T2DM supplemented with resveratrol during 12 weeks (99). At an inflammatory level, resveratrol supplementation (300–500 mg/day) showed a reduction in TNF-α vs. placebo (100, 102), but an intervention with 1.5 g/day did not show the same pattern in this inflammatory biomarker (101).

One reason for the lack of impact of resveratrol on inflammatory biomarkers may be the significant heterogeneity among the trials (size sample, type of sample, inflammatory status, dose of resveratrol, length of treatment, etc.), which can potentially lead to bias. A relatively small number of participants might not provide sufficient statistical power to estimate the effects of resveratrol on proinflammatory markers. In addition, plasma resveratrol levels which are too low might explain the lack of impact of resveratrol on atherosclerotic markers. Moreover, the different sources of resveratrol (*trans*-resveratrol or extracts containing resveratrol) with different compositions may be another limitation and may also induce bias. Therefore, larger studies and studies focusing on pro-inflammatory markers or improvement of BP or lipid profile are needed to evaluate the different anti-inflammatory effects of resveratrol in humans. Moreover, prospective studies including higher doses of resveratrol and longer duration of supplementation are necessary to determine the effect of resveratrol supplementation on biomarkers of inflammation and oxidative stress.

## Flavonoids

Flavonoids are a wide family of compounds characterized by a diphenylpropane skeleton (C_6_-C_3_-C_6_). These compounds are obtained from plant foods (283), and numerous studies have related flavonoids to healthy effects (284), and a reduction in the risk of mortality (285–287). However, the results of several meta-analyses have not clarified whether there is a linear dose-response relationship (285, 286). Regarding CVD, a meta-analysis of 4 prospective cohort studies by Grosso et al. (285), Kim and Je (286), Liu et al. (287), and Wang et al. (288) has shown that high flavonoid intake is associated with a reduction in cardiovascular mortality. In addition, a meta-analysis of other prospective studies found a significant reduction in the risk of mortality by CHD (287, 289), and a significant reduction in the risk of stroke (290). These evidences support the recommendation of plant-based diets. Future studies should be aimed at analyzing the main subgroups of flavonoids and evaluating the latest studies on flavonoid supplementation and its effect on health.

### Isoflavones

Isoflavones, an estrogen-like compound structurally similar to 17β-estradiol (104), are basically made up of daidzein, genistein, and glycitein. They are mainly found in soy, in which the most notable types of phyto-estrogen present are genistein and daidzein (291). Although the main source of isoflavones is soy bean, other products such as soy dairy substitutes, soy meat substitutes, soy paste and soy traditional foods are also a good source of isoflavones (291).

During the last years, many studies have reported that isoflavones, or one of their compounds, may have an important role in our health. In particular, studies have been aimed at determining whether isoflavones have a direct or indirect effect on protecting against atherosclerosis by improving the levels of some inflammatory molecules as well as improving body weight and the lipid profile. For example, the meta-analysis by Zhang et al. (292) studied the effects of soy isoflavone supplementation in non-Asian postmenopausal women. They found significant reductions in body weight (WMD: −0.515; 95% CI: −0.895 to −0.134; *P* = 0.008), glucose levels (WMD, −0.189; 95% CI: −0.344 to −0.033), and fasting insulin levels (WMD, −0.940; 95% CI: −1.721 to −0.159) with soy isoflavone supplementation. Thus, soy isoflavone supplementation could be beneficial for reducing body weight, and plasma glucose, and controlling insulin levels (293). However, the recent meta-analysis by Simental-Mendía et al. (294) did not find any significant alteration in circulating Lp(a) (SMD: 0.08, 95% CI: −0.05, 0.20, *P* = 0.228) plasma concentrations on investigating the impact of supplementation with soy isoflavones on plasma Lp(a) levels (294). This finding is in contrast with the findings of previous meta-analyses reporting that soy reduced total cholesterol and LDL-C and increased HDL-C; however, it must be highlighted that previous meta-analyses were not specifically performed on placebo-controlled trials that may have reduced their robustness (294). On the other hand, interventional studies have also investigated the relationship between soy supplementation and its benefits on human health. Sathyapalan et al. (103) recently evaluated the possible influence of soy isoflavone supplementation on cardiovascular risk markers. The study involved 200 women (mean age 55 y) with early menopause. At the end of the intervention, it was found that soy isoflavone supplementation significantly reduced metabolic parameters and systolic BP (*P* < 0.01), thereby significantly improving cardiovascular risk markers and calculated cardiovascular risk during early menopause compared to soy protein without isoflavones (103). Byun et al. (105) described the effect of Chungkookjang supplementation, a Korean fermented soybean food with approximately 50 mg/g of isoflavones, on body composition, dyslipemia, and risk factors for atherosclerosis in overweight/obese subjects. After the intervention, apolipoprotein A1 (Apo A1) was significantly increased in the male Chungkookjang group (*P* < 0.05) alone. In contrast, the women in Chungkookjang group showed a significant decrease in the percentage of body fat (PBF), and the lean body mass (LBM) was significantly increased (*P* < 0.05). Apo A1 was also significantly increased in both the placebo and the Chungkookjang group, whereas apolipoprotein B (Apo B) was significantly decreased in the Chungkookjang group (*P* < 0.05). In addition, in the Chungkookjang group, hs-CRP showed a tendency to decreasing and significantly differed between the two groups (*P* < 0.05) (105). These results suggest that supplementation with Chungkookjang may improve body composition and risk factors for CVD in overweight and obese adults. Additionally, in a similar study with Chungkookjang, Back et al. obtained results suggesting that with this fermented soybean food had potential anti-atherosclerotic effects that might be more pronounced when combined with a modification in lifestyle (106). Apart from the beneficial effects on the improvement of CRP concentrations (104, 291) and a reduction in subclinical atherosclerosis reported by Hodis et al. (104) isoflavones have also been described as an anti-inflammatory and immunomodulatory compound. Moreover, these authors reported an average reduction of 16% (*P* = 0.36) in carotid artery IMT progression in American postmenopausal women of 45–92 years of age who were given daily doses of 25 g soy protein containing 911 mg aglycon isoflavone equivalents or placebo for 2.7 years. On average, this group also showed a 68% lower carotid IMT progression rate than the placebo group (*P* = 0.05) (104). On the other hand, while prevention of the onset of the disease, known as primary prevention, is important for health, secondary prevention is also very valuable. Indeed, Chan et al. (107) investigated the effect of an oral isoflavone supplement on vascular endothelial function in patients with established CVD. They performed a randomized, double-blinded, placebo-controlled trial to determine the effects of isoflavone supplementation vs. placebo for 12 weeks on brachial FMD in patients with prior ischemic stroke. Isoflavone treatment resulted in a significant decrease in serum hs-CRP levels (treatment effect −1.7 mg/L, 95% CI −3.3 to −0.1, *P* = 0.033) and a significant increase of FMD (treatment effect 1.0%, 95% CI 0.1–2.0, *P* = 0.035). In addition, it was suggested that the vasoprotective effect of isoflavones was more pronounced in patients with more severe endothelial dysfunction. In conclusion, this study demonstrated that 12 weeks of isoflavone treatment reduced serum hs-CRP and improved brachial FMD in patients with clinically manifest atherosclerosis, thereby reversing their endothelial dysfunction status. These findings may have important implications for the use of isoflavones in secondary prevention in patients with CVD, in addition to conventional interventions (107).

It should also be highlighted that another important compound related to isoflavones is considered to have anti-atherogenic effects which seems to improve arterial stiffness and may also prevent CHD. This compound is S-equol, a metabolite that comes from the dietary soy isoflavone daidzein, and it has been suggested that the production of equol from daidzein by intestinal bacteria may produce the benefits obtained with isoflavones (103, 295). Nonetheless, the metabolism of daidzein differs depending on the study population. For example, in Western countries, only 30–50% of individuals are equol producers (103). Törmälä et al. (108) studied the effects of equol production and soy supplementation on vascular function in postmenopausal women under long-term use of tibolone. This synthetic steroid is an alternative treatment for postmenopausal symptoms, which induces a different estrogenic milieu than estrogen and may affect vascular health. What these authors found was that in postmenopausal tibolone users, the capacity to produce endogenous equol was associated with favorable vascular function. Thus, women who produce equol have better arterial compliance and endothelial function compared to women who do not produce equol (108).

Moreover, during the last years, many biomarkers associated with isoflavone intake have been identified by proteome analysis. Fuchs et al. (109) identified *in vivo* markers that responded to an 8-week dietary intervention with isoflavone-enriched soy extract in postmenopausal women who consumed 50 mg of isoflavones/day. After the intervention, the subjects showed a selected set of proteins responding to treatment that could be closely linked to the genesis and progression of atherosclerotic processes. The nature of the proteins identified suggests that soy isoflavones may increase anti-inflammatory response in blood mononuclear cells that might contribute to the atherosclerosis-preventive activities of a soy-rich diet. In addition, the changes observed in the marker proteins suggest that soy extract may protect the fibrinolytic system (109).

Several studies including animals, cell cultures, and clinical trials have addressed the anti-inflammatory properties of isoflavones. Nevertheless, the mechanisms by which isoflavones exert their potential anti-inflammatory effects still remain unclear. A large number of meta-analyses and interventional studies indicate that isoflavones or soy protein have no impact on plasma lipids or proinflammatory biomarkers. On one hand, it has been highlighted that most of these studies were not placebo-controlled trials, thereby reducing their robustness. In addition, the isoflavone content, the type of soy product used (soy protein), race, genetic background, environment, lifestyle, number of cases studied, and menopausal status are other confounding factors that might explain the discrepancies observed in the efficacy of isoflavones on the lipid profile or anti-inflammatory markers. Studies in postmenopausal women have reported a weaker effect of isoflavones because of the inability of healthy late postmenopausal women to produce equol, which is an active metabolite of the soy isoflavone with higher biological and pharmacological effects than isoflavones own (296). Equol is able to bind to estrogen receptors, lowering lipid concentrations, and reducing atherosclerosis (297). Therefore, although isoflavones may be used in a range of inflammatory diseases in addition to atherosclerosis, more extensive studies are still warranted to determine the underlying mechanisms and the potential adverse effects of isoflavone consumption (carcinogenic and immunosuppressive effects).

### Flavonols

Several groups have reviewed the scientific evidence available on total flavonol intake and the risk of mortality by CVD. In 2014 a meta-analysis of 13 prospective studies published by Wang et al. (288) observed a significant inverse relationship (RR = 0.89, 95% CI 0.84; 0.94), and dose-response analysis concluded that an increment of 10 mg of flavonol intake daily was associated with a 5% reduction in CVD risk (288). This agrees with the recently published meta-analysis by Grosso et al. (285) (RR = 0.87, 95% CI 0.76, 0.99) who also found a reduction in CVD risk with flavonol supplementation. These results, however, were not consistent with those of the meta-analysis by Kim et al. (286) who did not find any significant associations. On the other hand, a meta-analysis of 18 RCTs found relevant changes in cardiovascular biomarkers after flavonol supplementation: total-cholesterol, LDL-C and TG were reduced, HDL-C was increased, and fasting plasma glucose and blood pressure were also significantly reduced (*P* < 0.05, all). Moreover, these modifications seemed to be especially relevant in participants with blood lipid alterations and studies in Asian populations (298).

Quercetin is an ubiquitous dietary flavonol (299), which has been linked to numerous effects on health [antioxidant, antidiabetic, anti-obesity, anticarcinogenic, anti-atherosclerotic, antithrombotic, anti-allergic, and immune, inflammation, and cell signaling modulating activities (300)], thereby making it one of the most promising bioactive compounds for atherosclerosis therapy.

Meta-analyses of RCTs involving quercetin supplementation have shown a significant reduction of systolic and diastolic BP (300). Moreover, a reduction in circulating CRP levels of −0.33 m/L (95% CI −0.50, −0.15) was found in a meta-analysis of 7 RCT published by Mohammadi-Sartang et al. (301). These authors related significant effects to quercetin doses > 500 mg/day in subjects with normal levels of CRP (< 3 mg/L) (301). However, other meta-analyses did not observe any significant effects of quercetin supplementation on IL-6 or TNF-α concentrations (302) and plasma lipids (total-cholesterol, LDL-C, HDL-C, TG) (303).

In the last years, different RCTs have been carried out of quercetin supplementation and its possible effects on health. Brüll et al. (110) analyzed how supplementation with 162 mg of quercetin daily affects inflammatory biomarkers in patients with a high BMI and pre-hypertension, but they did not find any significant changes in CRP, TNFα, leptin or adiponectin levels. These authors also tested the acute effect 54 mg of quercetin supplementation on endothelial function and blood pressure after 4 h and again did not observe any significant changes in these values (304). Neither did Dower et al. (111) observe any significant changes in vascular function biomarkers, such as endothelin-1 and FMD. Pfeuffer et al. (112) investigated whether the effects of quercetin supplementation on atherosclerosis risk factors, inflammation biomarkers and oxidative stress depend on the apolipoprotein E (APOE) genotype. They found no association between the genotype and the effects of quercetin but did observe a significant reduction in waist circumference and an increase of HDL-C and TNF-α levels after supplementation compared to placebo, *P* < 0.05 (112). Another flavonol, dihydromyricetin, showed effects on glucose and lipid metabolism in patients with non-alcoholic fatty liver disease.

On one hand, flavonols might exert their cardioprotective effects by lowering BP, circulating LDL concentrations and reducing intracellular reactive oxidative species (ROS), as well as inhibiting the endothelial expression of adhesion molecules, the expression of which is related to the inhibition of NF-κβ and Activator protein 1 (AP-1) activation. The differences observed among the different studies may be attributed to the small number of participants and lack of effect of quercetin on endothelial function (antioxidant activity). All factors are key in the development of atherosclerosis. In addition, *in vitro* and animal studies have demonstrated the anti-inflammatory effects of quercetin at high plasma quercetin concentrations (>1 μM) (305), although some studies probably used quercetin concentrations which were insufficient to improve biomarkers of inflammation. Another limitation is the profile of the subjects studied. Although the study subjects were overweight-to-obese and had hypertension or MetS, they were metabolically healthy (excluding T2DM), limiting a further reduction of parameters such as glucose, hs-CRP, and hs-TNFα which were already low at baseline. Another possible limitation is the supplementation period (< 3 weeks), which may be insufficient to observe changes in markers of systemic inflammation and adiposity, both associated with inflammation. The collection of blood 8–12 h after quercentin intake may exert an acute effect at different sites of action and at a cellular level, might being able to twek and may have influenced its real effect on proinflammatory markers. Quercetin might not exert any effect on endothelial function because of a lack of antioxidant activity and oxidative stress. Finally, the different physiology of the species studied (humans and animals), as well as the different levels of inflammatory status might explain the different results obtained in the studies carried out. In addition, many RCTs use an enriched mixture of flavonols and a possible interaction with other phytochemicals and nutrients may explain the effects observed. Nonetheless, potential interactions with other phytochemicals and nutrients might be resolved using pure flavonols. Therefore, more RCTs are necessary to know the role of quercetin in atherosclerosis, and more specifically, its effects on inflammatory biomarkers.

### Other Flavonoids

The main dietary sources of flavan-3-ols (flavanols) are green tea, cocoa and berries. Flavan-3-ols have been associated with a reduction in the risk of all-cause mortality (285) and a lower mortality by CVD (285, 286, 288). A recently published Cochrane meta-analysis reported an association between flavan-3-ols from chocolate or cocoa products and a slight reduction in BP of 2 mmHg in healthy adults (-systolic BP: −1.76 mmHg, 95% CI: −3.09, −0.43 and diastolic BP: −1.76 mmHg, 95% CI: −2.57, −0.94). However, the authors highlighted the relevance of baseline BP, since pre-hypertensive participants seemed to present a higher response to cocoa flavan-3-ols than normotensive subjects (306). Another meta-analysis of 19 RCTs on cocoa flavan-3-ols found significant effects on inflammation and oxidative stress biomarkers: CRP (WMD: −0.83 mg/dL, 95% CI: −0.88, −0.77), VCAM-1 (WMD: 85.6 mg/mL, 95% CI: 16.0, 155), lipid metabolism (TG, HDL-C), and insulin resistance modulation (fasting insulin, HOMA-IR, QUICKI, quantitative insulin sensitivity check index, and the insulin sensitivity index, ISI) (307). A previous meta-analysis also found a modulation in HOMA-IR, and moreover, reported an improvement in FMD (1.43%; 95% CI: 1.00%, 1.68%) (308).

Catechins are the main flavan-3-ol present in green tea. A meta-analysis published by Khalesi et al. (309) found that green tea catechin intake was significantly associated with a reduction in BP (systolic BP −2.05 mmHg, 95% CI −3.06, −1.05 and diastolic BP −1.71 mmHg, 95% CI −2.86, −0.56) and plasma lipid modulation (total-cholesterol −0.15 mmol/L, 95 % CI −0.27, −0.02, LDL-C −0.16 mmol/L, 95 % CI −0.2, −0.09). Moreover, analysis by subgroups indicated that higher BP reductions were associated with green tea catechin intake < 500 mg/day.

On the other hand, a recent RCT published by Huang et al. (113) found that supplementation of 856.8 mg of epigallocatechin gallate (EGCG) to daily green tea extract intake over 6 weeks was associated with a significant increase of leptin levels of +25.7% (*P* < 0.048) and with decrease of LDL-C levels of 4.8% (113). Venkatakrishnan et al. (114) also observed significant reductions in LDL-C after 12 weeks of daily intake of catechin-enriched green tea or catechin-enriched oolong tea in mildly hypercholesterolemic subjects. Along with a reduction in total-cholesterol and TG, improvements were observed in antioxidant capacity [increased LDL oxidation lag time, SOD, GPx and catalase activity (CAT)] and oxidative indices (trolox equivalent antioxidant capacity, TEAC, glutathione, GSH and lipid peroxidation products reduction) as well as a significant reduction in weight, BMI and body fat (*P* < 0.05, all). In contrast, Saarenhovi et al. (115) did not observe significant changes in FMD, NMD, biochemical parameters (plasma fasting glucose and plasma lipids) or inflammatory biomarkers, adhesion molecules or coagulation markers [asymmetric dimethylarginine, ADMA, CRP, sE-selectin, von Willebrand factor (vWf), sICAM-1, sVCAM-1, PAI-1, CRP] after 4-weeks of supplementation with an apple polyphenol extract rich in epicatechin and flavan-3-ol oligomers. However, in a 1-year intervention RCT with green tea extract supplementation (including 843 mg of EGCG), Samavat et al. (116) observed that serum lipids were significantly modified in postmenopausal women: total-cholesterol decreased 2.1%, LDL-C 4.1% and non-HDL cholesterol 3.1% (*P* < 0.05, all). Nonetheless, HDL-C did not change after supplementation and TG concentrations increased (*P* = 0.046). Moreover, sub-analysis of the data found that the reduction in total cholesterol was especially relevant in women with high baseline total cholesterol levels (*P*-interaction = 0.01) (116), and fasting insulin concentrations also showed the same pattern, with the levels being significantly reduced in supplemented women with high baseline fasting glucose concentrations (117).

Flavanone intake has also been inversely related to a lower risk of all-cause mortality and to mortality by CVD (285, 288). One of the most relevant flavonones is hesperidin, an antioxidant compound that can be obtained from citrus fruit such as oranges or lemons. It has been related to effects over inflammatory biomarkers and blood pressure.

Recently, Homayouni et al. (118) observed that 500 mg/day of hesperidin supplementation in T2DM patients was related to anti-inflammatory effects in the short term (IL-6, TNF-α, hs-CRP reductions, *P* < 0.05) as well as a significant increase in the total antioxidant capacity in serum (13.4% ± 19.2) and a reduction in mean arterial BP of 2.5% ± 4.6. These authors also found a reduction in froctosamine (−10.10% ± 16.84), a constant biomarker of glucose level, and in hydroxydeoxyguanosine (8-OHDG) levels, a biomarker of DNA damage (*P* < 0.05, both). However, another similar study evaluating the effect of hesperidin supplementation (450 mg/day for 6-weeks) in volunteers with overweight or obesity found no significant improvement at an endothelial level. Only the adhesion molecules, VCAM-1 and sICAM-1, showed a tendency to diminish (*P* = 0.052 and *P* = 0.056, respectively). Moreover, no significant changes were observed in BP, plasma lipids, glucose parameters or FMD (120). However, it was observed that participants with FMD ≥ 3% showed better response to hesperidin supplementation with a reduction in VCAM-1 and sICAM-1 levels (*P* < 0.05) (120).

The lack of a significant effect of other flavonoids on atherosclerosis progression is unclear. Pharmacokinetic studies on different types of flavonoids are necessary to evaluate their possible acute biological effects and to obtain information on the best timing of FMD measurements after the administration of flavonoids. In addition, the discrepancies observed might be due to the different doses or composition of the flavonoids studied. New studies are needed to determine the most adequate dose, and studies on acute and long-term effects are also of interest. Other parameters such as age, sex, possible associated pathologies or grade of absorption of these flavonoids should also be considered in futures studies.

## Conclusions

The prevention of CVD is currently one of the greatest medical challenges at a global level. These diseases are associated with important morbidity and mortality, and thus, tools to aid in the prevention of CVD are key for the future. In this sense, there is growing evidence that a wide range of supplemental compounds have been related to the prevention of atherosclerosis or a slowing of further deterioration. Some of these compounds have been widely studied, such as vitamins, while others are new potential candidates which need to be investigated. Their mechanisms of action are diverse, producing effects at different levels, modulating inflammatory response, controlling oxidative stress, and stimulating or repressing key gene expression, among others. Nevertheless, to the date, several of these compounds lack scientific evidence to support their possible benefits in cardiovascular health (vitamin C, CoQ10, omega 6, stilbenes, flavonoids, among others).

Food supplements may be a good alternative for the prevention and treatment of atherosclerosis. Nevertheless, the lack of conclusive results about effectiveness of supplements on CVD, make more research in this field necessary.

One of the major challenges of immunonutrient supplementation is to identify the possible cardioprotective effects associated with the intake of a specific supplement with determined properties or in combination with other phytochemicals, or even in combination with other pharmaceutical therapies, in order to study the possible additional or synergistic benefits incurred and potential greater effectiveness. Therefore, robust, well-designed RCTs are needed to achieve greater evidence and to evaluate the effectiveness of supplementation and avoid bias, since the studies available have several limitations. Several strategies should be followed. On one hand, the study population should be well defined, focusing on the prevention of atherosclerosis and the participants should be individuals at high risk, albeit free, of CVD or should be diagnosed with previously established atherosclerosis in order to study secondary prevention. In both cases, the search for new biomarkers able to predict atherosclerosis linked to atherosclerosis regression or the use of new imaging techniques could be key in the design of these clinical trials. In addition, other parameters which should be controlled include the identification of more accurate oxidative biomarkers, and interindividual variation in the response to antioxidants (smoking, obesity, hypercholesterolemia, diabetes, elderly individuals, etc.) should be considered.

On the other hand, in many clinical trials the dose of the supplements studied is a clear limitation. The supplements administered often show no beneficial effect because the dose used is insufficient to observe any effect, and therefore, the dose administered should be physiologically relevant to humans (very high doses). In addition, it is essential that the composition and dose of the supplement studied as well as the length of supplementation, and interference or competition between phytochemicals be consistent to reduce the significant level of discrepancies among studies. More in depth knowledge of the absorption and bioavailability process, pharmacokinetic activity and the mechanisms underlying supplement absorption is required.

Further long-term RCTs are needed to fully evaluate the role of immunonutrient supplementation and its effect on anti-inflammatory response in atherosclerotic disease and determine the possible molecular mechanisms involved in the protective action of these supplements to develop new therapeutic approaches in the prevention of atherosclerosis.

## Author Contributions

RC and RE: conceptualization and methodology; RC, ML, AR-L: investigation and writing–original draft preparation; RC, AR-L, and RE: writing–review and editing; RC: visualization, supervision, project administration, and funding acquisition.

### Conflict of Interest Statement

RE reports serving on the board of and receiving lecture fees from the Research Foundation on Wine and Nutrition (FIVIN); serving on the boards of the Beer and Health Foundation and the European Foundation for Alcohol Research (ERAB); receiving lecture fees from Cerveceros de España and Sanofi-Aventis; and receiving grant support through his institution from Novartis. R. C. reports serving on the board of and receiving lecture fees from the Research Foundation on Wine and Nutrition (FIVIN). The remaining authors declare that the research was conducted in the absence of any commercial or financial relationships that could be construed as a potential conflict of interest.

## References

[1] Roth GA Forouzanfar MH Moran AE Barber R Nguyen G Feigin VL. Demographic and epidemiologic drivers of global cardiovascular mortality. N Engl J Med. (2015) 372:1333–41. 10.1056/NEJMoa1406656 25830423 PMC4482354

[2] Naghavi M Wang H Lozano R Davis A Liang X Zhou M GBD 2013 Mortality and causes of death collaborators. Global, regional, and national age-sex specific all-cause and cause-specific mortality for 240 causes of death, 1990-2013: a systematic analysis for the Global Burden of Disease Study 2013. Lancet. (2014) 385:117–71. 10.1016/S0140-6736(14)61682-2 25530442 PMC4340604

[3] Benjamin EJ Virani SS Callaway CW Chamberlain AM Chang AR Cheng S Heart disease and stroke statistics - 2018 update: A report from the American Heart Association. Circulation. (2018) 139:e56–e528. 10.1161/CIR.0000000000000659 30700139

[4] Lichtenstein AH Brands M Carnethon M Daniels S Franch HA Franklin B. Diet and lifestyle recommendations revision 2006 a scientific statement from the american heart association nutrition committee. Circulation. (2006) 114:82–96. 10.1161/CIRCULATIONAHA.106.176158 16785338

[5] Usda H 2015 Dietary Guidelines Advisory Committee Report. (2015). Available online at: https://health.gov/dietaryguidelines/2015-scientific-report/PDFs/Scientific-Report-of-the-2015-Dietary-Guidelines-Advisory-Committee.pdf (accessed November 28, 2018).

[6] Moore KJ Sheedy FJ Fisher EA Atherosclerosis results from a maladaptive inflamma. Nat Publ Gr. (2013) 13:709–21. 10.1038/nri3520 PMC4357520 23995626

[7] Tall AR Yvan-Charvet L. Cholesterol, inflammation and innate immunity. Nat Rev Immunol. (2015) 15:104–16. 10.1038/nri3793 25614320 PMC4669071

[8] Manson JE Cook NR Lee I-M Christen W Bassuk SS Mora S. Marine n−3 fatty acids and prevention of cardiovascular disease and cancer. N Engl J Med. (2018) 380:23–32. 10.1056/NEJMoa1811403 30415637 PMC6392053

[9] Erdman JW MacDonald I Zeisel SH International Life Sciences Institute Present Knowledge in Nutrition. International Life Sciences Institute (2012). Available online at: https://www.wiley.com/en-us/Present+Knowledge+in+Nutrition%2C+10th+Edition-p-9780470959176 (accessed November 27, 2018).

[10] Coates PM Encyclopedia of Dietary Supplements. Informa Healthcare (2010). Available online at: https://www.crcpress.com/Encyclopedia-of-Dietary-Supplements/Coates-Betz-Blackman-Cragg-Levine-Moss-White/p/book/9781439819289 (accessed November 27, 2018).

[11] Bowen KJ Harris WS Kris-Etherton PM. Omega-3 fatty acids and cardiovascular disease: are there benefits? Curr Treat Options Cardiovasc Med. (2016) 18:69. 10.1007/s11936-016-0487-1 27747477 PMC5067287

[12] Simopoulos AP. The importance of the omega-6/omega-3 fatty acid ratio in cardiovascular disease and other chronic diseases. Exp Biol Med. (2008) 233:674–88. 10.3181/0711-MR-311 18408140

[13] Balk E Chung M Lichtenstein A Chew P Kupelnick B Lawrence A. Effects of omega-3 fatty acids on cardiovascular risk factors and intermediate markers of cardiovascular disease. Evid Rep Technol Assess (Summ). (2004) 1–6. 15133887 PMC4781039

[14] Group TR PSC n−3 Fatty acids in patients with multiple cardiovascular risk factors. N Engl J Med. (2013) 368:1800–8. 10.1056/NEJMoa1205409 23656645

[15] Myung S-K Myung S-K Lee YJ Seo HG Group for the KMS. efficacy of omega-3 fatty acid supplements (Eicosapentaenoic Acid and Docosahexaenoic Acid) in the secondary prevention of cardiovascular disease. Arch Intern Med. (2012) 172:686. 10.1001/archinternmed.2012.262 22493407

[16] Oikawa S Yokoyama M Origasa H Matsuzaki M Matsuzawa Y Saito Y. Suppressive effect of EPA on the incidence of coronary events in hypercholesterolemia with impaired glucose metabolism: sub-analysis of the Japan EPA Lipid Intervention Study (JELIS). Atherosclerosis. (2009) 206:535–9. 10.1016/j.atherosclerosis.2009.03.029 19447387

[17] ORIGIN Trial Investigators Bosch J Gerstein HC Dagenais GR Díaz R Dyal L. n−3 Fatty acids and cardiovascular outcomes in patients with dysglycemia. N Engl J Med. (2012) 367:309–18. 10.1056/NEJMoa1203859 22686415

[18] Tenenbaum A Fisman EZ. Omega-3 polyunsaturated fatty acids supplementation in patients with diabetes and cardiovascular disease risk: does dose really matter? Cardiovasc Diabetol. (2018) 17:119. 10.1186/s12933-018-0766-0 30153832 PMC6112138

[19] Su K-P Tseng P-T Lin P-Y Okubo R Chen T-Y Chen Y-W. Association of use of omega-3 polyunsaturated fatty acids with changes in severity of anxiety symptoms. JAMA Netw Open. (2018) 1:e182327. 10.1001/jamanetworkopen.2018.2327 30646157 PMC6324500

[20] Rizos EC Ntzani EE Bika E Kostapanos MS Elisaf MS. Association between omega-3 fatty acid supplementation and risk of major cardiovascular disease events. JAMA. (2012) 308:1024. 10.1001/2012.jama.11374 22968891

[21] Hidayat K Yang J Zhang Z Chen G-C Qin L-Q Eggersdorfer M. Effect of omega-3 long-chain polyunsaturated fatty acid supplementation on heart rate: a meta-analysis of randomized controlled trials. Eur J Clin Nutr. (2018) 72:805–17. 10.1038/s41430-017-0052-3 29284786 PMC5988646

[22] Burke MF Burke FM Soffer DE. Review of cardiometabolic effects of prescription omega-3 fatty acids. Curr Atheroscler Rep. (2017) 19:60. 10.1007/s11883-017-0700-z 29116404

[23] Hamer M Steptoe A. Influence of specific nutrients on progression of atherosclerosis, vascular function, haemostasis and inflammation in coronary heart disease patients: a systematic review. Br J Nutr. (2006) 95:849–59. 10.1079/BJN20061741 16611374

[24] Massaro M Scoditti E Carluccio MA De Caterina R. Nutraceuticals and prevention of atherosclerosis: focus on ω-3 polyunsaturated fatty acids and mediterranean diet polyphenols. Cardiovasc Ther. (2010) 28:e13–9. 10.1111/j.1755-5922.2010.00211.x 20633019

[25] Calder PC. The role of marine omega-3 (*n*-3) fatty acids in inflammatory processes, atherosclerosis and plaque stability. Mol Nutr Food Res. (2012) 56:1073–80. 10.1002/mnfr.201100710 22760980

[26] Innes J Calder P. The differential effects of eicosapentaenoic acid and docosahexaenoic acid on cardiometabolic risk factors: a systematic review. Int J Mol Sci. (2018) 19:532. 10.3390/ijms19020532 29425187 PMC5855754

[27] O'Mahoney LL Matu J Price OJ Birch KM Ajjan RA Farrar D. Omega-3 polyunsaturated fatty acids favourably modulate cardiometabolic biomarkers in type 2 diabetes: a meta-analysis and meta-regression of randomized controlled trials. Cardiovasc Diabetol. (2018) 17:98. 10.1186/s12933-018-0740-x 29981570 PMC6035402

[28] Wang Q Liang X Wang L Lu X Huang J Cao J. Effect of omega-3 fatty acids supplementation on endothelial function: a meta-analysis of randomized controlled trials. Atherosclerosis. (2012) 221:536–43. 10.1016/j.atherosclerosis.2012.01.006 22317966

[29] Rangel-Huerta OD Aguilera CM Mesa MD Gil A. Omega-3 long-chain polyunsaturated fatty acids supplementation on inflammatory biomakers: a systematic review of randomised clinical trials. Br J Nutr. (2012) 107:S159–70. 10.1017/S0007114512001559 22591890

[30] Yang Y Lu N Chen D Meng L Zheng Y Hui R. Effects of n-3 PUFA supplementation on plasma soluble adhesion molecules: a meta-analysis of randomized controlled trials. Am J Clin Nutr. (2012) 95:972–80. 10.3945/ajcn.111.025924 22378734

[31] Franzese CJ Bliden KP Gesheff MG Pandya S Guyer KE Singla A Relation of fish oil supplementation to markers of atherothrombotic risk in patients with cardiovascular disease not receiving lipid-lowering therapy. Am J Cardiol. (2015) 115:1204–11. 10.1016/j.amjcard.2015.02.002 25759102

[32] Paulo MC Andrade AM Andrade ML Morais MG Kiely M Parra D. Influence of n-3 polyunsaturated fatty acids on soluble cellular adhesion molecules as biomarkers of cardiovascular risk in young healthy subjects. Nutr Metab Cardiovasc Dis. (2008) 18:664–70. 10.1016/j.numecd.2007.11.007 18420395

[33] Eschen O Christensen JH De Caterina R Schmidt EB. Soluble adhesion molecules in healthy subjects: a dose-response study using n-3 fatty acids. Nutr Metab Cardiovasc Dis. (2004) 14:180–5. 10.1016/S0939-4753(04)80002-4 15553594

[34] Yusof HM Miles EA Calder P. Influence of very long-chain n-3 fatty acids on plasma markers of inflammation in middle-aged men. Prostaglandins, Leukot Essent Fat Acids. (2008) 78:219–28. 10.1016/j.plefa.2008.02.002 18403189

[35] Tousoulis D Plastiras A Siasos G Oikonomou E Verveniotis A Kokkou E. Omega-3 PUFAs improved endothelial function and arterial stiffness with a parallel antiinflammatory effect in adults with metabolic syndrome. Atherosclerosis. (2014) 232:10–6. 10.1016/j.atherosclerosis.2013.10.014 24401211

[36] Siniarski A Haberka M Mostowik M Gołebiowska-Wiatrak R Poreba M Malinowski KP. Treatment with omega-3 polyunsaturated fatty acids does not improve endothelial function in patients with type 2 diabetes and very high cardiovascular risk: a randomized, double-blind, placebo-controlled study (Omega-FMD). Atherosclerosis. (2018) 271:148–55. 10.1016/j.atherosclerosis.2018.02.030 29518747

[37] Cawood AL Ding R Napper FL Young RH Williams JA Ward MJA. Eicosapentaenoic acid (EPA) from highly concentrated n−3 fatty acid ethyl esters is incorporated into advanced atherosclerotic plaques and higher plaque EPA is associated with decreased plaque inflammation and increased stability. Atherosclerosis. (2010) 212:252–9. 10.1016/j.atherosclerosis.2010.05.022 20542512

[38] Thies F Garry JM Yaqoob P Rerkasem K Williams J Shearman CP. Association of n-3 polyunsaturated fatty acids with stability of atherosclerotic plaques: a randomised controlled trial. Lancet. (2003) 361:477–85. 10.1016/S0140-6736(03)12468-3 12583947

[39] Zhao Y Shao L Teng L Hu B Luo Y Yu X. Effects of *n*−3 polyunsaturated fatty acid therapy on plasma inflammatory markers and N-terminal pro-brain natriuretic peptide in elderly patients with chronic heart failure. J Int Med Res. (2009) 37:1831–41. 10.1177/147323000903700619 20146881

[40] Allaire J Couture P Leclerc M Charest A Marin J Lépine M-C. A randomized, crossover, head-to-head comparison of eicosapentaenoic acid and docosahexaenoic acid supplementation to reduce inflammation markers in men and women: the Comparing EPA to DHA (ComparED) Study. Am J Clin Nutr. (2016) 104:280–7. 10.3945/ajcn.116.131896 27281302

[41] Bouwens M van de Rest O Dellschaft N Bromhaar MG de Groot LC Geleijnse JM. Fish-oil supplementation induces antiinflammatory gene expression profiles in human blood mononuclear cells. Am J Clin Nutr. (2009) 90:415–24. 10.3945/ajcn.2009.27680 19515734

[42] Kusumoto A Ishikura Y Kawashima H Kiso Y Takai S Miyazaki M. Effects of arachidonate-enriched triacylglycerol supplementation on serum fatty acids and platelet aggregation in healthy male subjects with a fish diet. Br J Nutr. (2007) 98:626. 10.1017/S0007114507734566 17445350

[43] Sluijs I Plantinga Y de Roos B Mennen LI Bots ML. Dietary supplementation with cis-9,trans-11 conjugated linoleic acid and aortic stiffness in overweight and obese adults. Am J Clin Nutr. (2010) 91:175–83. 10.3945/ajcn.2009.28192 19923377

[44] Hassan Eftekhari M Aliasghari F Babaei-Beigi MA Hasanzadeh J. Effect of conjugated linoleic acid and omega-3 fatty acid supplementation on inflammatory and oxidative stress markers in atherosclerotic patients. ARYA Atheroscler. (2013) 9:311–8. 10.4103/2277-9175.124644 24575132 PMC3933057

[45] Mohseni M Vafa MR Hajimiresmail SJ Zarrati M Rahimi Forushani A Bitarafan V. Effects of coenzyme q10 supplementation on serum lipoproteins, plasma fibrinogen, and blood pressure in patients with hyperlipidemia and myocardial infarction. Iran Red Crescent Med J. (2014) 16:e16433. 10.5812/ircmj.16433 25763201 PMC4329748

[46] Pérez-Sánchez C Aguirre MÁ Ruiz-Limón P Ábalos-Aguilera MC Jiménez-Gómez Y Arias-de la Rosa I. Ubiquinol effects on antiphospholipid syndrome prothrombotic profile: a randomized, placebo-controlled trial. Arterioscler Thromb Vasc Biol. (2017) 37:1923–32. 10.1161/ATVBAHA.117.309225 28684614

[47] Lee B-J Huang Y-C Chen S-J Lin P-T. Effects of coenzyme Q10 supplementation on inflammatory markers (high-sensitivity C-reactive protein, interleukin-6, and homocysteine) in patients with coronary artery disease. Nutrition. (2012) 28:767–72. 10.1016/j.nut.2011.11.008 22342390

[48] Lee Y-J Cho W-J Kim J-K Lee D-C. Effects of coenzyme Q10 on arterial stiffness, metabolic parameters, and fatigue in obese subjects: a double-blind randomized controlled study. J Med Food. (2011) 14:386–90. 10.1089/jmf.2010.1202 21370966

[49] Larijani VN Ahmadi N Zeb I Khan F Flores F Budoff M. Beneficial effects of aged garlic extract and coenzyme Q10 on vascular elasticity and endothelial function: the FAITH randomized clinical trial. Nutrition. (2013) 29:71–5. 10.1016/j.nut.2012.03.016 22858191 PMC4277702

[50] Zeb I Ahmadi N Nasir K Kadakia J Larijani VN Flores F. Aged garlic extract and coenzyme Q10 have favorable effect on inflammatory markers and coronary atherosclerosis progression: a randomized clinical trial. J Cardiovasc Dis Res. (2012) 3:185–90. 10.4103/0975-3583.98883 22923934 PMC3425023

[51] Christen WG Cook NR Van Denburgh M Zaharris E Albert CM Manson JE. Effect of combined treatment with folic acid, vitamin B6, and vitamin B12 on plasma biomarkers of inflammation and endothelial dysfunction in women. J Am Heart Assoc. (2018) 7:e008517. 10.1161/JAHA.117.008517 29776960 PMC6015379

[52] Peeters ACTM van Aken BE Blom HJ Reitsma PH den Heijer M. The effect of homocysteine reduction by B-vitamin supplementation on inflammatory markers. Clin Chem Lab Med. (2007) 45:54–8. 10.1515/CCLM.2007.021 17243915

[53] Van Dijk SC Enneman AW Swart KM Van Wijngaarden JP Ham AC De Jonge R. Effect of vitamin B12 and folic acid supplementation on biomarkers of endothelial function and inflammation among elderly individuals with hyperhomocysteinemia. Vasc Med. (2016) 21:91–8. 10.1177/1358863X15622281 26774115

[54] Durga J van Tits LJH Schouten EG Kok FJ Verhoef P. Effect of lowering of homocysteine levels on inflammatory markers. Arch Intern Med. (2005) 165:1388. 10.1001/archinte.165.12.1388 15983288

[55] Bleie Ø Semb AG Grundt H Nordrehaug JE Vollset SE Ueland PM Homocysteine-lowering therapy does not affect inflammatory markers of atherosclerosis in patients with stable coronary artery disease. J Intern Med. (2007) 262:244–53. 10.1111/j.1365-2796.2007.01810.x 17645592

[56] Ulvik A Midttun Ø Ringdal Pedersen E Nygård O Ueland PM. Association of plasma B-6 vitamers with systemic markers of inflammation before and after pyridoxine treatment in patients with stable angina pectoris. Am J Clin Nutr. (2012) 95:1072–8. 10.3945/ajcn.111.029751 22492365

[57] Mottaghi A Ebrahimof S Angoorani P Saboor-Yaraghi A-A. Vitamin A supplementation reduces IL-17 and RORc gene expression in atherosclerotic patients. Scand J Immunol. (2014) 80:151–7. 10.1111/sji.12190 24845870

[58] Mottaghi A Salehi E Keshvarz A Sezavar H Saboor-Yaraghi A-A The influence of vitamin A supplementation on Foxp3 and TGF-γ gene expression in atherosclerotic patients. J Nutrigenet Nutrigenomics. (2012) 5:314–26. 10.1159/000341916 23363776

[59] Sezavar H Saboor-Yaraghi A-A Salehi E Mottaghi A Whether vitamin A supplementation is effective in T-bet and IFN–γ gene expression reduction? Immunol Invest. (2015) 44:189–98. 10.3109/08820139.2014.953635 25496023

[60] Salonen RM Nyyssönen K Kaikkonen J Porkkala-Sarataho E Voutilainen S Rissanen TH. Six-year effect of combined vitamin C and E supplementation on atherosclerotic progression: the antioxidant supplementation in atherosclerosis prevention (ASAP) Study. Circulation. (2003) 107:947–53. 10.1161/01.CIR.0000050626.25057.51 12600905

[61] Ellulu MS Rahmat A Patimah I Khaza'ai H Abed Y Effect of vitamin C on inflammation and metabolic markers in hypertensive and/or diabetic obese adults: a randomized controlled trial. Drug Des Devel Ther. (2015) 9:3405–12. 10.2147/DDDT.S83144 PMC4492638 26170625

[62] Woollard KJ Loryman CJ Meredith E Bevan R Shaw JA Lunec J. Effects of oral vitamin C on monocyte: endothelial cell adhesion in healthy subjects. Biochem Biophys Res Commun. (2002) 294:1161–8. 10.1016/S0006-291X(02)00603-4 12074599

[63] Bruunsgaard H Poulsen HE Pedersen BK Nyyssönen K Kaikkonen J Salonen JT. Long-term combined supplementations with α-tocopherol and vitamin C have no detectable anti-inflammatory effects in healthy men. J Nutr. (2003) 133:1170–3. 10.1093/jn/133.4.1170 12672938

[64] Mullan A Delles C Ferrell W Mullen W Edwards CA McColl JH. Effects of a beverage rich in (poly)phenols on established and novel risk markers for vascular disease in medically uncomplicated overweight or obese subjects: a four week randomized placebo-controlled trial. Atherosclerosis. (2016) 246:169–76. 10.1016/j.atherosclerosis.2016.01.004 26797134

[65] Gutierrez A Duran-Valdez E Robinson I de Serna D Schade D. Does short-term vitamin C reduce cardiovascular risk in type 2 diabetes? Endocr Pract. (2013) 19:785–91. 10.4158/EP12431.OR 23757614

[66] Dewell A Tsao P Rigdon J Gardner CD Antioxidants from diet or supplements do not alter inflammatory markers in adults with cardiovascular disease risk. a pilot randomized controlled trial. Nutr Res. (2018) 50:63–72. 10.1016/j.nutres.2017.10.017 29540273 PMC5858717

[67] Beilfuss J Berg V Sneve M Jorde R Kamycheva E. Effects of a 1-year supplementation with cholecalciferol on interleukin-6, tumor necrosis factor-alpha and insulin resistance in overweight and obese subjects. Cytokine. (2012) 60:870–4. 10.1016/j.cyto.2012.07.032 22925537

[68] Tabesh M Azadbakht L Faghihimani E Tabesh M Esmaillzadeh A. Calcium-vitamin D cosupplementation influences circulating inflammatory biomarkers and adipocytokines in vitamin D-insufficient diabetics: a randomized controlled clinical trial. J Clin Endocrinol Metab. (2014) 99:E2485–93. 10.1210/jc.2014-1977 25215557

[69] Schleithoff SS Zittermann A Tenderich G Berthold HK Stehle P Koerfer R. Vitamin D supplementation improves cytokine profiles in patients with congestive heart failure: a double-blind, randomized, placebo-controlled trial. Am J Clin Nutr. (2006) 83:754–9. 10.1093/ajcn/83.4.754 16600924

[70] Mousa A Naderpoor N Johnson J Sourris K de Courten MPJ Wilson K. Effect of vitamin D supplementation on inflammation and nuclear factor kappa-B activity in overweight/obese adults: a randomized placebo-controlled trial. Sci Rep. (2017) 7:15154. 10.1038/s41598-017-15264-1 29123173 PMC5680306

[71] Waterhouse M Tran B Ebeling PR English DR Lucas RM Venn AJ. Effect of vitamin D supplementation on selected inflammatory biomarkers in older adults: a secondary analysis of data from a randomised, placebo-controlled trial. Br J Nutr. (2015) 114:693–9. 10.1017/S0007114515002366 26206095

[72] Plantinga Y Ghiadoni L Magagna A Giannarelli C Franzoni F Taddei S. Supplementation with vitamins C and E improves arterial stiffness and endothelial function in essential hypertensive patients. Am J Hypertens. (2007) 20:392–7. 10.1016/j.amjhyper.2006.09.021 17386345

[73] Magliano D McNeil J Branley P Shiel L Demos L Wolfe R. The Melbourne Atherosclerosis Vitamin E Trial. (MAVET): a study of high dose vitamin E in smokers. Eur J Cardiovasc Prev Rehabil. (2006) 13:341–7. 10.1097/00149831-200606000-00008 16926662

[74] Devaraj S Tang R Adams-Huet B Harris A Seenivasan T de Lemos JA. Effect of high-dose alpha-tocopherol supplementation on biomarkers of oxidative stress and inflammation and carotid atherosclerosis in patients with coronary artery disease. Am J Clin Nutr. (2007) 86:1392–8. 10.1093/ajcn/86.5.1392 17991651 PMC2692902

[75] Wu JHY Ward NC Indrawan AP Almeida C-A Hodgson JM Proudfoot JM. Effects of alpha-tocopherol and mixed tocopherol supplementation on markers of oxidative stress and inflammation in type 2 diabetes. Clin Chem. (2007) 53:511–9. 10.1373/clinchem.2006.076992 17272491

[76] Gutierrez AD de Serna GD Robinson I Schade DS 13 Why vitamin E does not prevent atherosclerosis. J Investig Med. (2006) 54:S82.1–S82. 10.2310/6650.2005.X0004.12

[77] Knapen MHJ Braam LAJLM Drummen NE Bekers O Hoeks APG Vermeer C. Menaquinone-7 supplementation improves arterial stiffness in healthy postmenopausal women. A double-blind randomised clinical trial. Thromb Haemost. (2015) 113:1135–44. 10.1160/TH14-08-0675 25694037

[78] Kristensen M Kudsk J Bügel S. Six weeks phylloquinone supplementation produces undesirable effects on blood lipids with no changes in inflammatory and fibrinolytic markers in postmenopausal women. Eur J Nutr. (2008) 47:375–9. 10.1007/s00394-008-0737-4 18807108

[79] Shea MK O'Donnell CJ Hoffmann U Dallal GE Dawson-Hughes B Ordovas JM. Vitamin K supplementation and progression of coronary artery calcium in older men and women. Am J Clin Nutr. (2009) 89:1799–807. 10.3945/ajcn.2008.27338 19386744 PMC2682995

[80] Colmán-Martínez M Martínez-Huélamo M Valderas-Martínez P Arranz-Martínez S Almanza-Aguilera E Corella D. *trans* -Lycopene from tomato juice attenuates inflammatory biomarkers in human plasma samples: An intervention trial. Mol Nutr Food Res. (2017) 61:1600993. 10.1002/mnfr.201600993 28688174

[81] Stonehouse W Brinkworth GD Thompson CH Abeywardena MY. Short term effects of palm-tocotrienol and palm-carotenes on vascular function and cardiovascular disease risk: A randomised controlled trial. Atherosclerosis. (2016) 254:205–14. 10.1016/j.atherosclerosis.2016.10.027 27760402

[82] Coombes JS Sharman JE Fassett RG. Astaxanthin has no effect on arterial stiffness, oxidative stress, or inflammation in renal transplant recipients: a randomized controlled trial. (the XANTHIN trial). Am J Clin Nutr. (2016) 103:283–9. 10.3945/ajcn.115.115477 26675778

[83] Zou Z-Y Xu X-R Lin X-M Zhang H-B Xiao X Ouyang L. Effects of lutein and lycopene on carotid intima–media thickness in Chinese subjects with subclinical atherosclerosis: a randomised, double-blind, placebo-controlled trial. Br J Nutr. (2014) 111:474–80. 10.1017/S0007114513002730 24047757

[84] Lambert C Cubedo J Padró T Sánchez-Hernández J Antonijoan RM Perez A. Phytosterols and Omega 3 supplementation exert novel regulatory effects on metabolic and inflammatory pathways: a proteomic study. Nutrients. (2017) 9:9060599. 10.3390/nu9060599 28608804 PMC5490578

[85] Ho XL Liu JJH Loke WM. Plant sterol-enriched soy milk consumption modulates 5-lipoxygenase, 12-lipoxygenase, and myeloperoxidase activities in healthy adults – a randomized-controlled trial. Free Radic Res. (2016) 50:1396–407. 10.1080/10715762.2016.1252839 27776459

[86] Ho XL Loke WM. Dietary plant sterols supplementation increases *in vivo* nitrite and nitrate production in healthy adults: a randomized, controlled study. J Food Sci. (2017) 82:1750–6. 10.1111/1750-3841.13752 28708316 PMC5601184

[87] Heggen E Kirkhus B Pedersen JI Tonstad S. Effects of margarine enriched with plant sterol esters from rapeseed and tall oils on markers of endothelial function, inflammation and hemostasis. Scand J Clin Lab Invest. (2015) 75:189–92. 10.3109/00365513.2014.992040 25553599

[88] Ras RT Fuchs D Koppenol WP Schalkwijk CG Otten-Hofman A Garczarek U. Effect of a plant sterol-enriched spread on biomarkers of endothelial dysfunction and low-grade inflammation in hypercholesterolaemic subjects. J Nutr Sci. (2016) 5:e44. 10.1017/jns.2016.40 28620471 PMC5465806

[89] Ras RT Fuchs D Koppenol WP Garczarek U Greyling A Keicher C. The effect of a low-fat spread with added plant sterols on vascular function markers: results of the Investigating Vascular Function Effects of Plant Sterols. (INVEST) study. Am J Clin Nutr. (2015) 101:733–41. 10.3945/ajcn.114.102053 25809853 PMC4381780

[90] Macedo RCS Vieira A Marin DP Otton R. Effects of chronic resveratrol supplementation in military firefighters undergo a physical fitness test – A placebo-controlled, double blind study. Chem Biol Interact. (2015) 227:89–95. 10.1016/j.cbi.2014.12.033 25572586

[91] Espinoza JL Trung LQ Inaoka PT Yamada K An DT Mizuno S. The Repeated administration of resveratrol has measurable effects on circulating T-cell subsets in humans. Oxid Med Cell Longev. (2017) 2017:1–10. 10.1155/2017/6781872 28546852 PMC5435979

[92] van der Made SM Plat J Mensink RP Resveratrol does not influence metabolic risk markers related to cardiovascular health in overweight and slightly obese subjects: a randomized, placebo-controlled crossover trial. PLoS ONE. (2015) 10:e0118393 10.1371/journal.pone.0118393 25790328 PMC4366169

[93] Made S Plat J Mensink R. Trans-resveratrol supplementation and endothelial function during the fasting and postprandial phase: a randomized placebo-controlled trial in overweight and slightly obese participants. Nutrients. (2017) 9:596. 10.3390/nu9060596 28604618 PMC5490575

[94] Kitada M Ogura Y Maruki-Uchida H Sai M Suzuki T Kanasaki K. The effect of piceatannol from passion fruit. (*Passiflora edulis*) seeds on metabolic health in humans. Nutrients. (2017) 9:1142. 10.3390/nu9101142 29057795 PMC5691758

[95] Kjær TN Ornstrup MJ Poulsen MM Stødkilde-Jørgensen H Jessen N Jørgensen JOL. No beneficial effects of resveratrol on the metabolic syndrome: a randomized placebo-controlled clinical trial. J Clin Endocrinol Metab. (2017) 102:1642–51. 10.1210/jc.2016-2160 28182820

[96] Bo S Ponzo V Evangelista A Ciccone G Goitre I Saba F. Effects of 6 months of resveratrol versus placebo on pentraxin 3 in patients with type 2 diabetes mellitus: a double-blind randomized controlled trial. Acta Diabetol. (2017) 54:499–507. 10.1007/s00592-017-0977-y 28238190

[97] Bo S Ponzo V Ciccone G Evangelista A Saba F Goitre I. Six months of resveratrol supplementation has no measurable effect in type 2 diabetic patients. A randomized, double blind, placebo-controlled trial. Pharmacol Res. (2016) 111:896–905. 10.1016/j.phrs.2016.08.010 27520400

[98] Seyyedebrahimi S Khodabandehloo H Nasli Esfahani E Meshkani R The effects of resveratrol on markers of oxidative stress in patients with type 2 diabetes: a randomized, double-blind, placebo-controlled clinical trial. Acta Diabetol. (2018) 55:341–53. 10.1007/s00592-017-1098-3 29357033

[99] Imamura H Yamaguchi T Nagayama D Saiki A Shirai K Tatsuno I. Resveratrol ameliorates arterial stiffness assessed by cardio-ankle vascular index in patients with type 2 diabetes mellitus. Int Heart J. (2017) 58:577–583. 10.1536/ihj.16-373 28701674

[100] Chen S Zhao X Ran L Wan J Wang X Qin Y. Resveratrol improves insulin resistance, glucose and lipid metabolism in patients with non-alcoholic fatty liver disease: a randomized controlled trial. Dig Liver Dis. (2015) 47:226–32. 10.1016/j.dld.2014.11.015 25577300

[101] Heebøll S Kreuzfeldt M Hamilton-Dutoit S Kjær Poulsen M Stødkilde-Jørgensen H Møller HJ. Placebo-controlled, randomised clinical trial: high-dose resveratrol treatment for non-alcoholic fatty liver disease. Scand J Gastroenterol. (2016) 51:456–64. 10.3109/00365521.2015.1107620 26784973

[102] Faghihzadeh F Adibi P Rafiei R Hekmatdoost A. Resveratrol supplementation improves inflammatory biomarkers in patients with nonalcoholic fatty liver disease. Nutr Res. (2014) 34:837–843. 10.1016/j.nutres.2014.09.005 25311610

[103] Sathyapalan T Aye M Rigby AS Thatcher NJ Dargham SR Kilpatrick ES. Soy isoflavones improve cardiovascular disease risk markers in women during the early menopause. Nutr Metab Cardiovasc Dis. (2018) 28:691–7. 10.1016/j.numecd.2018.03.007 29739677

[104] Hodis HN Mack WJ Kono N Azen SP Shoupe D Hwang-Levine J. Isoflavone soy protein supplementation and atherosclerosis progression in healthy postmenopausal women. Stroke. (2011) 42:3168–75. 10.1161/STROKEAHA.111.620831 21903957 PMC3202054

[105] Byun M-S Yu O-K Cha Y-S Park T-S. Korean traditional Chungkookjang improves body composition, lipid profiles and atherogenic indices in overweight/obese subjects: a double-blind, randomized, crossover, placebo-controlled clinical trial. Eur J Clin Nutr. (2016) 70:1116–22. 10.1038/ejcn.2016.77 27302672

[106] Back H-I Kim S-R Yang J-A Kim M-G Chae S-W Cha Y-S. Effects of *Chungkookjang* Supplementation on obesity and atherosclerotic indices in overweight/obese subjects: a 12-week, randomized, double-blind, placebo-controlled clinical trial. J Med Food. (2011) 14:532–7. 10.1089/jmf.2010.1199 21434780

[107] Chan Y-H Lau K-K Yiu K-H Li S-W Chan H-T Fong DY-T. Reduction of C-reactive protein with isoflavone supplement reverses endothelial dysfunction in patients with ischaemic stroke. Eur Heart J. (2008) 29:2800–7. 10.1093/eurheartj/ehn409 18812325

[108] Törmälä R Appt S Clarkson TB Groop P-H Rönnback M Ylikorkala O. Equol production capability is associated with favorable vascular function in postmenopausal women using tibolone; no effect with soy supplementation. Atherosclerosis. (2008) 198:174–8. 10.1016/j.atherosclerosis.2007.09.010 17961576

[109] Fuchs D Vafeiadou K Hall WL Daniel H Williams CM Schroot JH. Proteomic biomarkers of peripheral blood mononuclear cells obtained from postmenopausal women undergoing an intervention with soy isoflavones. Am J Clin Nutr. (2007) 86:1369–75. 10.1093/ajcn/86.5.1369 17991648

[110] Brüll V Burak C Stoffel-Wagner B Wolffram S Nickenig G Müller C. No effects of quercetin from onion skin extract on serum leptin and adiponectin concentrations in overweight-to-obese patients with. (pre-) hypertension: a randomized double-blinded, placebo-controlled crossover trial. Eur J Nutr. (2017) 56:2265–75. 10.1007/s00394-016-1267-0 27423432

[111] Dower JI Geleijnse JM Gijsbers L Zock PL Kromhout D Hollman PC. Effects of the pure flavonoids epicatechin and quercetin on vascular function and cardiometabolic health: a randomized, double-blind, placebo-controlled, crossover trial. Am J Clin Nutr. (2015) 101:914–21. 25934864 10.3945/ajcn.114.098590

[112] Pfeuffer M Auinger A Bley U Kraus-Stojanowic I Laue C Winkler P. Effect of quercetin on traits of the metabolic syndrome, endothelial function and inflammation in men with different APOE isoforms. Nutr Metab Cardiovasc Dis. (2013) 23:403–9. 10.1016/j.numecd.2011.08.010 22118955

[113] Huang L-H Liu C-Y Wang L-Y Huang C-J Hsu C-H. Effects of green tea extract on overweight and obese women with high levels of low density-lipoprotein-cholesterol. (LDL-C): a randomised, double-blind, and cross-over placebo-controlled clinical trial. BMC Complement Altern Med. (2018) 18:294. 10.1186/s12906-018-2355-x 30400924 PMC6218972

[114] Venkatakrishnan K Chiu H-F Cheng J-C Chang Y-H Lu Y-Y Han Y-C. Comparative studies on the hypolipidemic, antioxidant and hepatoprotective activities of catechin-enriched green and oolong tea in a double-blind clinical trial. Food Funct. (2018) 9:1205–13. 10.1039/C7FO01449J 29384173

[115] Saarenhovi M Salo P Scheinin M Lehto J Lovró Z Tiihonen K. The effect of an apple polyphenol extract rich in epicatechin and flavan-3-ol oligomers on brachial artery flow-mediated vasodilatory function in volunteers with elevated blood pressure. Nutr J. (2017) 16:73. 10.1186/s12937-017-0291-0 29078780 PMC5660451

[116] Samavat H Newman AR Wang R Yuan J-M Wu AH Kurzer MS. Effects of green tea catechin extract on serum lipids in postmenopausal women: a randomized, placebo-controlled clinical trial. Am J Clin Nutr. (2016) 104:1671–82. 10.3945/ajcn.116.137075 27806972 PMC5118731

[117] Dostal AM Samavat H Espejo L Arikawa AY Stendell-Hollis NR Kurzer MS. Green tea extract and catechol-O-methyltransferase genotype modify fasting serum insulin and plasma adiponectin concentrations in a randomized controlled trial of overweight and obese postmenopausal women. J Nutr. (2016) 146:38–45. 10.3945/jn.115.222414 26581683 PMC4700981

[118] Homayouni F Haidari F Hedayati M Zakerkish M Ahmadi K. Blood pressure lowering and anti-inflammatory effects of hesperidin in type 2 diabetes; a randomized double-blind controlled clinical trial. Phyther Res. (2018) 32:1073–9. 10.1002/ptr.6046 29468764

[119] Homayouni F Haidari F Hedayati M Zakerkish M Ahmadi K. Hesperidin supplementation alleviates oxidative DNA damage and lipid peroxidation in type 2 diabetes: a randomized double-blind placebo-controlled clinical trial. Phyther Res. (2017) 31:1539–45. 10.1002/ptr.5881 28805022

[120] Salden BN Troost FJ de Groot E Stevens YR Garcés-Rimón M Possemiers S. Randomized clinical trial on the efficacy of hesperidin 2S on validated cardiovascular biomarkers in healthy overweight individuals. Am J Clin Nutr. (2016) 104:1523–33. 10.3945/ajcn.116.136960 27797708

[121] Stirban A Nandrean S Götting C Tamler R Pop A Negrean M. Effects of n−3 fatty acids on macro- and microvascular function in subjects with type 2 diabetes mellitus. Am J Clin Nutr. (2010) 91:808–13. 10.3945/ajcn.2009.28374 20071644

[122] Dangardt F Osika W Chen Y Nilsson U Gan L-M Gronowitz E. Omega-3 fatty acid supplementation improves vascular function and reduces inflammation in obese adolescents. Atherosclerosis. (2010) 212:580–5. 10.1016/j.atherosclerosis.2010.06.046 20727522

[123] Rizza S Tesauro M Cardillo C Galli A Iantorno M Gigli F. Fish oil supplementation improves endothelial function in normoglycemic offspring of patients with type 2 diabetes. Atherosclerosis. (2009) 206:569–74. 10.1016/j.atherosclerosis.2009.03.006 19394939 PMC2772138

[124] Haberka M Mizia- Stec K Mizia M Janowska J Gieszczyk K Chmiel A N-3 polyunsaturated fatty acids early supplementation improves ultrasound indices of endothelial function, but not through NO inhibitors in patients with acute myocardial infarction: N-3 PUFA supplementation in acute myocardial infarction. Clin Nutr. (2011) 30:79–85. 10.1016/j.clnu.2010.07.011 20705373

[125] Nozue T Yamamoto S Tohyama S Fukui K Umezawa S Onishi Y. Effects of serum n-3 to n-6 polyunsaturated fatty acids ratios on coronary atherosclerosis in statin-treated patients with coronary artery disease. Am J Cardiol. (2013) 111:6–11. 10.1016/j.amjcard.2012.08.038 23040588

[126] Moss JWE Ramji DP. Nutraceutical therapies for atherosclerosis. Nat Rev Cardiol. (2016) 13:513–32. 10.1038/nrcardio.2016.103 27383080 PMC5228762

[127] Harris WS Mozaffarian D Rimm E Kris-Etherton P Rudel LL Appel LJ. Omega-6 fatty acids and risk for cardiovascular disease a science advisory from the american heart association nutrition subcommittee of the council on nutrition, physical activity, and metabolism; council on cardiovascular nursing; and council on epidemiology and prevention. Circulation. (2009) 119:902–7. 10.1161/CIRCULATIONAHA.108.191627 19171857

[128] Das UN. Essential fatty acids and their metabolites could function as endogenous HMG-CoA reductase and ACE enzyme inhibitors, anti-arrhythmic, anti-hypertensive, anti-atherosclerotic, anti-inflammatory, cytoprotective, and cardioprotective molecules. Lipids Health Dis. (2008) 7:37. 10.1186/1476-511X-7-37 18922179 PMC2576273

[129] Hooper L Al-Khudairy L Abdelhamid AS Rees K Brainard JS Brown TJ. Omega-6 fats for the primary and secondary prevention of cardiovascular disease. Cochrane Database Syst Rev. (2018) 7:CD011094. 10.1002/14651858.CD011094.pub3 30019765 PMC6513455

[130] Haghighatdoost F Nobakht M Gh BF. Effect of conjugated linoleic acid on blood inflammatory markers: a systematic review and meta-analysis on randomized controlled trials. Eur J Clin Nutr. (2018) 72:1071–82. 10.1038/s41430-017-0048-z 29288248

[131] Mazidi M Karimi E Rezaie P Ferns GA. Effects of conjugated linoleic acid supplementation on serum C-reactive protein: a systematic review and meta-analysis of randomized controlled trials. Cardiovasc Ther. (2017) 35:e12275. 10.1111/1755-5922.12275 28556504

[132] Johnson GH Fritsche K. Effect of dietary linoleic acid on markers of inflammation in healthy persons: a systematic review of randomized controlled trials. J Acad Nutr Diet. (2012) 112:1029–41.e15. 10.1016/j.jand.2012.03.029 22889633

[133] Tsimikas S Philis-Tsimikas A Alexopoulos S Sigari F Lee C Reaven PD. LDL Isolated From Greek Subjects on a Typical Diet or From American Subjects on an Oleate-Supplemented Diet Induces Less Monocyte Chemotaxis and Adhesion When Exposed to Oxidative Stress. (1999). Available online at: http://www.atvbaha.org (accessed December 28, 2018). 10.1161/01.atv.19.1.122 9888874

[134] Martinelli N Girelli D Malerba G Guarini P Illig T Trabetti E. FADS genotypes and desaturase activity estimated by the ratio of arachidonic acid to linoleic acid are associated with inflammation and coronary artery disease. Am J Clin Nutr. (2008) 88:941–9. 10.1093/ajcn/88.4.941 18842780

[135] Mathias RA Sergeant S Ruczinski I Torgerson DG Hugenschmidt CE Kubala M. The impact of FADS genetic variants on ω6 polyunsaturated fatty acid metabolism in African Americans. BMC Genet. (2011) 12:50. 10.1186/1471-2156-12-50 21599946 PMC3118962

[136] Pravst I Žmitek K Žmitek J. Coenzyme Q10 contents in foods and fortification strategies. Crit Rev Food Sci Nutr. (2010) 50:269–80. 10.1080/10408390902773037 20301015

[137] Hernández-Camacho JD Bernier M López-Lluch G Navas P. Coenzyme Q10 supplementation in aging and disease. Front Physiol. (2018) 9:44. 10.3389/fphys.2018.00044 29459830 PMC5807419

[138] Mortensen SA Rosenfeldt F Kumar A Dolliner P Filipiak KJ Pella D The effect of coenzyme Q 10 on morbidity and mortality in chronic heart failure. JACC Hear Fail. (2014) 2:641–9. 10.1016/j.jchf.2014.06.008 25282031

[139] Alehagen U Alexander J Aaseth J. Supplementation with selenium and coenzyme Q10 reduces cardiovascular mortality in elderly with low selenium status. a secondary analysis of a randomised clinical trial. PLoS ONE. (2016) 11:e0157541. 10.1371/journal.pone.0157541 27367855 PMC4930181

[140] Zhang S Yang K Zeng L Wu X Huang H. Effectiveness of coenzyme Q10 supplementation for type 2 diabetes mellitus: a systematic review and meta-analysis. Int J Endocrinol. (2018) 2018:1–11. 10.1155/2018/6484839 30305810 PMC6165589

[141] Jorat MV Tabrizi R Mirhosseini N Lankarani KB Akbari M Heydari ST. The effects of coenzyme Q10 supplementation on lipid profiles among patients with coronary artery disease: a systematic review and meta-analysis of randomized controlled trials. Lipids Health Dis. (2018) 17:230. 10.1186/s12944-018-0876-4 30296936 PMC6176512

[142] Flowers N Hartley L Todkill D Stranges S Rees K Co-enzyme Q10 supplementation for the primary prevention of cardiovascular disease. Cochrane Database Syst Rev. (2014) 12:CD010405 10.1002/14651858.CD010405.pub2 PMC9759150 25474484

[143] Gao L Mao Q Cao J Wang Y Zhou X Fan L. Effects of coenzyme Q10 on vascular endothelial function in humans: a meta-analysis of randomized controlled trials. Atherosclerosis. (2012) 221:311–6. 10.1016/j.atherosclerosis.2011.10.027 22088605

[144] Fan L Feng Y Chen G-C Qin L-Q Fu C Chen L-H. Effects of coenzyme Q10 supplementation on inflammatory markers: a systematic review and meta-analysis of randomized controlled trials. Pharmacol Res. (2017) 119:128–36. 10.1016/j.phrs.2017.01.032 28179205

[145] Zhai J Bo Y Lu Y Liu C Zhang L. Effects of coenzyme Q10 on markers of inflammation: a systematic review and meta-analysis. PLoS ONE. (2017) 12:e0170172. 10.1371/journal.pone.0170172 28125601 PMC5268485

[146] Aslani Z Shab-Bidar S Fatahi S Djafarian K. Effect of coenzyme Q10 supplementation on serum of high sensitivity c-reactive protein level in patients with cardiovascular diseases: a systematic review and meta-analysis of randomized controlled trials. Int J Prev Med. (2018) 9:82. 10.4103/ijpvm.IJPVM_263_17 30283614 PMC6151976

[147] Toole JF Malinow MR Chambless LE Spence JD Pettigrew LC Howard VJ. Lowering homocysteine in patients with ischemic stroke to prevent recurrent stroke, myocardial infarction, and death: the vitamin intervention for stroke prevention (VISP) randomized controlled trial. JAMA. (2004) 291:565–75. 10.1001/jama.291.5.565 14762035

[148] Spence JD Bang H Chambless LE Stampfer MJ. Vitamin intervention for stroke prevention trial. Stroke. (2005) 36:2404–9. 10.1161/01.STR.0000185929.38534.f3 16239629

[149] Spence JD. Homocysteine. Stroke. (2006) 37:282–3. 10.1161/01.STR.0000199621.28234.e2 16397178

[150] Wang L Manson JE Song Y Sesso HD. Systematic review: vitamin D and calcium supplementation in prevention of cardiovascular events. Ann Intern Med. (2010) 152:315. 10.7326/0003-4819-152-5-201003020-00010 20194238

[151] Root MM McGinn MC Nieman DC Henson DA Heinz SA Shanely RA. Combined fruit and vegetable intake is correlated with improved inflammatory and oxidant status from a cross-sectional study in a community setting. Nutrients. (2012) 4:29–41. 10.3390/nu4010029 22347616 PMC3277099

[152] Danziger J Young RL Shea MK Tracy RP Ix JH Jenny NS. Vitamin K-Dependent protein activity and incident ischemic cardiovascular disease: the multi-ethnic study of atherosclerosis. Arterioscler Thromb Vasc Biol. (2016) 36:1037–42. 10.1161/ATVBAHA.116.307273 27034472 PMC5844474

[153] Mozos I Marginean O. Links between vitamin D deficiency and cardiovascular diseases. Biomed Res Int. (2015) 2015:1–12. 10.1155/2015/109275 26000280 PMC4427096

[154] Helmersson J Ärnlöv J Larsson A Basu S. Low dietary intake of β-carotene, α-tocopherol and ascorbic acid is associated with increased inflammatory and oxidative stress status in a Swedish cohort. Br J Nutr. (2009) 101:1775. 10.1017/S0007114508147377 19079838

[155] Kheiri B Abdalla A Osman M Ahmed S Hassan M Bachuwa G Vitamin D deficiency and risk of cardiovascular diseases: a narrative review. Clin Hypertens. (2018) 24:9 10.1186/s40885-018-0094-4 29977597 PMC6013996

[156] Wang L Gaziano JM Norkus EP Buring JE Sesso HD. Associations of plasma carotenoids with risk factors and biomarkers related to cardiovascular disease in middle-aged and older women. Am J Clin Nutr. (2008) 88:747–54. 10.1093/ajcn/88.3.747 18779292 PMC2559966

[157] Ozkanlar S Akcay F. Antioxidant vitamins in atherosclerosis–animal experiments and clinical studies. Adv Clin Exp Med. 21:115–23. 23214308

[158] Bleys J Miller ER Pastor-Barriuso R Appel LJ Guallar E. Vitamin-mineral supplementation and the progression of atherosclerosis: a meta-analysis of randomized controlled trials. Am J Clin Nutr. (2006) 84:880–7. 10.1093/ajcn/84.4.880 17023716

[159] Hosseini B Saedisomeolia A Skilton MR. Association between micronutrients intake/status and carotid intima media thickness: a systematic review. J Acad Nutr Diet. (2017) 117:69–82. 10.1016/j.jand.2016.09.031 27863993

[160] Bazzano LA. Folic acid supplementation cardiovascular disease: the state of the art. Am J Med Sci. (2009) 338:48–9. 10.1097/MAJ.0b013e3181aaefd6 19593104

[161] Zhang C Wang Z-Y Qin Y-Y Yu F-F Zhou Y-H. Association between B vitamins supplementation and risk of cardiovascular outcomes: a cumulative meta-analysis of randomized controlled trials. PLoS ONE. (2014) 9:e107060. 10.1371/journal.pone.0107060 25238614 PMC4169527

[162] Li W-F Zhang D-D Xia J-T Wen S-F Guo J Li Z-C. The association between B vitamins supplementation and adverse cardiovascular events: a meta-analysis. Int J Clin Exp Med. (2014) 7:1923–30. 25232372 PMC4161532

[163] Ntaios G Savopoulos C Grekas D Hatzitolios A. The controversial role of B-vitamins in cardiovascular risk: an update. Arch Cardiovasc Dis. (2009) 102:847–54. 10.1016/j.acvd.2009.07.002 19963194

[164] Morris MS Sakakeeny L Jacques PF Picciano MF Selhub J. Vitamin B-6 intake is inversely related to, and the requirement is affected by, inflammation status. J Nutr. (2010) 140:103–10. 10.3945/jn.109.114397 19906811 PMC2793124

[165] Riccioni G Bucciarelli T Mancini B Corradi F Di Ilio C Mattei PA. Antioxidant vitamin supplementation in cardiovascular diseases. Ann Clin Lab Sci. (2007) 37:89–95. 17311876

[166] Ciccone MM Cortese F Gesualdo M Carbonara S Zito A Ricci G. Dietary intake of carotenoids and their antioxidant and anti-inflammatory effects in cardiovascular care. Mediators Inflamm. (2013) 2013:1–11. 10.1155/2013/782137 24489447 PMC3893834

[167] Bjelakovic G Nikolova D Gluud LL Simonetti RG Gluud C Antioxidant supplements for prevention of mortality in healthy participants and patients with various diseases. Cochrane Database Syst Rev. (2012) 14:CD007176 10.1002/14651858.CD007176.pub2 PMC8407395 22419320

[168] Myung S-K Ju W Cho B Oh S-W Park SM Koo B-K. Efficacy of vitamin and antioxidant supplements in prevention of cardiovascular disease: systematic review and meta-analysis of randomised controlled trials. BMJ. (2013) 346:f10. 10.1136/bmj.f10 23335472 PMC3548618

[169] Ye Y Li J Yuan Z. Effect of antioxidant vitamin supplementation on cardiovascular outcomes: a meta-analysis of randomized controlled trials. PLoS ONE. (2013) 8:e56803. 10.1371/journal.pone.0056803 23437244 PMC3577664

[170] Vivekananthan DP Penn MS Sapp SK Hsu A Topol EJ. Use of antioxidant vitamins for the prevention of cardiovascular disease: meta-analysis of randomised trials. Lancet. (2003) 361:2017–23. 10.1016/S0140-6736(03)13637-9 12814711

[171] Jenkins DJA Spence JD Giovannucci EL Kim Y Josse R Vieth R. Supplemental vitamins and minerals for CVD prevention and treatment. J Am Coll Cardiol. (2018) 71:2570–84. 10.1016/j.jacc.2018.04.020 29852980

[172] Soni MG Thurmond TS Miller ER Spriggs T Bendich A Omaye ST. Safety of vitamins and minerals: controversies and perspective. Toxicol Sci. (2010) 118:348–55. 10.1093/toxsci/kfq293 20861067

[173] Rutkowski M Grzegorczyk K. Adverse effects of antioxidative vitamins. Int J Occup Med Environ Health. (2012) 25:105–21. 10.2478/s13382-012-0022-x 22528540

[174] Weber C Erl W Weber K Weber PC. Increased adhesiveness of isolated monocytes to endothelium is prevented by vitamin C intake in smokers. Circulation. (1996) 93:1488–92. 8608614 10.1161/01.cir.93.8.1488

[175] d'Uscio L V Milstien S Richardson D Smith L Katusic ZS. Long-term vitamin C treatment increases vascular tetrahydrobiopterin levels and nitric oxide synthase activity. Circ Res. (2003) 92:88–95. 10.1161/01.RES.0000049166.33035.62 12522125

[176] Mortensen A Lykkesfeldt J. Does vitamin C enhance nitric oxide bioavailability in a tetrahydrobiopterin-dependent manner*? In vitro, in vivo* and clinical studies. Nitric Oxide. (2014) 36:51–7. 10.1016/j.niox.2013.12.001 24333161

[177] Libby P Aikawa M. Vitamin C, collagen, and cracks in the plaque. Circulation. (2002) 105:1396–8. 10.1161/01.CIR.0000012513.58079.EA 11914242

[178] Wallace S Wallace A new look at atherosclerosis repeatable science ushers in a new era of medicine. J Cardiol Curr Resh. (2017) 9:00341 10.15406/jccr.2017.09.00341

[179] Osganian SK Stampfer MJ Rimm E Spiegelman D Hu FB Manson JE. Vitamin C and risk of coronary heart disease in women. J Am Coll Cardiol. (2003) 42:246–52. 10.1016/S0735-1097(03)00575-8 12875759

[180] Lynch SM Gaziano JM Frei B. Ascorbic acid and atherosclerotic cardiovascular disease. Subcell Biochem. (1996) 25:331–67. 10.1007/978-1-4613-0325-1_17 8821982

[181] Aune D Keum N Giovannucci E Fadnes LT Boffetta P Greenwood DC. Dietary intake and blood concentrations of antioxidants and the risk of cardiovascular disease, total cancer, and all-cause mortality: a systematic review and dose-response meta-analysis of prospective studies. Am J Clin Nutr. (2018) 108:1069–91. 10.1093/ajcn/nqy097 30475962 PMC6250988

[182] Lee C-H Chan R Wan H Woo Y-C Cheung C Fong C. Dietary intake of anti-oxidant vitamins A, C, and E Is inversely associated with adverse cardiovascular outcomes in Chinese—A 22-years population-based prospective study. Nutrients. (2018) 10:1664. 10.3390/nu10111664 30400367 PMC6265686

[183] Martín-Calvo N Martínez-González M. Vitamin C intake is inversely associated with cardiovascular mortality in a cohort of spanish graduates: the SUN project. Nutrients. (2017) 9:954. 10.3390/nu9090954 28850099 PMC5622714

[184] Juraschek SP Guallar E Appel LJ Miller ER. Effects of vitamin C supplementation on blood pressure: a meta-analysis of randomized controlled trials. Am J Clin Nutr. (2012) 95:1079–88. 10.3945/ajcn.111.027995 22492364 PMC3325833

[185] Ashor AW Siervo M van der Velde F Willis ND Mathers JC. Systematic review and meta-analysis of randomised controlled trials testing the effects of vitamin C supplementation on blood lipids. Clin Nutr. (2016) 35:626–37. 10.1016/j.clnu.2015.05.021 26164552

[186] Al-Khudairy L Flowers N Wheelhouse R Ghannam O Hartley L Stranges S. Vitamin C supplementation for the primary prevention of cardiovascular disease. Cochrane Database Syst Rev. (2017) 3:CD011114. 10.1002/14651858.CD011114.pub2 28301692 PMC6464316

[187] Cook NR Albert CM Gaziano JM Zaharris E MacFadyen J Danielson E. A randomized factorial trial of vitamins C and E and beta carotene in the secondary prevention of cardiovascular events in women. Arch Intern Med. (2007) 167:1610. 10.1001/archinte.167.15.1610 17698683 PMC2034519

[188] Sesso HD Buring JE Christen WG Kurth T Belanger C MacFadyen J. Vitamins E and C in the prevention of cardiovascular disease in men. JAMA. (2008) 300:2123. 10.1001/jama.2008.600 18997197 PMC2586922

[189] Hercberg S Galan P Preziosi P Bertrais S Mennen L Malvy D. The SU.VI.MAX Study. Arch Intern Med. (2004) 164:2335. 10.1001/archinte.164.21.2335 15557412

[190] Brown BG Zhao X-Q Chait A Fisher LD Cheung MC Morse JS Simvastatin and niacin, antioxidant vitamins, or the combination for the prevention of coronary disease. N Engl J Med. (2001) 345:1583–92. 10.1056/NEJMoa011090 11757504

[191] Ashor AW Lara J Mathers JC Siervo M. Effect of vitamin C on endothelial function in health and disease: a systematic review and meta-analysis of randomised controlled trials. Atherosclerosis. (2014) 235:9–20. 10.1016/j.atherosclerosis.2014.04.004 24792921

[192] Agarwal M Mehta PK Dwyer JH Dwyer KM Shircore AM Nordstrom CK. Differing relations to early atherosclerosis between vitamin C from supplements vs. food in the los angeles atherosclerosis study: a prospective cohort study. Open Cardiovasc Med J. (2012) 6:113–21. 10.2174/1874192401206010113 23002405 PMC3447163

[193] Salonen JT Nyyssönen K Salonen R Lakka HM Kaikkonen J Porkkala-Sarataho E. Antioxidant supplementation in atherosclerosis prevention (ASAP) study: a randomized trial of the effect of vitamins E and C on 3-year progression of carotid atherosclerosis. J Intern Med. (2000) 248:377–86. 10.1046/j.1365-2796.2000.00752.x 11123502

[194] Jorde R Sollid ST Svartberg J Schirmer H Joakimsen RM Njølstad I Vitamin D 20 000 IU per week for five years does not prevent progression from prediabetes to diabetes. J Clin Endocrinol Metab. (2016) 101:1647–55. 10.1210/jc.2015-4013 26829443

[195] Golzarand M Shab-Bidar S Koochakpoor G Speakman J R Djafarian K. Effect of vitamin D3 supplementation on blood pressure in adults: an updated meta-analysis. Nutr Metab Cardiovasc Dis. (2016) 26:663–73. 10.1016/j.numecd.2016.04.011 27287826

[196] Ford JA MacLennan GS Avenell A Bolland M Grey A Witham M. Cardiovascular disease and vitamin D supplementation: trial analysis, systematic review, and meta-analysis. Am J Clin Nutr. (2014) 100:746–55. 10.3945/ajcn.113.082602 25057156

[197] Hsia J Heiss G Ren H Allison M Dolan NC Greenland P. Calcium/Vitamin D supplementation and cardiovascular events. Circulation. (2007) 115:846–54. 10.1161/CIRCULATIONAHA.106.673491 17309935

[198] Bolland MJ Grey A Gamble GD Reid IR The effect of vitamin D supplementation on skeletal, vascular, or cancer outcomes: a trial sequential meta-analysis. Lancet Diabetes Endocrinol. (2014) 2:307–20. 10.1016/S2213-8587(13)70212-2 24703049

[199] Elamin MB Abu Elnour NO Elamin KB Fatourechi MM Alkatib AA Almandoz JP. Vitamin D and cardiovascular outcomes: a systematic review and meta-analysis. J Clin Endocrinol Metab. (2011) 96:1931–42. 10.1210/jc.2011-0398 21677037

[200] Norman PE Powell JT. Vitamin D and cardiovascular disease. Circ Res. (2014) 114:379–93. 10.1161/CIRCRESAHA.113.301241 24436433

[201] Mousa A Naderpoor N Teede H Scragg R de Courten B. Vitamin D supplementation for improvement of chronic low-grade inflammation in patients with type 2 diabetes: a systematic review and meta-analysis of randomized controlled trials. Nutr Rev. (2018) 76:380–94. 10.1093/nutrit/nux077 29490085

[202] Rodriguez AJ Mousa A Ebeling PR Scott D de Courten B. Effects of vitamin D supplementation on inflammatory markers in heart failure: a systematic review and meta-analysis of randomized controlled trials. Sci Rep. (2018) 8:1169. 10.1038/s41598-018-19708-0 29348609 PMC5773527

[203] Jamka M Wozniewicz M Walkowiak J Bogdanski P Jeszka J Stelmach-Mardas M. The effect of vitamin D supplementation on selected inflammatory biomarkers in obese and overweight subjects: a systematic review with meta-analysis. Eur J Nutr. (2016) 55:2163–76. 10.1007/s00394-015-1089-5 26538075

[204] Beveridge LA Khan F Struthers AD Armitage J Barchetta I Bressendorff I. Effect of vitamin D supplementation on markers of vascular function: a systematic review and individual participant meta-analysis. J Am Heart Assoc. (2018) 7:e008273. 10.1161/JAHA.117.008273 29848497 PMC6015391

[205] De Vita F Lauretani F Bauer J Bautmans I Shardell M Cherubini A. Relationship between vitamin D and inflammatory markers in older individuals. Age. (Omaha). (2014) 36:9694. 10.1007/s11357-014-9694-4 25086618 PMC4150893

[206] Laird E McNulty H Ward M Hoey L McSorley E Wallace JMW. Vitamin D deficiency is associated with inflammation in older Irish adults. J Clin Endocrinol Metab. (2014) 99:1807–15. 10.1210/jc.2013-3507 24606079

[207] Liefaard MC Ligthart S Vitezova A Hofman A Uitterlinden AG Kiefte-de Jong JC. Vitamin D and C-reactive protein: a mendelian randomization study. PLoS ONE. (2015) 10:e0131740. 10.1371/journal.pone.0131740 26147588 PMC4492676

[208] Zittermann A. Vitamin D in preventive medicine: are we ignoring the evidence? Br J Nutr. (2003) 89:552–72. 10.1079/BJN2003837 12720576

[209] Fantuzzi G Faggioni R. Leptin in the regulation of immunity, inflammation, and hematopoiesis. J Leukoc Biol. (2000) 68:437–46. 11037963

[210] Stokić E Kupusinac A Tomic-Naglic D Smiljenic D Kovacev-Zavisic B Srdic-Galic B. Vitamin D and dysfunctional adipose tissue in obesity. Angiology. (2015) 66:613–8. 10.1177/0003319714543512 25053676

[211] Vaidya A Williams JS Forman JP. The independent association between 25-hydroxyvitamin D and adiponectin and its relation with BMI in two large cohorts: the NHS and the HPFS. Obesity. (2012) 20:186–91. 10.1038/oby.2011.210 21760630 PMC3461263

[212] Ilahi M Armas LA Heaney RP. Pharmacokinetics of a single, large dose of cholecalciferol. Am J Clin Nutr. (2008) 87:688–91. 10.1093/ajcn/87.3.688 18326608

[213] Jiang W-L Gu H-B Zhang Y-F Xia Q-Q Qi J Chen J-C. Vitamin D supplementation in the treatment of chronic heart failure: a meta-analysis of randomized controlled trials. Clin Cardiol. (2016) 39:56–61. 10.1002/clc.22473 26415519 PMC6490747

[214] Abou-Raya A Abou-Raya S Helmii M Roux-Lombard P Meyer O Dayer J. The effect of vitamin D supplementation on inflammatory and hemostatic markers and disease activity in patients with systemic lupus erythematosus: a randomized placebo-controlled trial. J Rheumatol. (2013) 40:265–72. 10.3899/jrheum.111594 23204220

[215] Nicholson I Dalzell AM El-Matary W. Vitamin D as a therapy for colitis: a systematic review. J Crohn's Colitis. (2012) 6:405–11. 10.1016/j.crohns.2012.01.007 22398085

[216] Rezk NASA Aly NYA Hewidy AAH Effect of vitamin D replacement in chronic obstructive pulmonary disease patients with vitamin D deficiency. Egypt J Chest Dis Tuberc. (2015) 64:353–7. 10.1016/j.ejcdt.2015.01.002

[217] Sandmand M Bruunsgaard H Kemp K Andersen-Ranberg K Pedersen AN Skinhøj P. Is ageing associated with a shift in the balance between Type 1 and Type 2 cytokines in humans? Clin Exp Immunol. (2002) 127:107–14. 10.1046/j.1365-2249.2002.01736.x 11882040 PMC1906284

[218] Deng Y Jing Y Campbell AE Gravenstein S. Age-related impaired type 1 T cell responses to influenza: reduced activation *ex vivo*, decreased expansion in CTL culture *in vitro*, and blunted response to influenza vaccination *in vivo* in the elderly. J Immunol. (2004) 172:3437–46. 10.4049/jimmunol.172.6.3437 15004143

[219] Looney RJ Falsey AR Walsh E Campbell D. Effect of aging on cytokine production in response to respiratory syncytial virus infection. J Infect Dis. (2002) 185:682–5. 10.1086/339008 11865426

[220] Devaraj S Jialal I. Alpha-tocopherol decreases tumor necrosis factor-alpha mRNA and protein from activated human monocytes by inhibition of 5-lipoxygenase. Free Radic Biol Med. (2005) 38:1212–20. 10.1016/j.freeradbiomed.2005.01.009 15808419

[221] Belisle SE Leka LS Dallal GE Jacques PF Delgado-Lista J Ordovas JM. Cytokine response to vitamin E supplementation is dependent on pre-supplementation cytokine levels. Biofactors. (2008) 33:191–200. 10.1002/biof.5520330305 19478423 PMC2769508

[222] Singh U Devaraj S Jialal I. Vitamin E, oxidative stress, and inflammation. Annu Rev Nutr. (2005) 25:151–74. 10.1146/annurev.nutr.24.012003.132446 16011463

[223] Cordero Z Drogan D Weikert C Boeing H. Vitamin E and risk of cardiovascular diseases: a review of epidemiologic and clinical trial studies. Crit Rev Food Sci Nutr. (2010) 50:420–40. 10.1080/10408390802304230 20373188

[224] Saboori S Shab-Bidar S Speakman JR Yousefi Rad E Djafarian K. Effect of vitamin E supplementation on serum C-reactive protein level: a meta-analysis of randomized controlled trials. Eur J Clin Nutr. (2015) 69:867–73. 10.1038/ejcn.2014.296 25669317

[225] Ashor AW Siervo M Lara J Oggioni C Afshar S Mathers JC. Effect of vitamin C and vitamin E supplementation on endothelial function: a systematic review and meta-analysis of randomised controlled trials. Br J Nutr. (2015) 113:1182–94. 10.1017/S0007114515000227 25919436

[226] Shekelle PG Morton SC Jungvig LK Udani J Spar M Tu W. Effect of supplemental vitamin E for the prevention and treatment of cardiovascular disease. J Gen Intern Med. (2004) 19:380–9. 10.1111/j.1525-1497.2004.30090.x 15061748 PMC1492195

[227] Curtis AJ Bullen M Piccenna L McNeil JJ. Vitamin E supplementation and mortality in healthy people: a meta-analysis of randomised controlled trials. Cardiovasc Drugs Ther. (2014) 28:563–73. 10.1007/s10557-014-6560-7 25398301

[228] Miller ER Pastor-Barriuso R Dalal D Riemersma RA Appel LJ Guallar E. Meta-analysis: high-dosage vitamin E supplementation may increase all-cause mortality. Ann Intern Med. (2005) 142:37–46. 10.7326/0003-4819-142-1-200501040-00110 15537682

[229] Schwab S Zierer A Schneider A Heier M Koenig W Kastenmüller G. Vitamin E supplementation is associated with lower levels of C-reactive protein only in higher dosages and combined with other antioxidants: the Cooperative Health Research in the Region of Augsburg. (KORA) F4 study. Br J Nutr. (2015) 113:1782–91. 10.1017/S0007114515000902 25895432

[230] Freedman JE Keaney JF. Vitamin E inhibition of platelet aggregation is independent of antioxidant activity. J Nutr. (2001) 131:374S−7S. 10.1093/jn/131.2.374S 11160564

[231] Carr BR Khan N Adams-Huet B Kakarla N Havelock JC Gell J. Effect of vitamin E supplementation with and without hormone therapy on circulatory inflammatory markers in postmenopausal women. Fertil Steril. (2006) 85:667–73. 10.1016/j.fertnstert.2005.08.030 16500336

[232] van Tits LJ Demacker PN de Graaf J Hak-Lemmers HL Stalenhoef AF. α-Tocopherol supplementation decreases production of superoxide and cytokines by leukocytes *ex vivo* in both normolipidemic and hypertriglyceridemic individuals. Am J Clin Nutr. (2000) 71:458–64. 10.1093/ajcn/71.2.458 10648258

[233] Harshman SG Shea MK. The Role of Vitamin K in chronic aging diseases: inflammation, cardiovascular disease, and osteoarthritis. Curr Nutr Rep. (2016) 5:90–98. 10.1007/s13668-016-0162-x 27648390 PMC5026413

[234] van Ballegooijen AJ Beulens JW. The Role of Vitamin K status in cardiovascular health: evidence from observational and clinical studies. Curr Nutr Rep. (2017) 6:197–205. 10.1007/s13668-017-0208-8 28944098 PMC5585988

[235] Ohsaki Y Shirakawa H Hiwatashi K Furukawa Y Mizutani T Komai M. Vitamin K suppresses lipopolysaccharide-induced inflammation in the rat. Biosci Biotechnol Biochem. (2006) 70:926–32. 10.1271/bbb.70.926 16636460

[236] Ohsaki Y Shirakawa H Miura A Giriwono PE Sato S Ohashi A. Vitamin K suppresses the lipopolysaccharide-induced expression of inflammatory cytokines in cultured macrophage-like cells via the inhibition of the activation of nuclear factor κB through the repression of IKKα/β phosphorylation. J Nutr Biochem. (2010) 21:1120–6. 10.1016/j.jnutbio.2009.09.011 20149620

[237] Geleijnse JM Vermeer C Grobbee DE Schurgers LJ Knapen MHJ van der Meer IM. Dietary intake of menaquinone is associated with a reduced risk of coronary heart disease: the rotterdam study. J Nutr. (2004) 134:3100–5. 10.1093/jn/134.11.3100 15514282

[238] Gast GCM de Roos NM Sluijs I Bots ML Beulens JWJ Geleijnse JM. A high menaquinone intake reduces the incidence of coronary heart disease. Nutr Metab Cardiovasc Dis. (2009) 19:504–10. 10.1016/j.numecd.2008.10.004 19179058

[239] Shea MK Holden RM. Vitamin K Status and Vascular calcification: evidence from observational and clinical studies. Adv Nutr. (2012) 3:158–65. 10.3945/an.111.001644 22516723 PMC3648716

[240] Shea MK Cushman M Booth SL Burke GL Chen H Kritchevsky SB. Associations between vitamin K status and haemostatic and inflammatory biomarkers in community-dwelling adults. the multi-ethnic study of atherosclerosis. Thromb Haemost. (2014) 112:438–44. 10.1160/TH13-12-1003 24849546 PMC4152396

[241] Juanola-Falgarona M Salas-Salvadó J Estruch R Portillo MP Casas R Miranda J. Association between dietary phylloquinone intake and peripheral metabolic risk markers related to insulin resistance and diabetes in elderly subjects at high cardiovascular risk. Cardiovasc Diabetol. (2013) 12:7. 10.1186/1475-2840-12-7 23298335 PMC3558443

[242] Shea MK Booth SL Massaro JM Jacques PF D'Agostino RB Dawson-Hughes B. Vitamin K and vitamin D status: associations with inflammatory markers in the Framingham Offspring Study. Am J Epidemiol. (2008) 167:313–20. 10.1093/aje/kwm306 18006902 PMC3151653

[243] Shea MK Booth SL Miller ME Burke GL Chen H Cushman M. Association between circulating vitamin K1 and coronary calcium progression in community-dwelling adults: the Multi-Ethnic Study of Atherosclerosis. Am J Clin Nutr. (2013) 98:197–208. 10.3945/ajcn.112.056101 23719555 PMC3683819

[244] Verma H Garg R. Effect of Vitamin K Supplementation on cardiometabolic risk factors: a systematic review and meta-analysis. Endocrine Metab Immune Disord - Drug Targets. (2019) 19:13–25. 10.2174/1871530318666180703125007 29968548

[245] Suksomboon N Poolsup N Darli Ko Ko H. Effect of vitamin K supplementation on insulin sensitivity: a meta-analysis. Diabetes Metab Syndr Obes. (2017) 10:169–77. 10.2147/DMSO.S137571 28496349 PMC5422317

[246] Shahdadian F Mohammadi H Rouhani MH. Effect of Vitamin K Supplementation on glycemic control: a systematic review and meta-analysis of clinical trials. Horm Metab Res. (2018) 50:227–35. 10.1055/s-0044-100616 29523009

[247] Shea MK O'Donnell CJ Vermeer C Magdeleyns EJP Crosier MD Gundberg CM Circulating uncarboxylated matrix gla protein is associated with vitamin K nutritional status, but not coronary artery calcium, in older adults. J Nutr. (2011) 141:1529–34. 10.3945/jn.111.139634 21628633 PMC3138643

[248] Reddi K Henderson B Meghji S Wilson M Poole S Hopper C. Interleukin 6 production by lipopolysaccharide-stimulated human fibroblasts is potently inhibited by Naphthoquinone. (vitamin K) compounds. Cytokine. (1995) 7:287–90. 10.1006/cyto.1995.0034 7640347

[249] Eichbaum FW Slemer O Zyngier SB. Anti-inflammatory effect of warfarin and vitamin K1. Naunyn Schmiedebergs Arch Pharmacol. (1979) 307:185–90. 10.1007/BF00498462 573373

[250] Gammone MA Riccioni G D'Orazio N. Carotenoids: potential allies of cardiovascular health? Food Nutr Res. (2015) 59:26762. 10.3402/fnr.v59.26762 25660385 PMC4321000

[251] Saini RK Nile SH Park SW. Carotenoids from fruits and vegetables: chemistry, analysis, occurrence, bioavailability and biological activities. Food Res Int. (2015) 76:735–50. 10.1016/j.foodres.2015.07.047 28455059

[252] Skibsted LH Carotenoids in antioxidant networks. colorants or radical scavengers. J Agric Food Chem. (2012) 60:2409–17. 10.1021/jf2051416 22339058

[253] Stephensen C Provitamin A Carotenoids and Immune Function. In: Tanumihardjo S editor. Carotenoids and Human Health. New York, NY: Humana Press, 261–70.

[254] Kaulmann A Bohn T. Carotenoids, inflammation, and oxidative stress—implications of cellular signaling pathways and relation to chronic disease prevention. Nutr Res. (2014) 34:907–29. 10.1016/j.nutres.2014.07.010 25134454

[255] Cheng HM Koutsidis G Lodge JK Ashor A Siervo M Lara J. Tomato and lycopene supplementation and cardiovascular risk factors: a systematic review and meta-analysis. Atherosclerosis. (2017) 257:100–8. 10.1016/j.atherosclerosis.2017.01.009 28129549

[256] Leermakers ET Darweesh SK Baena CP Moreira EM Melo van Lent D Tielemans MJ The effects of lutein on cardiometabolic health across the life course: a systematic review and meta-analysis1,2. Am J Clin Nutr. (2016) 103:481–94. 10.3945/ajcn.115.120931 26762372

[257] Li X Xu J. Dietary and circulating lycopene and stroke risk: a meta-analysis of prospective studies. Sci Rep. (2014) 4:5031. 10.1038/srep05031 24848940 PMC5381376

[258] Song B Liu K Gao Y Zhao L Fang H Li Y. Lycopene and risk of cardiovascular diseases: A meta-analysis of observational studies. Mol Nutr Food Res. (2017) 61:1601009. 10.1002/mnfr.201601009 28318092

[259] Preedy VR Vitamin A and Carotenoids : Chemistry, Analysis, Function and Effects. Royal Society of Chemistry, (2012). Available online at: https://books.google.es/books/about/Vitamin_A_and_Carotenoids.html?id=ojNqPbqgepsC&redir_esc=y (accessed March 22, 2019).

[260] He W-S Zhu H Chen Z-Y. Plant Sterols: chemical and enzymatic structural modifications and effects on their cholesterol-lowering activity. J Agric Food Chem. (2018) 66:3062. 10.1021/acs.jafc.8b00059 29521096

[261] Gylling H Simonen P Gylling H Simonen P. Phytosterols, phytostanols, and lipoprotein metabolism. Nutrients. (2015) 7:7965–77. 10.3390/nu7095374 26393644 PMC4586569

[262] Wang M Huang W Hu Y Zhang L Shao Y Wang M. Phytosterol profiles of common foods and estimated natural intake of different structures and forms in China. J Agric Food Chem. (2018) 66:2669–76. 10.1021/acs.jafc.7b05009 29397719

[263] Richelle M Enslen M Hager C Groux M Tavazzi I Godin J-P. Both free and esterified plant sterols reduce cholesterol absorption and the bioavailability of β-carotene and α-tocopherol in normocholesterolemic humans. Am J Clin Nutr. (2004) 80:171–7. 10.1093/ajcn/80.1.171 15213045

[264] Ras RT van der Schouw YT Trautwein EA Sioen I Dalmeijer GW Zock PL. Intake of phytosterols from natural sources and risk of cardiovascular disease in the European Prospective Investigation into Cancer and Nutrition-the Netherlands. (EPIC-NL) population. Eur J Prev Cardiol. (2015) 22:1067–75. 10.1177/2047487314554864 25305273

[265] Phillips KM Tarragó-Trani MT Stewart KK Phytosterol content of experimental diets differing in fatty acid composition. Food Chem. (1999) 64:415–422. 10.1016/S0308-8146(98)00090-9

[266] Srigley CT Hansen SL Smith SA Abraham A Bailey E Chen X Sterols and stanols in foods and dietary supplements containing added phytosterols: a collaborative study. J Am Oil Chem Soc. (2018) 95:247–57. 10.1002/aocs.12011

[267] Gylling H Plat J Turley S Ginsberg HN Ellegård L Jessup W. Plant sterols and plant stanols in the management of dyslipidaemia and prevention of cardiovascular disease. Atherosclerosis. (2014) 232:346–60. 10.1016/j.atherosclerosis.2013.11.043 24468148

[268] Rocha VZ Ras RT Gagliardi AC Mangili LC Trautwein EA Santos RD. Effects of phytosterols on markers of inflammation: A systematic review and meta-analysis. Atherosclerosis. (2016) 248:76–83. 10.1016/j.atherosclerosis.2016.01.035 26987068

[269] Han S Jiao J Xu J Zimmermann D Actis-Goretta L Guan L. Effects of plant stanol or sterol-enriched diets on lipid profiles in patients treated with statins: systematic review and meta-analysis. Sci Rep. (2016) 6:31337. 10.1038/srep31337 27539156 PMC4990897

[270] Ras RT Geleijnse JM Trautwein EA. LDL-cholesterol-lowering effect of plant sterols and stanols across different dose ranges: a meta-analysis of randomised controlled studies. Br J Nutr. (2014) 112:214–9. 10.1017/S0007114514000750 24780090 PMC4071994

[271] Amir Shaghaghi M Abumweis SS Jones PJH. Cholesterol-lowering efficacy of plant sterols/stanols provided in capsule and tablet formats: results of a systematic review and meta-analysis. J Acad Nutr Diet. (2013) 113:1494–503. 10.1016/j.jand.2013.07.006 24144075

[272] St-Onge M-P Jones PJH. Phytosterols and human lipid metabolism: efficacy, safety, and novel foods. Lipids. (2003) 38:367–75. 10.1007/s11745-003-1071-3 12848281

[273] Sirerol JA Rodríguez ML Mena S Asensi MA Estrela JM Ortega AL. Role of natural stilbenes in the prevention of cancer. Oxid Med Cell Longev. (2016) 2016:1–15. 10.1155/2016/3128951 26798416 PMC4698548

[274] Neveu V Perez-Jimenez J Vos F Crespy V du Chaffaut L Mennen L. Phenol-Explorer: an online comprehensive database on polyphenol contents in foods. Database. (2010) 2010:bap024-bap024. 10.1093/database/bap024 20428313 PMC2860900

[275] Berman AY Motechin RA Wiesenfeld MY Holz MK. The therapeutic potential of resveratrol: a review of clinical trials. NPJ Precis Oncol. (2017) 1:35. 10.1038/s41698-017-0038-6 28989978 PMC5630227

[276] Liu Y Ma W Zhang P He S Huang D. Effect of resveratrol on blood pressure: A meta-analysis of randomized controlled trials. Clin Nutr. (2015) 34:27–34. 10.1016/j.clnu.2014.03.009 24731650

[277] Hausenblas HA Schoulda JA Smoliga JM. Resveratrol treatment as an adjunct to pharmacological management in type 2 diabetes mellitus-systematic review and meta-analysis. Mol Nutr Food Res. (2015) 59:147–59. 10.1002/mnfr.201400173 25138371

[278] Feringa HHH Laskey DA Dickson JE Coleman CI. The effect of grape seed extract on cardiovascular risk markers: a meta-analysis of randomized controlled trials. J Am Diet Assoc. (2011) 111:1173–81. 10.1016/j.jada.2011.05.015 21802563

[279] Sahebkar A Serban C Ursoniu S Wong ND Muntner P Graham IM. Lack of efficacy of resveratrol on C-reactive protein and selected cardiovascular risk factors — Results from a systematic review and meta-analysis of randomized controlled trials. Int J Cardiol. (2015) 189:47–55. 10.1016/j.ijcard.2015.04.008 25885871

[280] Haghighatdoost F Hariri M. Effect of resveratrol on lipid profile: an updated systematic review and meta-analysis on randomized clinical trials. Pharmacol Res. (2018) 129:141–50. 10.1016/j.phrs.2017.12.033 29305228

[281] Kappelle PJWH Dullaart RPF van Beek AP Hillege HL Wolffenbuttel BHR. The plasma leptin/adiponectin ratio predicts first cardiovascular event in men: a prospective nested case–control study. Eur J Intern Med. (2012) 23:755–9. 10.1016/j.ejim.2012.06.013 22819464

[282] Mohammadi-Sartang M Mazloom Z Sohrabi Z Sherafatmanesh S Barati-Boldaji R. Resveratrol supplementation and plasma adipokines concentrations? A systematic review and meta-analysis of randomized controlled trials. Pharmacol Res. (2017) 117:394–405. 10.1016/j.phrs.2017.01.012 28089943

[283] Ambigaipalan P Phenolics and polyphenolics in foods, beverages and spices: antioxidant activity and health effects – A review. J Funct Foods. (2015) 18:820–897. 10.1016/j.jff.2015.06.018

[284] Rodriguez-Mateos A Vauzour D Krueger CG Shanmuganayagam D Reed J Calani L. Bioavailability, bioactivity and impact on health of dietary flavonoids and related compounds: an update. Arch Toxicol. (2014) 88:1803–53. 10.1007/s00204-014-1330-7 25182418

[285] Grosso G Micek A Godos J Pajak A Sciacca S Galvano F. Dietary flavonoid and lignan intake and mortality in prospective cohort studies: systematic review and dose-response meta-analysis. Am J Epidemiol. (2017) 185:1304–16. 10.1093/aje/kww207 28472215

[286] Kim Y Je Y. Flavonoid intake and mortality from cardiovascular disease and all causes: A meta-analysis of prospective cohort studies. Clin Nutr ESPEN. (2017) 20:68–77. 10.1016/j.clnesp.2017.03.004 29072172

[287] Liu X Liu Y Huang Y Yu H Yuan S Tang B. Dietary total flavonoids intake and risk of mortality from all causes and cardiovascular disease in the general population: A systematic review and meta-analysis of cohort studies. Mol Nutr Food Res. (2017) 61:1601003. 10.1002/mnfr.201601003 28054441

[288] Wang X Ouyang YY Liu J Zhao G. Flavonoid intake and risk of CVD: a systematic review and meta-analysis of prospective cohort studies. Br J Nutr. (2014) 111:1–11. 10.1017/S000711451300278X 23953879

[289] Jiang W Wei H He B. Dietary flavonoids intake and the risk of coronary heart disease: A dose-response meta-analysis of 15 prospective studies. Thromb Res. (2015) 135:459–463. 10.1016/j.thromres.2014.12.016 25555317

[290] Tang Z Li M Zhang X Hou W. Dietary flavonoid intake and the risk of stroke: a dose-response meta-analysis of prospective cohort studies. BMJ Open. (2016) 6:e008680. 10.1136/bmjopen-2015-008680 27279473 PMC4908865

[291] Keinan-Boker L Peeters P Mulligan A Navarro C Slimani N Mattisson I. Soy product consumption in 10 European countries: the European Prospective Investigation into Cancer and Nutrition. (EPIC) study. Public Health Nutr. (2002) 5:1217. 10.1079/PHN2002400 12639228

[292] Piscopo A De Bruno A Zappia A Ventre C Poiana M. Characterization of monovarietal olive oils obtained from mills of Calabria region. (Southern Italy). Food Chem. (2016) 213:313–8. 10.1016/j.foodchem.2016.06.080 27451186

[293] Zhang Y-B Chen W-H Guo J-J Fu Z-H Yi C Zhang M. Soy isoflavone supplementation could reduce body weight and improve glucose metabolism in non-Asian postmenopausal women—A meta-analysis. Nutrition. (2013) 29:8–14. 10.1016/j.nut.2012.03.019 22858192

[294] Simental-Mendía LE Gotto AM Atkin SL Banach M Pirro M Sahebkar A. Effect of soy isoflavone supplementation on plasma lipoprotein(a) concentrations: a meta-analysis. J Clin Lipidol. (2018) 12:16–24. 10.1016/j.jacl.2017.10.004 29129666

[295] Sekikawa A Ihara M Lopez O Kakuta C Lopresti B Higashiyama A. Effect of S-equol and soy isoflavones on heart and brain. Curr Cardiol Rev. (2018) 15:114–35. 10.2174/1573403X15666181205104717 30516108 PMC6520578

[296] Mangano KM Hutchins-Wiese HL Kenny AM Walsh SJ Abourizk RH Bruno RS Soy proteins and isoflavones reduce interleukin-6 but not serum lipids in older women: a randomized controlled trial. Nutr Res. (2013) 33:1026–33. 10.1016/j.nutres.2013.08.009 24267042 PMC4452619

[297] Ushioda M Makita K Takamatsu K Horiguchi F Aoki D. Serum Lipoprotein(a) Dynamics before/after menopause and long-term effects of hormone replacement therapy on lipoprotein(a) levels in middle-aged and older Japanese women. Horm Metab Res. (2006) 38:581–6. 10.1055/s-2006-950504 16981140

[298] Menezes R Rodriguez-Mateos A Kaltsatou A González-Sarrías A Greyling A Giannaki C. Impact of flavonols on cardiometabolic biomarkers: a meta-analysis of randomized controlled human trials to explore the role of inter-individual variability. Nutrients. (2017) 9:E117. 10.3390/nu9020117 28208791 PMC5331548

[299] Erlund I Review of the flavonoids quercetin, hesperetin, and naringenin. Dietary sources, bioactivities, bioavailability, and epidemiology. Nutr Res. (2004) 24:851–74. 10.1016/j.nutres.2004.07.005

[300] Serban M-C Sahebkar A Zanchetti A Mikhailidis DP Howard G Antal D. Effects of quercetin on blood pressure: a systematic review and meta-analysis of randomized controlled trials. J Am Heart Assoc. (2016) 5:e002713. 10.1161/JAHA.115.002713 27405810 PMC5015358

[301] Mohammadi-Sartang M Mazloom Z Sherafatmanesh S Ghorbani M Firoozi D. Effects of supplementation with quercetin on plasma C-reactive protein concentrations: a systematic review and meta-analysis of randomized controlled trials. Eur J Clin Nutr. (2017) 71:1033–9. 10.1038/ejcn.2017.55 28537580

[302] Peluso I Raguzzini A Serafini M. Effect of flavonoids on circulating levels of TNF-α and IL-6 in humans: a systematic review and meta-analysis. Mol Nutr Food Res. (2013) 57:784–801. 10.1002/mnfr.201200721 23471810

[303] Sahebkar A. Effects of quercetin supplementation on lipid profile: a systematic review and meta-analysis of randomized controlled trials. Crit Rev Food Sci Nutr. (2017) 57:666–76. 10.1080/10408398.2014.948609 25897620

[304] Brüll V Burak C Stoffel-Wagner B Wolffram S Nickenig G Müller C Acute intake of quercetin from onion skin extract does not influence postprandial blood pressure and endothelial function in overweight-to-obese adults with hypertension: a randomized, double-blind, placebo-controlled, crossover trial. Eur J Nutr. (2017) 56:1347–57. 10.1007/s00394-016-1185-1 26924303

[305] Hoek-van den Hil EF van Schothorst EM van der Stelt I Swarts HJM van Vliet M Amolo T. Direct comparison of metabolic health effects of the flavonoids quercetin, hesperetin, epicatechin, apigenin and anthocyanins in high-fat-diet-fed mice. Genes Nutr. (2015) 10:469. 10.1007/s12263-015-0469-z 26022682 PMC4447677

[306] Ried K Fakler P Stocks NP. Effect of cocoa on blood pressure. Cochr Database Syst Rev. (2017) 4:CD008893. 10.1002/14651858.CD008893.pub3 28439881 PMC6478304

[307] Lin X Zhang I Li A Manson JE Sesso HD Wang L Liu S. Cocoa flavanol intake and biomarkers for cardiometabolic health: a systematic review and meta-analysis of randomized controlled trials. J Nutr. (2016) 146:2325–33. 10.3945/jn.116.237644 27683874 PMC5086796

[308] Hooper L Kay C Abdelhamid A Kroon PA Cohn JS Rimm EB. Effects of chocolate, cocoa, and flavan-3-ols on cardiovascular health: a systematic review and meta-analysis of randomized trials. Am J Clin Nutr. (2012) 95:740–51. 10.3945/ajcn.111.023457 22301923

[309] Khalesi S Sun J Buys N Jamshidi A Nikbakht-Nasrabadi E Khosravi-Boroujeni H. Green tea catechins and blood pressure: a systematic review and meta-analysis of randomised controlled trials. Eur J Nutr. (2014) 53:1299–311. 10.1007/s00394-014-0720-1 24861099

